# H2B oncohistones cause homologous recombination defect and genomic instability through reducing H2B monoubiquitination in *Schizosaccharomyces pombe*

**DOI:** 10.1016/j.jbc.2024.107345

**Published:** 2024-05-07

**Authors:** Bingxin Qin, Guangchun Lu, Xuejin Chen, Chenhua Zheng, Huanteng Lin, Qi Liu, Jinjie Shang, Gang Feng

**Affiliations:** 1 Jiangsu Key Laboratory for Microbes and Functional Genomics, College of Life Sciences, Nanjing Normal University, Nanjing, China; 2 School of Basic Medical Sciences, Fujian Medical University, Fuzhou, China

**Keywords:** histone, histone modification, deubiquitination, homologous recombination, genomic instability, DNA damage response, DNA replication, DNA repair, yeast, yeast genetics

## Abstract

Canonical oncohistones are histone H3 mutations in the N-terminal tail associated with tumors and affect gene expression by altering H3 post-translational modifications (PTMs) and the epigenetic landscape. Noncanonical oncohistone mutations occur in both tails and globular domains of all four core histones and alter gene expression by perturbing chromatin remodeling. However, the effects and mechanisms of noncanonical oncohistones remain largely unknown. Here we characterized 16 noncanonical H2B oncohistones in the fission yeast *Schizosaccharomyces pombe*. We found that seven of them exhibited temperature sensitivities and 11 exhibited genotoxic sensitivities. A detailed study of two of these onco-mutants H2BG52D and H2BP102L revealed that they were defective in homologous recombination (HR) repair with compromised histone eviction and Rad51 recruitment. Interestingly, their genotoxic sensitivities and HR defects were rescued by the inactivation of the H2BK119 deubiquitination function of Ubp8 in the Spt-Ada-Gcn5-Acetyltransferase (SAGA) complex. The levels of H2BK119 monoubiquitination (H2Bub) in the H2BG52D and H2BP102L mutants are reduced in global genome and at local DNA break sites presumably due to enhanced recruitment of Ubp8 onto nucleosomes and are recovered upon loss of H2B deubiquitination function of the SAGA complex. Moreover, H2BG52D and H2BP102L heterozygotes exhibit genotoxic sensitivities and reduced H2Bub in *cis*. We therefore conclude that H2BG52D and H2BP102L oncohistones affect HR repair and genome stability *via* the reduction of H2Bub and propose that other noncanonical oncohistones may also affect histone PTMs to cause diseases.

Oncohistones refer to histone genes that are mutated in human cancers (1, 2). These missense mutations occur in one of many histone genes. H3.3K27M, H3.3G34R/V/W/L, and H3.3K36M are canonical oncohistones that are frequently found in pediatric glioma and sarcoma (3, 4, 5, 6, 7). Besides, genome sequence analyses of common cancers reveal many missense mutations at distinct sites in the histone H3, H4, H2A, and H2B genes, which are termed noncanonical oncohistones. They occur at lower frequencies in a variety of cancer types and collectively at a higher frequency than the canonical H3 oncohistone. Noncanonical onco-mutations occur in both the histone tails and globular domains, and at or near the sites of post-translational modifications (PTMs) (8, 9).

The mechanism of canonical H3 oncohistones causing cancer involves alterations of classical PTMs of H3 and thus leads to altered expression of cancer-related genes (10, 11, 12, 13). The H3.3K27M mutation leads to the global reduction of H3K27me3 levels by inhibiting the H3K27 methyltransferase EZH2 in the PRC2 complex in *trans* (14, 15) and by sequestering the PRC2 complex to poised enhancers (16, 17), and drives gene expression by SMARCA4-mediated chromatin remodeling (18). Likewise, the H3.3K36M mutation results in the global disruption of H3K36me3 *via* the inhibition of H3K36 methyltransferase SETD2 in *trans* (19, 20). In contrast, the H3.3G34R/V/W/L mutations reduce the abundance of H3.3K36me3 through the inhibition of SETD2 in *cis* (21, 22). H3.3 onco-mutations may not only alter gene expression but also cause genomic instability which is another hallmark of cancer (23, 24). The H3.3K27M and H3.3G34R/V mutants have altered interactions with DNA repair proteins and thus affect DNA repair in pediatric high-grade gliomas (25). Therefore, targeting DNA repair factors could improve their treatments (26, 27). Moreover, H3G34 onco-mutations in the *Schizosaccharomyces pombe* (*S. pombe*) can cause defects in homologous recombination (HR) and genomic stability (28, 29). Chondrocyte progenitor cells harboring H3.3K36M are also defective in HR repair of DNA damage (19), as Set2/SETD2 and H3K36me3 are critical for DNA repair (30).

In contrast to canonical H3 oncohistones, noncanonical histone mutations occur at both tails and globular domains. Although they have the potential to affect histone PTMs, the evidence is lacking. Therefore, they appear to influence gene expression by locally altering nucleosome stability, chromatin structure, and chromatin remodeling (1, 31, 32, 33). H2BE76K, for instance, is the most prevalent noncanonical oncohistone. It disrupts H2B-H4 interaction, destabilizes nucleosomes, and alters chromatin architecture to promote the expression of oncogenic genes like *ADAM19* (9, 34, 35). However, because of many diverse noncanonical oncohistones and their lack of association with specific cancer types, their functional consequences and oncogenic mechanisms remain largely unknown. In addition to nucleosome destabilization or chromatin corruption, whether noncanonical oncohistones affect histone PTMs and genome stability, is an additional question that needs to be addressed in the study of diverse cancers.

Genetic studies in model organisms have been used as alternative approaches to uncover phenotypes and effector genes of oncohistones. The functional consequences or genetic suppressors of H3K27M, H3G34R/V/W, and H3K36M mutations have been studied in budding (36) and fission yeast (28, 29), worm (37), and fly (38). Moreover, the cellular effects on growth and viability of noncanonical oncohistones have been systematically screened in the budding yeast *Saccharomyces cerevisiae* (*S. cerevisiae*) using the “humanized” histones library (32). However, extensive functional and mechanical studies of noncanonical oncohistones in model organisms are still lacking.

Because the *S. pombe* genome contains only one H2B gene compared with two paralogs in the *S. cerevisiae* genome and 18 canonical H2B paralogs in the human genome, we chose to investigate H2B onco-mutants in the *S. pombe* as representatives of noncanonical oncohistones. We constructed 16 *S. pombe* strains, each with a conserved and highly frequent H2B onco-mutation, and screened their phenotypes. Furthermore, we focused on two of these onco-mutants, H2BG52D and H2BP102L, and found that they were defective in homologous recombination of DNA repair due to reduced H2Bub in *cis*. H2Bub is evolutionarily conserved from yeast to humans, which is located at K119 in fission yeast, K123 in budding yeast, and K120 in humans. H2Bub plays a key role in transcription and is associated with the actively transcribed genome (39, 40). H2Bub is also involved in DNA replication (41), replication stress response (42, 43, 44), and DNA damage repair (45, 46, 47, 48). The reduction of H2Bub in the H2BG52D and H2BP102L mutants was restored by mutating the deubiquitination module components, Ubp8 and Sgf11, in the SAGA complex. This largely suppressed the genomic instability of the H2BG52D and H2BP102L mutants. Similar observations were also made for the H2BD67N mutant, and the heterozygous H2BG52D and H2BP102L mutants. We therefore uncovered a novel H2Bub-dependent mechanism underlying the genomic instability caused by H2B oncohistones.

## Results

### The phenotypes of 16 H2B onco-mutants in *S. pombe*

**Figure 1:**
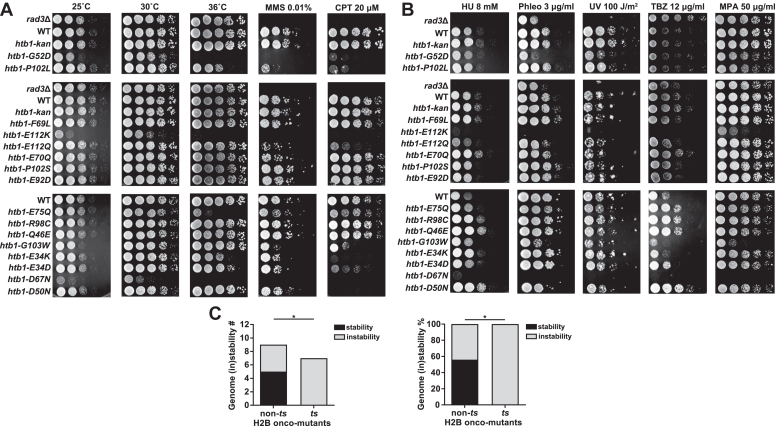
**The phenotypes of 16 *S. pombe* H2B onco-mutants.***A* and *B*, the growth of WT (TK8), *htb1-G52D* (YGF277), *htb1-P102L* (YGF279), *htb1-F69L* (YGF323), *htb1-E112K* (YGF328), *htb1-E112Q* (YGF321), *htb1-E70Q* (YGF330), *htb1-P102S* (YGF325), *htb1-E92D* (YGF326), *htb1-E75Q* (YGF317), *htb1-R98C* (YGF322), *htb1-Q46E* (YGF331), *htb1-G103W* (YGF318), *htb1-E34K* (YGF275), *htb1-E34D* (YGF329), *htb1-D67N* (YGF324), and *htb1-D50N* (YGF332) strains under indicated conditions. The *rad3*Δ (LD297) strain is the positive control, and the *htb1-kan* (YGF1) strain is the isogenic control in which the *htb1*^*+*^ gene with the *kanMX6* marker insertion downstream of its 3′ UTR. *C*, the number (*Left panel*) and percentage (*Right panel*) of *ts* and non-*ts* H2B onco-mutants with and without genome instability are shown. The significant correlation between *ts* and genome instability in H2B onco-mutants is subjected to Fisher’s exact test.

To dissect the role of H2B oncohistones, we constructed 16 strains of *S. pombe*, each carrying an onco-mutation in the H2B gene *htb1*^+^. These H2B onco-mutations occur at the conserved sites, and most of them have been reported among the top 20 of H2B mutations by frequency in human cancers (8, 9). These strains were spotted onto plates to investigate their growth under the indicated conditions: distinct temperature (25 °C, 30 °C, and 36 °C), replication stress (MMS, CPT, and HU), DNA damage (Phleomycin and UV irradiation), chromosome missegregation (TBZ), and inhibition of transcription elongation (MPA). MMS, CPT, and HU cause forks to stall or collapse during DNA replication. Phleomycin and UV directly lead to DNA damage or breaks. TBZ destabilizes the microtubule and leads to chromosome missegregation. We observed slower growth of *htb1-G52D*, *htb1-E112K*, and *htb1-D67N* cells compared with wild-type (WT) *htb1*^+^ under permissive temperature (Fig. 1*A*), which was consistent with the result from budding yeast with the “humanized” oncohistones (32). Intriguingly, seven out of the 16 mutants: *htb1-G52D*, *htb1-P102L, htb1-E112K*, *htb1-E75Q*, *htb1-E34K*, *htb1-E34D*, and *htb1-D67N* were temperature-sensitive (*ts*) in varying extents. Unexpectedly, 11 out of the 16 mutants comprising the *htb1-G52D*, *htb1-P102L*, *htb1-E112K*, *htb1-E112Q*, *htb1-E70Q*, *htb1-E75Q*, *htb1-G103W*, *htb1-E34K*, *htb1-E34D*, *htb1-D67N*, and *htb1-D50N* were sensitive to at least one genotoxic agent of replication stress and DNA damage (Fig. 1, *A* and *B*), suggesting that these 11 H2B onco-mutants potentially exhibit genomic instability. The above results were reproducible when using an independent alternative transformant from each strain (Fig. S1, *A* and *B*). The phenotypes of 16 H2B onco-mutants in *S. pombe* are summarized in Table 1. Interestingly, all seven *ts* onco-mutants showed genomic instability, and five out of nine non-*ts* onco-mutants failed to show genomic instability. The correlation between *ts* and genomic instability was statistically significant (Fig. 1*C*), suggesting that the normal function or stability of H2B in the nucleosome is necessary for genome stability. In addition, *htb1-P102L/S*, *htb1-E112K/Q*, and *htb1-E34K/D* which have two distinct mutations in one site exhibit diverse phenotypes. The *htb1-P102L*, but not *htb1-P102S* mutant, showed defects in *ts* and genomic instability. The *htb1-E112K* and *htb1-E34K* mutations exhibited more severe phenotypes than that of *htb1-E112Q* and *htb1-E34D* mutations, respectively (Fig. 1, *A* and *B*). These results correlate with previous findings of diverse phenotypes of H2BE76K/Q (9) and H3G34R/V/W (29), thus providing validation for our approach.

**Table 1:** Summary of phenotypes of 16 H2B onco-mutants in *S. pombe*

H2B onco-mutants	Growth	Genotoxic response	Transcription
G52D	+ (*ts*)	+	+
P102L	(*ts*)	+	+
F69L			
E112K	+ (*ts*)	+	
E112Q		+	
E70Q		+	
P102S			
E92D			
E75Q	(*ts*)	+	
R98C			
Q46E			
G103W		+	+
E34K	(*ts*)	+	
E34D	(*ts*)	+	
D67N	+ (*ts*)	+	+
D50N		+	

+, defective phenotype; *ts*, temperature sensitive.

Next, we performed RNA-seq analyses on six selected H2B onco-mutants to determine whether their genomic instability might be due to changes in gene expression. Although most H2B onco-mutants were not sensitive to MPA, we found that the expression of many genes was increased (Fig. S1*C*). Classification analyses (DEGs, KEGG, and GO) revealed that the autophagy and nutrient metabolism pathways were significantly enriched (Fig. S1, *D–F*). Furthermore, we found that the expression of genes annotated as DNA replication, repair, and recombination in GO-BP and KEGG were not significantly altered (Fig. S1*G*). These results suggest that the genome instability in H2B onco-mutants is not largely due to dysregulation of transcription, although we cannot rigorously exclude this possibility.

### The DNA synthesis defect during damaged fork stalling and restart in the *htb1-G52D* and *htb1-P102L* mutants

To gain insights into the mechanisms contributing to genomic instability in H2B onco-mutants, we focused on studies of the *htb1-G52D* and *htb1-P102L* mutants. Their equivalent mutation sites in human cancers are H2BG53 and H2BP103, respectively, and their mutation frequencies are ranked second and 11th among all H2B mutation sites, respectively (9). H2BG53D and H2BP103L are the main mutation types that have not been intensely studied but exhibit strong phenotypes in our genetic screen.

To independently test whether the *htb1-G52D* and *htb1-P102L* cells were sensitive to strand breaks by DNA damage directly, these mutants were subjected to ionizing radiation (IR). We found that the *htb1-G52D* mutant was sensitive to IR whereas the *htb1-P102L* mutant was less sensitive (Fig. S2*A*), which resembled their response to genotoxic drugs. Because their growth was temperature-sensitive, we tested whether temperature affects the DNA damage response of the *htb1-G52D* and *htb1-P102L* mutants by performing the MMS spot assays at permissive temperature (25 °C) and semi-restrictive temperature (33 °C) (Fig. S2*B*). Both *htb1-G52D* and *htb1-P102L* mutants were still sensitive to MMS at 25 °C and 33 °C, indicating that their *ts* is not necessary for their MMS sensitivities. In addition to the chronic exposure to MMS, we studied the survival rate of the *htb1-G52D* and *htb1-P102L* mutants under the acute treatment of MMS. Upon treatment with 0.05% MMS for 2 h and 4 h in liquid culture, the survival rate of the *htb1-G52D* and *htb1-P102L* mutants was reduced (Fig. S2*C*). Collectively, we conclude that the *htb1-G52D* and *htb1-P102L* mutants are defective in response to both replication stress and DNA damage.

**Figure 2:**
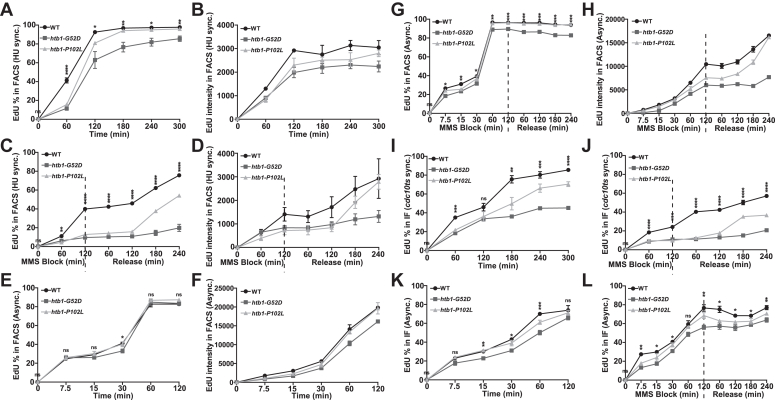
**Measurement of DNA synthesis using EdU incorporation in the *htb1-G52D* and *htb1-P102L* mutants under normal and MMS block/release conditions.***A*–*D*, EdU-incorporating strains of WT (J2172), *htb1-G52D* (YGF281), and *htb1-P102L* (YGF282) were synchronized into early S phase by 12 mM HU treatment, and then labeled with EdU at the indicated time points under normal condition (*A* and *B*) and 0.05% MMS block and release condition (*C* and *D*). *E*–*H*, the asynchronized cells were labeled with EdU at the indicated time points under normal conditions (*E* and *F*) and 0.05% MMS block and release condition (*G* and *H*). The percentages of cells with positive EdU signals (*A*, *C*, *E*, and *G*) and the mean intensities of EdU signals (*B*, *D*, *F*, and *H*) were analyzed with FACS. *I* and *J*, the *cdc10* mutant cells were synchronized into the G1 phase by incubating at 36 °C for 6 h and then released into the S phase by incubating at 25 °C for 1 h. The S phase cells were labeled with EdU at the indicated time points under normal condition (*I*) and 0.05% MMS block and release condition (*J*). *K* and *L*, the asynchronized cells were labeled with EdU at the indicated time points under normal condition (*K*) and 0.05% MMS block and release condition (*L*). The percentages of cells with positive EdU signals were observed with immunofluorescence (IF) (*I*–*L*). MMS block is shown on the *left* of the *dotted line*, and then release into normal condition is shown on the right. Three independent biological repeats are averaged and error bars represent SEMs. One-way ANOVA is statistically performed at each time point for the percentages of EdU-positive cells.

Next, we used FACS to measure the amount of newly synthesized DNA as results of DNA replication under normal growth conditions and the DNA repair process under acute MMS block and release. The *htb1-G52D* and *htb1-P102L* mutants were engineered to be capable of incorporating EdU, and these strains were verified for their MMS sensitivities (Fig. S2, *D* and *E*). We first used HU to synchronize cells into the early S phase and then labeled cells with EdU in the presence and absence of MMS treatment. During EdU labeling in physiological conditions without MMS, the *htb1-G52D* and *htb1-P102L* mutants exhibited approximately 20% and 10% reduction of both number of cells with EdU incorporation and intensity of EdU signals compared with WT, respectively (Fig. 2, *A* and *B*). This implies that the *htb1-G52D* mutation affects DNA replication in the S phase, probably because of more replication stress or genomic instability. Under the MMS treatment and release conditions, the *htb1-G52D* and *htb1-P102L* mutants exhibited approximately 70% reduced number of cells with EdU incorporation and about 50% reduced intensity of EdU signals during both MMS block and release (Fig. 2, *C* and *D*). These defects are not caused by delay of the cell growth in the fork stalling and restart during MMS block and release because the *htb1-G52D* and *htb1-P102L* cells exhibited no significant difference in growth rate compared with the WT during MMS block and release (Fig. S2, *F* and *G*). To avoid potential complications from HU synchronization, we also performed similar experiments without HU treatment and synchronization. Consistently, the *htb1-G52D* cells displayed fewer EdU incorporation and lower EdU intensity than the WT in the physiological condition (Fig. 2, *E* and *F*). Under MMS block and release conditions, the *htb1-G52D* and *htb1-P102L* mutations also reduced EdU signals under MMS and delayed EdU synthesis after release (Fig. 2, *G* and *H*).

In addition to FACS analysis, we observed EdU-positive cells by immunofluorescence (IF). Because HU causes replication stress which may complicate analysis, the *cdc10ts* cells were synchronized at G1 and released into the S phase. The result of IF demonstrated that the *htb1-G52D* and *htb1-P102L* mutants in the S phase exhibited approximately 40% and 20% reduction of cell percentage with EdU compared with WT, respectively (Fig. 2*I*). Under the MMS block and release conditions, the *htb1-G52D* and *htb1-P102L* mutants in S phase exhibited approximately 60% reduced number of cells with EdU during the MMS block and delayed about 70% rate of EdU re-synthesis after MMS release (Fig. 2*J*). For the asynchronized cells, we observed similar results but with smaller differences between *htb1-G52D/P102L* and WT cells with and without MMS (Fig. 2, *K* and *L*). These IF results were consistent with the previous FACS results. Together, the data suggest that replication stress results in defective fork stalling and delayed fork repair or recovery in the *htb1-G52D* and *htb1-P102L* mutants.

### Genetic interaction analysis indicates defective Rad51-mediated HR repair in the *htb1-G52D* and *htb1-P102L* mutants

We then performed the genetic interaction analyses to identify the potential defective pathways in the *htb1-G52D* and *htb1-P102L* mutants in response to replication and DNA damage by constructing double mutants with the mutations of the genes involved in DNA damage checkpoint, repair, and tolerance.

**Figure 3:**
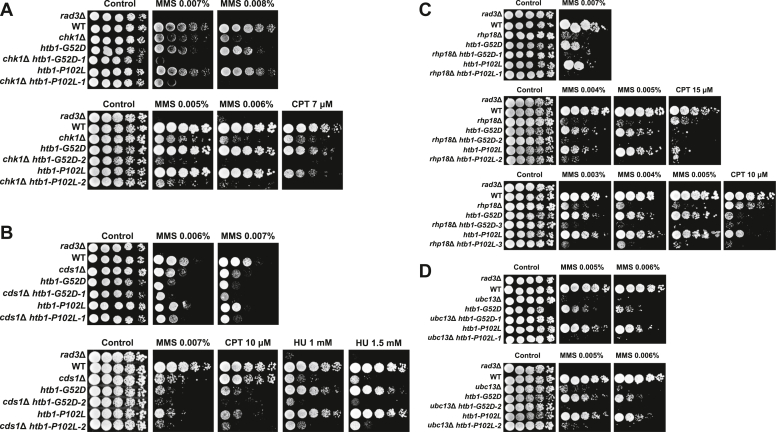

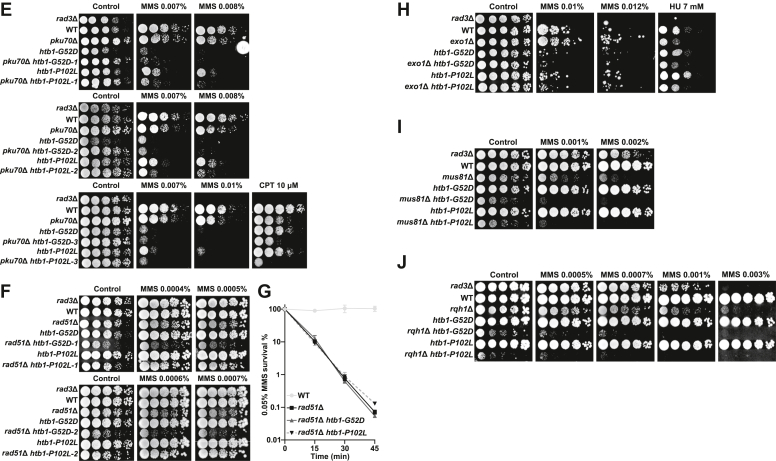
**Genetic interactions between *htb1-G52D/P102L* and indicated mutants.***A*, MMS and CPT sensitivities of *chk1*Δ (Ets12), *chk1*Δ *htb1-G52D* (YGF291), and *chk1*Δ *htb1-P102L* (YGF292) mutants. *B*, MMS, CPT, and HU sensitivities of *cds1*Δ (Ets13), *cds1*Δ *htb1-G52D* (YGF287), and *cds1*Δ *htb1-P102L* (YGF288) mutants. *C*, MMS and CPT sensitivities of *rhp18*Δ (FY20976), *rhp18*Δ *htb1-G52D* (YGF299), and *rhp18*Δ *htb1-P102L* (YGF300) mutants. *D*, MMS sensitivities of *ubc13*Δ (FY20970), *ubc13*Δ *htb1-G52D* (YGF295), and *ubc13*Δ *htb1-P102L* (YGF296) mutants. *E*, MMS and CPT sensitivities of *pku70*Δ (YGF266), *pku70*Δ *htb1-G52D* (YGF293), and *pku70*Δ *htb1-P102L* (YGF294) mutants. *F*, MMS sensitivities of *rad51*Δ (FY18537), *rad51*Δ *htb1-G52D* (YGF301), and *rad51*Δ *htb1-P102L* (YGF302) mutants. *G*, the viability of *rad51*Δ (FY18537), *rad51*Δ *htb1-G52D* (YGF301), and *rad51*Δ *htb1-P102L* (YGF302) under 0.05% MMS treatment. Three independent biological repeats are averaged and SD is shown as the error bar. *H*, MMS and HU sensitivities of *exo1*Δ (YGF357), *exo1*Δ *htb1-G52D* (YGF358), and *exo1*Δ *htb1-P102L* (YGF359) mutants. *I*, MMS sensitivities of *mus81*Δ (YGF396), *mus81*Δ *htb1-G52D* (YGF397), and *mus81*Δ *htb1-P102L* (YGF398) mutants. *J*, MMS sensitivities of *rqh1*Δ (YGF393), *rqh1*Δ *htb1-G52D* (YGF394), and *rqh1*Δ *htb1-P102L* (YGF395) mutants. Two or three repeated experiments with independent biological transformants of double mutants were shown in the indicated panels.

Following replication stress and DNA damage, checkpoint signaling is first activated to arrest cell cycle progression. The upstream sensor kinases Rad3 (ATR) and Tel1 (ATM) phosphorylate H2AS128/S129, which is also termed γH2A, and consequently activate the downstream effector kinases Chk1 (CHK1) and Cds1 (CHK2). We found that *chk1*Δ and *cds1*Δ, which are the null mutants of *chk1*^+^ and *cds1*^+^, together with *htb1-G52D* and *htb1-P102L* showed synergistic MMS and CPT or HU sensitivities compared with either single mutant (Fig. 3, *A* and *B*), suggesting that the *htb1-G52D* and *htb1-P102L* mutations were independent of the intact checkpoint functions in response to MMS. To independently test this speculation, we calculated the septation indices as indications of cell cycle phases of *htb1-G52D*, *htb1-P102L*, and their double mutants with *chk1*Δ and *cds1*Δ under MMS block and release. We found that the septation indices in the *htb1-G52D* and *htb1-P102L*, but not *rad3*Δ mutants, were reduced in response to MMS, implying that their checkpoint kinases especially for Rad3 was efficiently activated (Fig. S3*A*). Besides, the arrested cell cycle of the double mutants *cds1*Δ *htb1-G52D* and *cds1*Δ *htb1-P102L* indicated that Chk1 was activated by MMS and not affected by *htb1-G52D* and *htb1-P102L* mutations (Fig. S3*B*). The septation indices of *chk1*Δ *htb1-G52D* and *chk1*Δ *htb1-P102L* after MMS treatment were unchanged (Fig. S3*B*). Since Chk1 appears to be the dominant effector kinase in response to MMS, we cannot conclude that Cds1 is not affected. Accordingly, we conducted the assay of phosphorylation band shift. After the validation of MMS sensitivities in the *htb1-G52D* and *htb1-P102L* strains with either Chk1-HA and Cds1-HA (Fig. S3*C*), the immunoblots demonstrated that Chk1 and Cds1 were normally phosphorylated after MMS block and release, further supporting that checkpoint activation and maintenance remain functional in the *htb1-G52D* and *htb1-P102L* mutants (Fig. S3, *D* and *E*).

In addition to checkpoint, DNA damage tolerance like post-replication repair (PRR) and DNA damage repair such as non-homologous end join (NHEJ) and homologous recombination (HR), are also involved in MMS damage response (49). The null mutations of PRR genes, which are *rhp18*Δ (involved in translesion synthesis) (Fig. 3*C*) and *ubc13*Δ (involved in template switching) (Fig. 3*D*), might show mildly synergistic or additive sensitivities in response to MMS or CPT when combined with the *htb1-G52D/P102L* mutations. In addition to colony formation of *rhp18*Δ related mutants in MMS, we also observed that the cell cycle progression of two double mutants, *rhp18*Δ *htb1-G52D* and *rhp18*Δ *htb1-P102L*, which was indicated by their septation indices, was delayed compared with either single mutant after MMS release (Fig. S3, *A* and *F*). The results suggest that the *htb1-G52D* and *htb1-P102L* mutants influence both *rhp18/ubc13*-dependent PRR and -independent HR pathways in response to MMS-induced replication stress. Moreover, the deletion of NHEJ gene *pku70*^+^ with the *htb1-G52D* and *htb1-P102L* mutants also exhibited synergistic defects under MMS and CPT (Fig. 3*E*). In contrast, the null mutations of the HR genes *rad51*^+^ recombinase (Fig. 3, *F* and *G*) and *exo1*^*+*^ exonuclease (Fig. 3*H*) failed to show synergistic MMS or HU sensitivities with the *htb1-G52D* and *htb1-P102L* mutations. Taken together, these data suggest that the *htb1-G52D* and *htb1-P102L* mutants mainly affect the Rad51-dependent HR under MMS.

HR can be further divided into several sub-pathways, *i*.*e*., replication fork repair/restart by HR, also called break-induced replication (BIR), classical DSB repair (DSBR), and Rad51-independent single-strand annealing (SSA) (50). In addition to Rad51-mediated DSB repair, replication fork repair or restart by HR could be another pathway in the *htb1-G52D* and *htb1-P102L* cells in response to MMS. We found that the *htb1-G52D* and *htb1-P102L* mutations negatively interacted with the deletion of the Holliday junction resolvase *mus81*^*+*^ and RecQ helicase *rqh1*^*+*^, which are important for replication fork repair *via* HR under MMS (Fig. 3, *I* and *J*). This indicates that Rad51-dependent HR, but not Mus81-dependent HR and Rqh1-dependent HR, is the key defective pathway in the *htb1-G52D* and *htb1-P102L* cells under MMS.

### The *htb1-G52D* and *htb1-P102L* mutants are defective in HR repair

**Figure 4:**
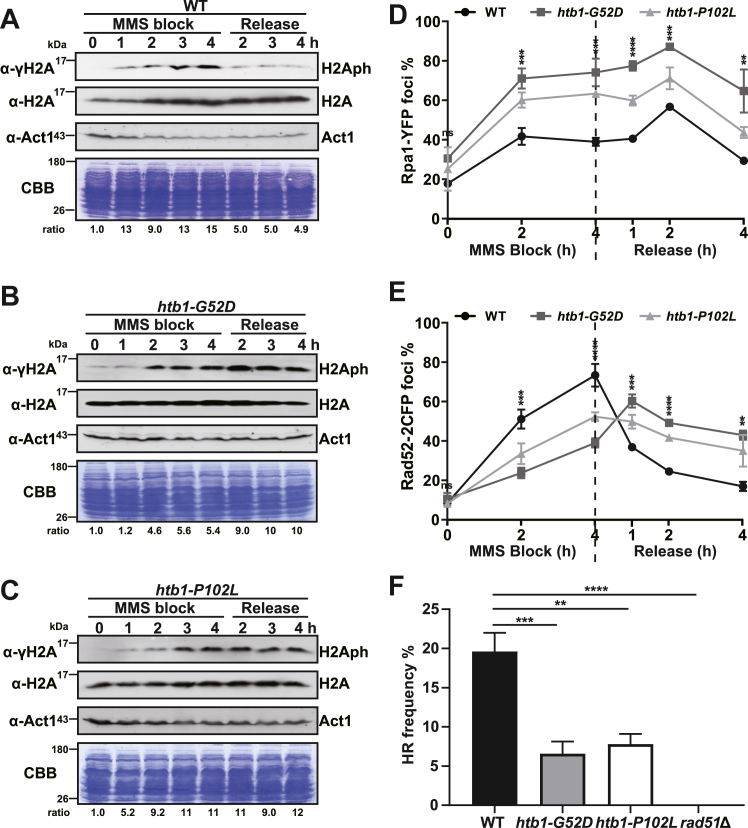
**Determination of levels of γH2A, RPA, and Rad52 markers during MMS block and release as well as HR frequency in the *htb1-G52D* and *htb1-P102L* mutants.***A*–*C*, the immunoblots of γH2A and H2A abundance during 0.05% MMS block and release at the indicated time points in WT (TK8) (*A*), *htb1-G52D* (YGF277) (*B*) and *htb1-P102L* (YGF279) (*C*) strains. The γH2A signal normalized to H2A signal is used to calculate the ratio of the indicated time points to 0 h. *D* and *E*, the percentage of Rpa1-YFP foci (*D*) and Rad52-2CFP foci (*E*) in WT (DY2407), *htb1-G52D* (YGF276), and *htb1-P102L* (YGF278) cells under 0.05% MMS block and release at the indicated time points. The percentage of foci is averaged from three independent biological repeats. Error bars represent SDs. One-way ANOVA is statistically performed at each time point. *F*, determination of HR frequencies in WT (TK8), *htb1-G52D* (YGF277), *htb1-P102L* (YGF279), and *rad51*Δ (FY14135) strains. HR efficiency is calculated as the percentage of the number of Leu^+^ colonies relative to the number of Ura^+^ colonies. Results are averaged from three independent biological repeats. Error bars represent SEMs. One-way ANOVA is statistically performed for multiple comparisons between the indicated samples and WT.

To independently test the findings of the genetic interaction analyses, we monitored the levels of γH2A during MMS block and release, which is an important marker of replication stress and DNA damage (51, 52). Immunoblots demonstrated that the levels of γH2A in the WT cells were increased after MMS treatment and decreased after MMS release (Figs. 4*A* and S4*A*). In contrast, γH2A levels in the *htb1-G52D* (Figs. 4*B* and S4*B*) and *htb1-P102L* cells (Figs. 4*C* and S4*C*) were induced after MMS treatment, but failed to decrease, and remained at the similar induced levels after MMS release, suggesting that the *htb1-G52D* and *htb1-P102L* mutations delayed the DNA repair.

In addition to the γH2A levels, we also used the fluorescent microscope to observe the numbers of Rpa1 and Rad52 foci in the *htb1-G52D* and *htb1-P102L* cells during MMS block and release. Rpa1-YFP and Rad52-2CFP contained in the *htb1-G52D* and *htb1-P102L* mutants were validated for MMS sensitivity (Fig. S4*D*). We then observed that the percentage of Rpa1 foci in the *htb1-G52D* and *htb1-P102L* cells was higher than that in WT cells throughout MMS treatment and release (Fig. 4*D*), suggesting that the *htb1-G52D* and *htb1-P102L* cells contain more RPA-coated ssDNA that might be due to inefficient repair under spontaneous and induced damage conditions. Therefore, normal replicating genomes of the *htb1-G52D* and *htb1-P102L* cells might be unstable and become more severe after fork damage. In addition to Rpa1 foci, the percentage of Rad52 foci in the *htb1-G52D* and *htb1-P102L* cells was slowly increased compared with that in WT cells under MMS treatment, suggesting that the function of Rad52 in Rad51 recruitment was compromised during HR repair. After MMS release, the percentage of Rad52 foci in the *htb1-G52D* and *htb1-P102L* cells remained at higher levels compared with that of WT cells (Fig. 4*E*). The change in the percentage of Rpa1 and Rad52 foci was not due to the change in their abundance of total protein (Fig. S4*E*). Together, these data imply that Rad52-dependent HR repair is compromised in the *htb1-G52D* and *htb1-P102L* cells.

To further investigate these implications, we directly measured the frequency of HR repair. We transformed the *leu1-32 ura4-D18* cells with the *leu1* fragments which generate Leu^+^ by HR repair, and with the *ura4*^+^ plasmids which are used for the normalization of transformation efficiency. The frequency of HR was calculated as the number of Leu^+^ transformants divided by that of Ura^+^ transformants. We found that the HR frequency of the positive control *rad51*Δ was very low as expected, and the HR frequency was also reduced in the *htb1-G52D* and *htb1-P102L* mutants (Fig. 4*F*). HR operates in S and G2 phases of the cell cycle in *S. pombe*. The reduced HR frequency appears not to be caused by the shortening of the S phase since the *htb1-G52D* cells replicate slowly with a longer S phase and the *htb1-P102L* cells replicate with a normal S phase.

### The *htb1-G52D* and *htb1-P102L* mutants show reduced Rad51 loading onto the HO-induced DSB site

**Figure 5:**
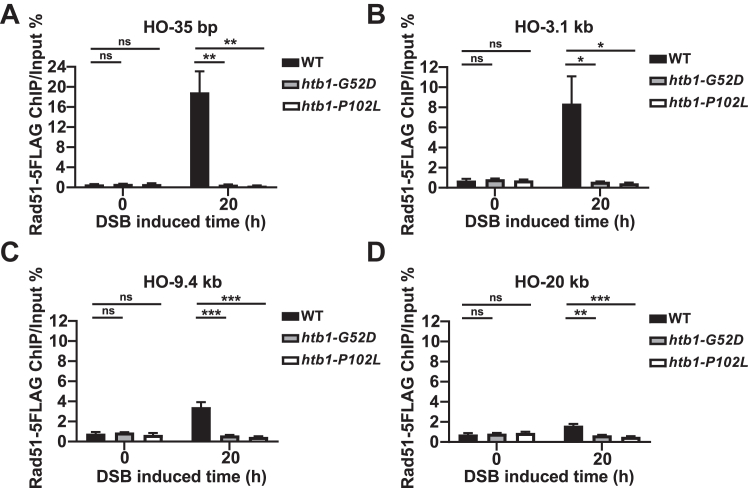
**The recruitment of Rad51aroundthe HO-induced DSB site in the *htb1-G52D* and *P102L* mutant.***A*–*D*, the ChIP-qPCR analysis of Rad51-5FLAG enrichment in WT (YGF466), *htb1-G52D* (YGF462), and *htb1-P102L* (YGF463) at 35 bp (*A*), 3.1 kb (*B*), 9.4 kb (*C*), and 20 kb (*D*) adjacent to the HO-induced DSB site after HO-induced 0 h and 20 h. The fold change is shown as the normalization of the enrichment in the indicated mutants to the WT at HO-induced 0 h and 20 h, separately. The data from three independent biological repeats are averaged. Error bars represent SEMs. One-way ANOVA is used for multiple comparisons between the indicated samples and WT at HO-induced 0 h and 20 h.

The above results motivated us to study the mechanisms of HR defects in the *htb1-G52D* and *htb1-P102L* mutants. We employed the established genetic system of P*nmt41*-HO endonuclease-induced HO cut to introduce one specific DSB site in *S. pombe* genome (53, 54, 55). We then performed ChIP experiments to investigate whether the recruitment of Rad51 to the HO-induced DSB site was compromised during HR in the *htb1-G52D* and *htb1-P102L* strains. A similar percentage (∼90%) of DSB formation was found in all the tested strains after inducing HO overexpression for 20 h (Fig. S5*A*). Subsequently, we engineered and confirmed these strains in which Rad51 was tagged with 5FLAG (Fig. S5*B*). We then validated the ChIP assay and found that Rad51-5FLAG was enriched around HO-induced DSB sites compared with the untagged strain (Fig. S5, *C* and *D*). After HO induction for 20 h instead of 0 h, the Rad51-5FLAG enrichment at 35 bp, 3.1 kb, 9.4 kb, and 20 kb away from the DSB site, was reduced in the *htb1-G52D* and *htb1-P102L* cells compared with the WT (Fig. 5, *A–D*). The Rad51-5FLAG enrichment and its reduction around DSB sites in the *htb1-G52D* and *htb1-P102L* cells failed to occur at *act1*^+^ locus, which has no induced DSB (Fig. S5*E*). This suggests that compromised Rad51 enrichment at DNA break ends is responsible for the HR defects of the *htb1-G52D* and *htb1-P102L* mutants.

### The *htb1-G52D* and *htb1-P102L* mutants show histone retention around the local HO-induced DSB site and exhibit the relaxed global chromatin

**Figure 6:**
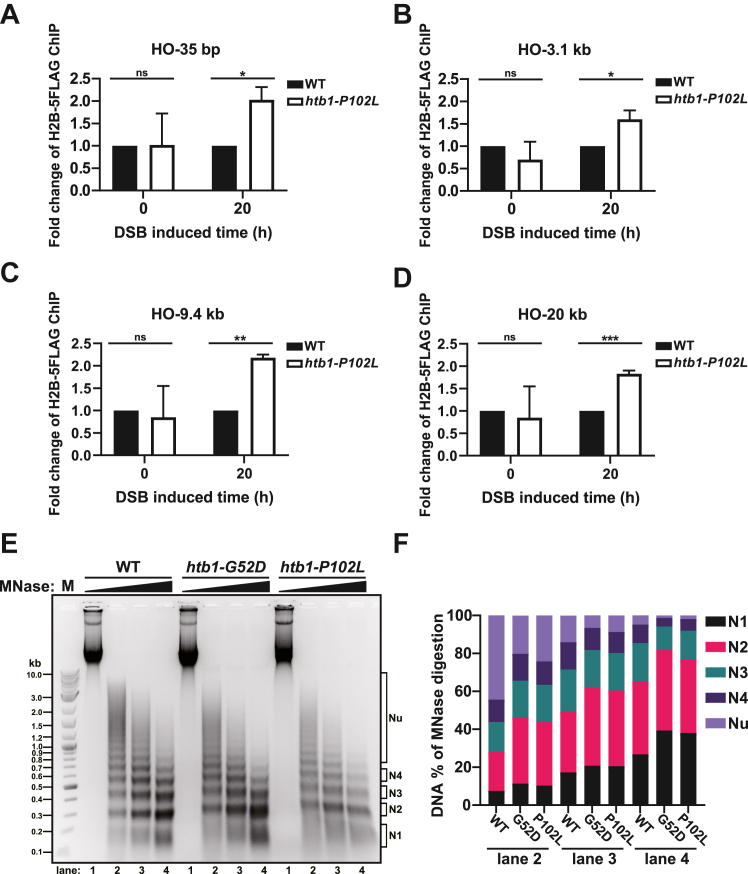
**The association of nucleosomes near the HO-induced DSB site in the *htb1-G52D* and *P102L* mutant.***A*–*D*, the ChIP-qPCR analysis of H2B-5FLAG enrichment at 35 bp (*A*), 3.1 kb (*B*), 9.4 kb (*C*), and 20 kb (*D*) adjacent to the HO-induced DSB site after HO-induced 0 h or 20 h. The fold change of H2B-5FLAG enrichment in the *htb1-P102L* (YGF492) relative to WT (DY49) is shown at HO-induced 0 h and 20 h, separately. The data from two or three independent biological repeats are averaged. Error bars represent SEMs. Student’s *t* test is used for comparing *htb1-P102L* with WT at HO-induced 0 h or 20 h. *E*, the DNA gel after MNase digestion of chromatin from WT (TK8), *htb1-G52D* (YGF277), and *htb1-P102L* (YGF279) strains is shown. N indicates the nucleosome DNA band; Nu indicates nucleosome DNA bands in the upper gel. *F*, quantification of nucleosomal DNA bands in lanes 2 to 4.

To study whether the decreased enrichment of Rad51 at the HO-induced DSB site is due to a change in histone or nucleosome occupancy during HR in the *htb1-G52D* and *htb1-P102L* mutants, we first verified that these mutations failed to affect the total abundance of H2B and H3 in the presence and absence of MMS treatment (Fig. S6, *A* and *B*). We then performed the ChIP experiments to measure the H2B enrichment representing local nucleosome occupancy near the HO-induced DSB site. Initially, we used a commercial polyclonal antibody of H2B for the ChIP assay. The amount of H2B enrichment in the *htb1-G52D* cells relative to the WT was slightly increased at 35 bp, 9.4 kb, and significantly increased at 3.1 kb, 20 kb away from the HO-induced DSB site compared with the un-induced condition (Fig. S6, *C*–*F*), but was not altered at *act1*^+^ gene (Fig. S6*G*). The amount of H2B enrichment in the *htb1-P102L* cells relative to the WT appeared to be increased but displayed large variations. To avoid non-specific binding and variations using the polyclonal antibody of H2B for ChIP, we successfully tagged H2B at the C-terminal with 5FLAG in the *htb1*^+^ and *htb1-P102L* strains but failed in the *htb1-G52D* strain, probably due to the synthetic lethality of *htb1-G52D-5FLAG.* The H2B-5FLAG ChIP experiments demonstrated that the amount of H2B-5FLAG enrichment in the *htb1-P102L* cells relative to the WT was significantly increased at 35 bp, 3.1 kb, 9.4 kb, and 20 kb away from the HO-induced DSB site compared with the un-induced condition (Fig. 6, *A*–*D*), but was not changed at *act1*^+^ locus (Fig. S6*H*). According to the previous result which shows that reduced Rad51 loading in H2Bub deficient cells is due to decreased histone eviction during end resection in budding yeast (56), we propose that reduced histone eviction or histone retention caused by the *htb1-G52D* and *htb1-P102L* mutations during end resection is also responsible for their HR defects.

In addition to the local nucleosome occupancy, we also investigated whether global nucleosome occupancy or general chromatin architecture was affected by the *htb1-G52D* and *htb1-P102L* mutations. The *htb1-G52D* mutation occurs at the DNA binding loop L1 in the H2B fold domain, and changing from a neutral to a negative charge may attenuate the H2B-DNA interaction. The *htb1-P102L* mutation is located in the junction between α-helix α3 and αC near the H2B fold domain, and changing to an aliphatic hydrophobic side chain may interfere with H2B-H2A interaction. Consistently, MNase digestion assay revealed that the chromatin in the *htb1-G52D* and *htb1-P102L* mutants was digested more readily than that of WT, suggesting that their unstable nucleosomes make global chromatin structure more relaxed (Fig. 6, *E* and *F*). Results from another independent biological repeat were consistently shown (Fig. S6, *I* and *J*). These data also agree with the previous study demonstrating that H2BG53D relaxes global chromatin in human cells (57), and explain our finding that the expression of most genes is upregulated in the *htb1-G52D* and *htb1-P102L* mutants. Taken together, we conclude that the *htb1-G52D* and *htb1-P102L* mutations affect both local nucleosome occupancy around DNA break sites and global chromatin structure.

### The defect in H2B deubiquitination suppresses the genomic instability of the *htb1-G52D* and *htb1-P102L* mutants

**Figure 7:**
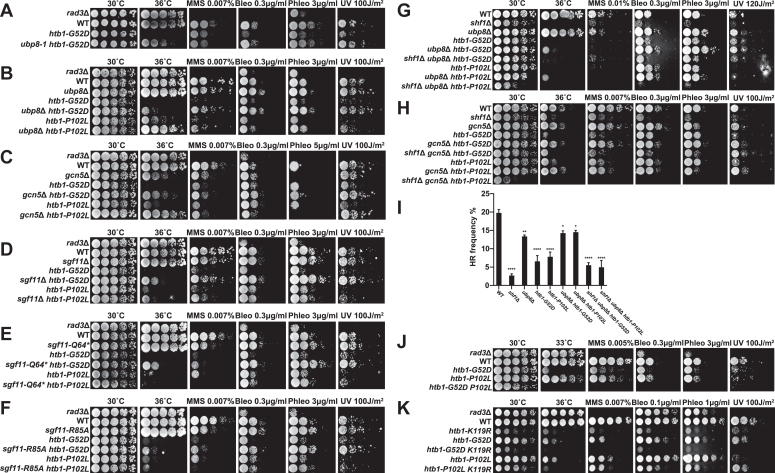
**Suppression of the genomic instability of the *htb1-G52D* and *htb1-P102L* mutants by the reduction of H2B deubiquitination.***A*, the growth of *htb1-G52D* (FY17274) cells and *ubp8-1 htb1-G52D* (YGF408) cells in the presence of 36 °C, MMS, bleomycin, phleomycin, and UV. *B*, the growth of *ubp8*Δ (YGF415), *htb1-G52D* (YGF277), *ubp8*Δ *htb1-G52D* (YGF416), *htb1-P102L* (YGF279), and *ubp8*Δ *htb1-P102L* (YGF417) cells in the presence of 36 °C, MMS, bleomycin, phleomycin, and UV. *C*, the growth of *gcn5*Δ (BN1), *htb1-G52D* (YGF277), *gcn5*Δ *htb1-G52D* (YGF418), *htb1-P102L* (YGF279), and *gcn5*Δ *htb1-P102L* (YGF419) cells in the presence of 36 °C, MMS, bleomycin, phleomycin, and UV. *D*, the growth of *sgf11*Δ (YGF482), *htb1-G52D* (YGF277), *sgf11*Δ *htb1-G52D* (YGF483), *htb1-P102L* (YGF279), and *sgf11*Δ *htb1-P102L* (YGF484) cells in the presence of 36 °C, MMS, bleomycin, phleomycin, and UV. *E*, the growth of *sgf11*-*Q64∗* (YGF485), *htb1-G52D* (YGF277), *sgf11*-*Q64∗ htb1-G52D* (YGF486), *htb1-P102L* (YGF279), and *sgf11*-*Q64∗ htb1-P102L* (YGF487) cells in the presence of 36 °C, MMS, bleomycin, phleomycin, and UV. *∗* indicates the stop codon. *F*, the growth of *sgf11*-*R85A* (YGF488), *htb1-G52D* (YGF277), *sgf11*-*R85A htb1-G52D* (YGF489), *htb1-P102L* (YGF279), and *sgf11*-*R85A htb1-P102L* (YGF490) cells in the presence of 36 °C, MMS, bleomycin, phleomycin, and UV. *G*, the growth of *shf1*Δ (YGF431), *ubp8*Δ (YGF415), *htb1-G52D* (YGF277), *ubp8*Δ *htb1-G52D* (YGF416), *shf1*Δ *ubp8*Δ *htb1-G52D* (YGF432), *htb1-P102L* (YGF279), *ubp8*Δ *htb1-P102L* (YGF417), and *shf1*Δ *ubp8*Δ *htb1-P102L* (YGF433) cells in the presence of 36 °C, MMS, bleomycin, phleomycin, and UV. *H*, the growth of *shf1*Δ (YGF431), *gcn5*Δ (BN1), *htb1-G52D* (YGF277), *gcn5*Δ *htb1-G52D* (YGF418), *shf1*Δ *gcn5*Δ *htb1-G52D* (YGF457), *htb1-P102L* (YGF279), *gcn5*Δ *htb1-P102L* (YGF419), and *shf1*Δ *gcn5*Δ *htb1-P102L* (YGF458) cells in the presence of 36 °C, MMS, bleomycin, phleomycin, and UV. *I*, the HR frequencies of *shf1*Δ (YGF431), *ubp8*Δ (YGF415), *htb1-G52D* (YGF277), *htb1-P102L* (YGF279), *ubp8*Δ *htb1-G52D* (YGF416), *ubp8*Δ *htb1-P102L* (YGF417), *shf1*Δ *ubp8*Δ *htb1-G52D* (YGF432), and *shf1*Δ *ubp8*Δ *htb1-P102L* (YGF433) cells. *J*, the growth of *htb1-G52D* (YGF277), *htb1-P102L* (YGF279), and *htb1-G52D P102L* (YGF491) in the presence of 33 °C, MMS, bleomycin, phleomycin, and UV. *K*, the growth of *htb1-K119R* (YGF226), *htb1-G52D* (YGF277), *htb1-P102L* (YGF279), *htb1-G52D K119R* (YGF444), and *htb1-P102L K119R* (YGF442) cells in the presence of 36 °C, MMS, bleomycin, phleomycin, and UV.

To further dissect the direct mechanism by which the *htb1-G52D* and *htb1-P102L* mutations cause the defect in HR repair, we attempted to isolate *ts* suppressors of the *htb1-G52D* mutant and determined whether the *ts* suppressors also rescued its genotoxic sensitivity or genomic instability. We isolated several *htb1-G52D* colonies that grew at the restrictive temperature of 36 °C due to spontaneous mutations. After purifying these colonies, they were subjected to the spot assay for suppressor confirmation and classification of suppression extent. After PCR and sequencing the *htb1*^+^ gene to exclude the possible revertant of *htb1-G52D* itself and intragenic mutations, we selected some strong extragenic suppressors. Tetrad dissection analysis suggested that these extragenic suppressors were likely single gene mutations. Genome re-sequencing revealed that the strongest suppressor was an insertion of a stop codon (TAGTGGTTTTTG nucleotides) behind the amino acid Leu408 in the ubiquitin protease domain of the deubiquitinase Ubp8. This suppressor was named as *ubp8-1* (Fig. S7*A*). It was a nonsense mutation that presumably caused the loss of the deubiquitination activity. We found that *ubp8-1* rescued the defective growth of *htb1-G52D* cells in the presence of 36 °C, bleomycin, phleomycin, and UV (Fig. 7*A*). To confirm this result and test whether this also applies to *htb1-P102L*, we introduced the null mutation of *ubp8*^*+*^ into our *htb1-G52D* and *htb1-P102L* background strains. The deletion of *ubp8*^*+*^ also suppressed the sensitivities of the *htb1-G52D* and *htb1-P102L* mutants to restrictive temperature (36 °C), MMS, bleomycin, phleomycin, and UV (Figs. 7*B* and S7*B*).

Ubp8 is the catalytic component of the deubiquitination (DUB) module (Ubp8, Sgf11, Sgf73, and Sus1) in the SAGA complex, which is responsible for deubiquitinating H2Bub (58, 59). Since other modules in the SAGA complex and other components of the DUB module are required for the deubiquitination activity of Ubp8 (60), we studied the effects of perturbation of the histone acetylation (HAT) module and Sgf11 in the DUB module on the phenotypes of the *htb1-G52D* and *htb1-P102L* cells. The absence of Gcn5, the catalytic subunit of the HAT module, suppressed the response of the *htb1-G52D* and *htb1-P102L* cells to restrictive temperature and DNA damage (Fig. 7*C*). Because Gcn5 in the HAT module of the SAGA complex acetylates histone H3 and H2B, the suppression mechanism of *gcn5*Δ is possibly attributed to the elevated acetylation levels of H3 and H2B in the *htb1-G52D* and *htb1-P102L* cells. However, the levels of H3K9ac and H2BK5acK10acK15ac were not altered in the *htb1-G52D* and *htb1-P102L* mutants (Fig. S7*C*). This implies that suppression of *htb1-G52D* and *htb1-P102L* mutants depends on the loss of deubiquitination but not the acetylation function of the SAGA complex. In addition to catalytic subunits Ubp8 and Gcn5, non-catalytic subunit Sgf11 interacts with nucleosomal DNA and an acidic patch of the nucleosome and is required for H2BK119 recognition by Ubp8 (61, 62). We found that *sgf11*Δ also reversed the sensitivity of the *htb1-G52D* to restrictive temperature and DNA damage, but failed in *htb1-P102L* cells (Fig. 7*D*). Since the Zinc Finger (ZnF) domain of Sgf11 is responsible for the interaction between the SAGA complex and nucleosome, we made its nonsense mutation *sgf11-Q64∗*, which causes an Sgf11 truncation of ZnF domain (Fig. S7*D*). The *sgf11-Q64∗* mutation rescued the defects of the *htb1-G52D* rather than *htb1-P102L* in response to restrictive temperature and DNA damage (Fig. 7*E*). Furthermore, we constructed a missense mutation *sgf11-R85A* (Fig. S7*D*), which occurs in the ZnF domain and disrupts the interaction between Sgf11 and the nucleosome without affecting the integrity of the SAGA complex (61). The *sgf11-R85A* mutation also suppressed the defects of the *htb1-G52D* instead of *htb1-P102L* in response to restrictive temperature and DNA damage (Fig. 7*F*). These findings suggest that the *htb1-G52D* mutation, not *htb1-P102L*, affects the interaction between the DUB module of SAGA complex and the nucleosome. These suppressions were not due to the detrimental effects of expression levels of *ubp8*^+^, *gcn5*^+^, and *sgf11*^+^ in the *htb1-G52D* and *htb1-P102L* cells, given that the abundance of *ubp8*^+^, *gcn5*^+^ and *sgf11*^+^ transcripts was not significantly altered in the *htb1-G52D*, *htb1-P102L*, and even other H2B onco-mutants (Table S7).

Previous work indicated that H2B appeared to be the only deubiquitinating substrate of Ubp8 in the SAGA complex in *S. pombe* (63). Hence, we reasoned that the restoration of H2Bub abundance is required for the inactivation of Ubp8 to suppress the *htb1-G52D* and *htb1-P102L* mutants. To test this hypothesis, we additionally deleted *shf1*^+^, the adaptor subunit of the H2Bub ligase complex HULC (Rhp6, Brl1, Brl2, and Shf1), resulting in a complete loss of H2Bub (64). As expected, the phenotypes of *shf1*^+^ deletion mutant *shf1*Δ were similar to that of the mutant lacking H2Bub. Besides, the *htb1-G52D* and *htb1-P102L* mutants shared similar phenotypes with *shf1*Δ (Fig. 7, *G* and *H*), suggesting that they also affect H2Bub. Interestingly, we found that *shf1*Δ alleviated the suppression effects of *ubp8*Δ (Figs. 7*G* and S7*E*) and *gcn5*Δ (Fig. 7*H*) on the restrictive temperature and DNA damage sensitivities of the *htb1-G52D* and *htb1-P102L* cells. In addition, the HR frequencies of *htb1-G52D* and *htb1-P102L* were reduced like *shf1*Δ, and restored in the absence of *ubp8*^+^, but reduced again after further deleting *shf1*^+^ (Fig. 7*I*). Collectively, these genetic data motivate us to hypothesize that the restoration of H2Bub is the major reason of the suppression of the *htb1-G52D* and *htb1-P102L* mutants, and the deficiency of H2Bub is the key mechanism for their genomic instability.

If H2Bub is the common effector of the *htb1-G52D* and *htb1-P102L* mutations, we speculate that the *htb1-G52D P102L* double mutant would have more severe defects than either single mutant. We did find that it exhibited the synergistic sensitivity to semi-restrictive temperature and DNA damage (Fig. 7*J*), supporting that the two onco-mutations have distinct mechanisms for reducing H2Bub. Next, we asked whether affecting H2Bub is the only mechanism underlying the *htb1-G52D* and *htb1-P102L* mutants by combining them with the null mutant of H2Bub *htb1-K119R*. As expected, the phenotypes of *htb1-G52D* and *htb1-P102L* were less severe than that of *htb1-K119R*. The phenotype of the *htb1-P102L K119R* double mutant resembled that of *htb1-K119R*, implying that H2Bub is the dominant effector for the *htb1-P102L* mutation. Unexpectedly, the *htb1-G52D* combined with the *htb1-K119R* exhibited enhanced sensitivity to restrictive temperature and DNA damage (Fig. 7*K*), suggesting that affecting H2Bub is not the sole mechanism for the phenotype of the *htb1-G52D* mutant. Since the *htb1-G52D* is a strong *ts* mutant and affects the chromatin structure, we speculate that the defects in both H2Bub and chromatin structure are responsible for the genomic instability of the *htb1-G52D* mutant.

### The levels of H2Bub are reduced in the *htb1-G52D* and *htb1-P102L* mutants and recovered in their SAGA suppressor mutants

**Figure 8:**
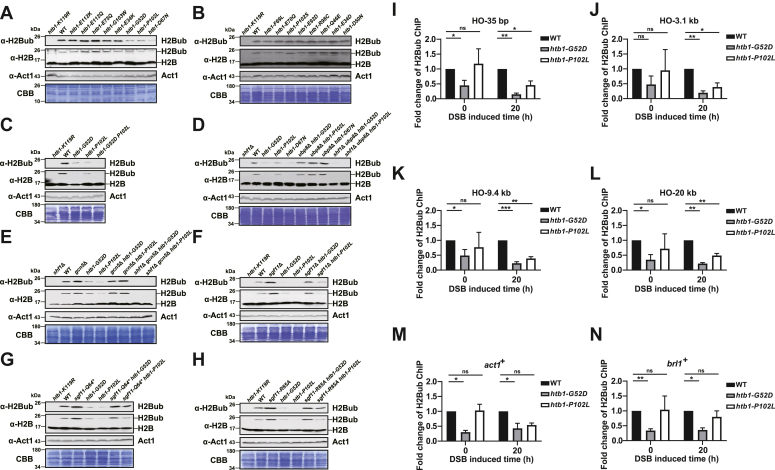
**Global and local levels of H2Bub in the indicated H2B onco-mutants and relevant suppressor mutants.***A*, immunoblots of H2Bub levels in *htb1-K119R* (YGF226), WT (TK8), *htb1-E112K* (YGF328), *htb1-E112Q* (YGF321), *htb1-E75Q* (YGF317), *htb1-G103W* (YGF318), *htb1-E34K* (YGF275), *htb1-G52D* (YGF277), *htb1-P102L* (YGF279) and *htb1-D67N* (YGF324) cells. *B*, the immunoblots of H2Bub levels in *htb1-K119R* (YGF226), WT (TK8), *htb1-F69L* (YGF323), *htb1-E70Q* (YGF330), *htb1-P102S* (YGF325), *htb1-E92D* (YGF326), *htb1-R98C* (YGF322), *htb1-Q46E* (YGF331), *htb1-E34D* (YGF329) and *htb1-D50N* (YGF332) cells. *C*, the immunoblots of H2Bub levels in *htb1-K119R* (YGF226), WT (TK8), *htb1-G52D* (YGF277), *htb1-P102L* (YGF279), and *htb1-G52D P102L* (YGF491) cells. *D*, immunoblots of H2Bub levels in *shf1*Δ (YGF431), WT (TK8), *htb1-G52D* (YGF277), *htb1-P102L* (YGF279), *htb1-D67N* (YGF324), *ubp8*Δ *htb1-G52D* (YGF416), *ubp8*Δ *htb1-P102L* (YGF417), *ubp8*Δ *htb1-D67N* (YGF443), *shf1*Δ *ubp8*Δ *htb1-G52D* (YGF432), and *shf1*Δ *ubp8*Δ *htb1-P102L* (YGF433) cells. *E*, immunoblots of H2Bub levels in *shf1*Δ (YGF431), WT (TK8), *htb1-G52D* (YGF277), *htb1-P102L* (YGF279), *gcn5*Δ (BN1), *gcn5*Δ *htb1-G52D* (YGF418), *gcn5*Δ *htb1-P102L* (YGF419), *shf1*Δ *gcn5*Δ *htb1-G52D* (YGF457), and *shf1*Δ *gcn5*Δ *htb1-P102L* (YGF458) cells. *F*, immunoblots of H2Bub levels in *htb1-K119R* (YGF226), WT (TK8), *sgf11*Δ (YGF482), *htb1-G52D* (YGF277), *htb1-P102L* (YGF279), *sgf11*Δ *htb1-G52D* (YGF483), and *sgf11*Δ *htb1-P102L* (YGF484) cells. *G*, immunoblots of H2Bub levels in *htb1-K119R* (YGF226), WT (TK8), *sgf11*-*Q64∗* (YGF485), *htb1-G52D* (YGF277), *htb1-P102L* (YGF279), *sgf11*-*Q64∗ htb1-G52D* (YGF486), and *sgf11*-*Q64∗ htb1-P102L* (YGF487) cells. *∗* indicates the stop codon. *H*, immunoblots of H2Bub levels in *htb1-K119R* (YGF226), WT (TK8), *sgf11*-*R85A* (YGF488), *htb1-G52D* (YGF277), *htb1-P102L* (YGF279), *sgf11*-*R85A htb1-G52D* (YGF489), and *sgf11*-*R85A htb1-P102L* (YGF490) cells. *I*–*N*, the ChIP-qPCR analysis of H2Bub enrichment at 35 bp (*I*), 3.1 kb (*J*), 9.4 kb (*K*), and 20 kb (*L*) adjacent to the HO-induced DSB site, as well as at *act1*^+^ (*M*) and *brl1*^+^ (*N*) gene bodies after HO-induced 0 h or 20 h. The fold change of H2Bub enrichment in the *htb1-G52D* (YGF276) and *htb1-P102L* (YGF278) relative to WT (DY2407) is shown at HO-induced 0 h and 20 h, separately. The data from two or three independent biological repeats are averaged. Error bars represent SEMs. One-way ANOVA is used for multiple comparisons between the indicated samples and WT at HO-induced 0 h or 20 h.

The above genetic data prompted us to investigate the levels of H2Bub in our 16 H2B onco-mutants (Fig. 8, *A* and *B*). As expected, we found that the levels of H2Bub were decreased in the *htb1-G52D* and *htb1-P102L* mutants (Fig. 8*A*), and were additively reduced in the *htb1-G52D P102L* double mutant (Fig. 8*C*). Besides *htb1-G52D* and *htb1-P102L*, the levels of H2Bub were also reduced in the *htb1-D67N* mutant (Fig. 8*A*), which was also ranked among the top 20 of H2B mutations by frequency in a previous report (8). The deletion of *ubp8*^+^ also rescued the restrictive temperature and genotoxic sensitivities of the *htb1-D67N* mutant (Fig. S8*A*). Since the growth of the *htb1-D67N* cells was so weak that its transformation efficiency was very low, its frequency of HR could not be reliably calculated. These data suggest that the H2Bub-dependent mechanism for genomic instability applies to other H2B onco-mutants besides H2BG52D and H2BP102L.

Furthermore, immunoblots revealed that the levels of H2Bub were significantly restored when the *htb1-G52D*, *htb1-P102L*, and *htb1-D67N* were combined with *ubp8*Δ, but completely reduced when *shf1*^+^ was additionally deleted (Fig. 8*D*). Similar results of immunoblots were obtained for the suppressor *gcn5*Δ (Fig. 8*E*). For the SAGA non-catalytic subunit Sgf11, we also found that the *sgf11*Δ (Fig. 8*F*), *sgf11-Q64∗* (Fig. 8*G*), and *sgf11-R85A* (Fig. 8*H*) in the *htb1-G52D* significantly rescued the reduced levels of H2Bub to that of *sgf11* mutants, but failed in the *htb1-P102L* cells. Together, these provide biochemical evidence that the genomic instability and its suppression in the *htb1-G52D* and *htb1-P102L* cells is due to the loss of H2Bub and the recovery of H2Bub, respectively.

In addition to the global reduction of H2Bub levels in whole-cell protein extracts, we speculate that the levels of H2Bub around the local DNA break site are also reduced in the *htb1-G52D* and *htb1-P102L* cells because RPA recruits Bre1 (Brl1 homolog in budding yeast) to catalyze the initial DNA break-proximal H2Bub in budding yeast and Wdr70 promotes the spreading of Brl1/Brl2-mediated break-distal (several kilobases away) H2Bub in fission yeast (65, 66). To test this speculation, we performed the H2Bub ChIP experiments. Our preliminary results indicated that the antibody against H2Bub was valid for ChIP (Fig. S8*B*). We then found that the levels of H2Bub in the *htb1-G52D* and *htb1-P102L* cells were significantly reduced at 35 bp, 3.1 kb, 9.4 kb, and 20 kb away from the HO-induced DSB site (Fig. 8, *I*–*L*), but not at *centromere-I* and the intergenic region *ars2004*, which are not transcribed and lacking H2Bub (Fig. S8, *C* and *D*). Since H2Bub is widespread across the transcribed genes, we also found that the levels of H2Bub in the *htb1-G52D* cells were significantly reduced at the transcribed genes, such as *act1*^+^ and *brl1*^+^ (Fig. 8, *M* and *N*). Taken together, we conclude that the abundance of H2Bub is reduced at global and local DNA damage sites in the *htb1-G52D* and *htb1-P102L* cells.

### The recruitment of the SAGA but not the HULC complex is responsible for the reduction of H2Bub in the *htb1-G52D* mutant

Furthermore, we investigated the possible mechanism responsible for H2Bub reduction in the *htb1-G52D* and *htb1-P102L* mutants. The reduction of H2Bub could be caused by the loss of the ubiquitination function of the HULC complex and the gain of the deubiquitination function of the SAGA complex. Our RNA-seq data revealed that the RNA levels of the HULC complex subunits Rhp6, Brl1, and Brl2 were not significantly decreased (Table S7), and the protein levels of Rhp6, Brl1, Brl2, and Shf1 were also not altered in the *htb1-G52D* and *htb1-P102L* mutants (Fig. S9*A*). Additionally, we speculate that the ubiquitination activity of the HULC complex is not affected by the *htb1-G52D* and *htb1-P102L* mutations because their H2Bub are completely catalyzed by the HULC complex after inactivation of deubiquitination. To test this hypothesis, we overexpressed Brl1 (ubiquitin ligase E3) from the *adh21*^*+*^ promoter with medium strength (Fig. S9*B*), and found that it failed to suppress the restrictive temperature and genotoxic sensitivities of the *htb1-G52D* and *htb1-P102L* cells (Fig. S9*E*). Furthermore, we overexpressed Brl1 from the *nmt1*^*+*^ promoter with higher strength (Fig. S9*C*) and obtained a similar result (Fig. S9*F*). We also overexpressed Rhp6 (ubiquitin-conjugating enzyme E2) from the *nmt1*^*+*^ promoter (Fig. S9*D*) and still found that their defects were not rescued (Fig. S9*G*). These genetic data suggest that the reduction of H2Bub in the *htb1-G52D* and *htb1-P102L* cells might not be attributed to decreased activity of their HULC complexes.

**Figure 9:**
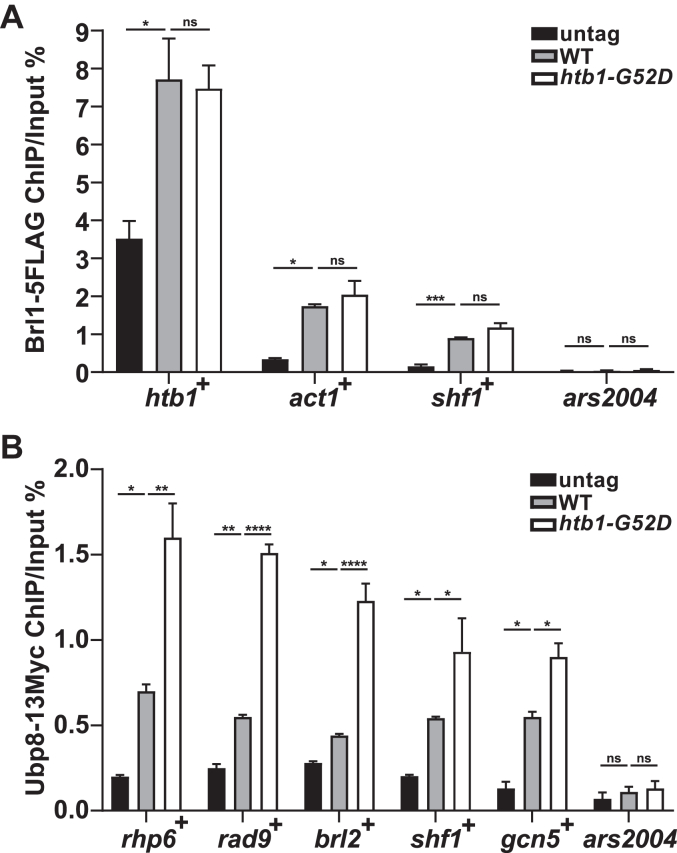
**Ubp8 in the SAGA complex but not Brl1 in the HULC complex exhibits enhanced recruitmentonto the chromatin in the *htb1-G52D* mutant.***A*, the ChIP-qPCR of Brl1-5FLAG enrichment at *htb1*^+^, *act1*^+^, *shf1*^+^, and *ars2004*. The Brl1-5FLAG enrichments from the untag (TK8), WT (YGF479), and *htb1-G52D* (YGF496) are shown as the percentages of IP DNA relative to input DNA. *B*, the ChIP-qPCR of Ubp8-13Myc enrichment at *rhp6*^+^, *rad9*^+^, *brl2*^+^, *shf1*^+^, *gcn5*^+^, and *ars2004* from the untagged control (TK8), WT (YGF502), and *htb1-G52D* (YGF503). The data from two or three independent biological repeats are averaged. Error bars represent SEMs. One-way ANOVA is used for multiple comparisons between the indicated samples and WT.

Since the defects of the *htb1-G52D* but not the *htb1-P102L* mutant are rescued by mutations of *sgf11*^+^, and the *htb1-G52D P102L* double mutant exhibits the synthetic defects, we hypothesize that the molecular details underlying H2Bub reduction may be distinct between the *htb1-G52D* and *htb1-P102L* mutants, and the *htb1-G52D* but not *htb1-P102L* mutant may be defective in association of the HULC or the SAGA complex with the nucleosomes. Consequently, we first tested whether the recruitment of the HULC complex subunit Brl1 onto nucleosomes was affected by the *htb1-G52D* mutation. We validated the Brl1-5FLAG ChIP experiment by demonstrating that Brl1-5FLAG in the WT was enriched at the active gene *htb1*^+^ more than that of untagged control (Fig. S9*H*). We then repeated Brl1-5FLAG ChIP and found that Brl1-5FLAG in the *htb1-G52D* was not enriched at non-transcribed region *ars2004* as expected, but similarly enriched at the indicated transcribed genes as it was in the WT (Fig. 9*A*).

In addition to the HULC complex, we investigated whether the SAGA complex was influenced by the *htb1-G52D* and *htb1-P102L* mutations. The RNA levels of Ubp8, Sgf11, Sgf73, and Sus1 in the DUB module of the SAGA complex were not significantly changed (Table S7), and the protein levels of Ubp8 and Sgf11 were also not altered in the *htb1-G52D* and *htb1-P102L* mutants (Fig. S9*I*). Moreover, the H2B deubiquitination activity of Ubp8 in the SAGA complex may not be directly affected by the *htb1-G52D* and *htb1-P102L* mutations, since they are not close to the H2BK119 site. Thus, we speculate that they may indirectly influence the regulator subunits of the DUB module in SAGA. To test whether the interaction between Ubp8 and its substrate was affected, we performed Ubp8-13Myc ChIP and confirmed that the 13Myc tag did not interfere with the levels and H2B deubiquitinating activity of Ubp8 in the WT and *htb1-G52D* cells (Fig. S9, *I* and *J*). We verified that Ubp8-13Myc in the WT was significantly enriched at the indicated transcribed genes more than that of the untagged control (Fig. S9*K*). We then repeated Ubp8-13Myc ChIP and discovered that Ubp8-13Myc of the *htb1-G52D* was not significantly enriched at the non-transcribed region *ars2004* as expected, but enriched at the indicated transcribed genes more than that of WT (Fig. 9*B*). These results suggest that the recruitment of the SAGA but not HULC complex onto the chromatin is enhanced in the *htb1-G52D* mutant.

The above results motivate us to propose that the 52nd amino acid of H2B changing from Glycine (G) to Aspartic acid (D) is critical for the enhanced H2BK119 recognition by the DUB module in the SAGA complex. Indeed, the other H2BG52 onco-mutants such as the *htb1-G52A* and *htb1-G52S*, which are mutated to neutral amino acids, exhibited no significant defects in restrictive temperature and MMS sensitivities (Fig. S9*L*) as well as H2Bub levels (Fig. S9*M*). The *htb1-G52R* onco-mutant, which is mutated to a basic amino acid, also exhibited no defects (our unpublished data). Conversely, the *htb1-G52E* onco-mutant, which is mutated to another acidic amino acid, exhibited similar defects in genome stability and H2Bub levels (our unpublished data). These suggest that the negative charge of H2BG52D has an important impact on H2Bub and the association between the SAGA complex and the nucleosomes.

### Heterozygous H2BG52D and H2BP102L mutations exhibit MMS sensitivities and reduction of H2Bub in *cis*

**Figure 10:**
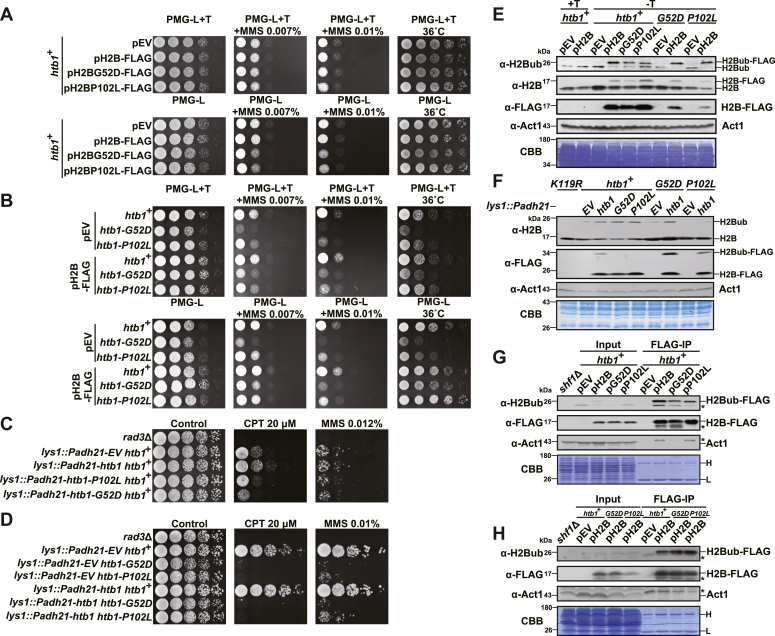
**The DNA damage sensitivities and H2Bub levels of the heterozygous H2BG52D and H2BP102L mutants.***A*, MMS and temperature sensitivities of *htb1*^+^/pEV (YGF311), *htb1*^+^/pH2B-FLAG (YGF314), *htb1*^+^/pH2BG52D-FLAG (YGF319), and *htb1*^+^/pH2BP102L-FLAG (YGF320) strains. pH2BG52D-FLAG indicates the FLAG tagged *htb1-G52D* gene under control of *nmt1*^*+*^ promoter in pREP1; pH2BP102L-FLAG indicates the FLAG tagged *htb1-P102L* gene under control of *nmt1*^*+*^ promoter in pREP1. *B*, MMS and temperature sensitivities of *htb1*^+^/pEV (YGF311), *htb1-G52D*/pEV (YGF312), *htb1-P102L*/pEV (YGF313), *htb1*^+^/pH2B-FLAG (YGF314), *htb1-G52D*/pH2B-FLAG (YGF315), and *htb1-P102L*/pH2B-FLAG (YGF316) strains. pEV is the empty vector of pREP1 plasmid. pH2B-FLAG indicates the C-terminal FLAG tagged *htb1*^+^ gene under control of *nmt1*^*+*^ promoter in pREP1. T indicates thiamine. *C*, MMS and CPT sensitivities of *htb1*^+^ strains integrated with overexpressed *Padh21-EV* (YGF467), *Padh21-htb1-5FLAG* (YGF493), *Padh21-htb1-G52D-5FLAG* (YGF498), and *Padh21-htb1-P102L-5FLAG* (YGF500) at *lys1*^+^. *D*, MMS and CPT sensitivities of *htb1*^+^ (YGF493), *htb1-G52D* (YGF494), and *htb1-P102L* (YGF495) strains integrated with overexpressed *Padh21-htb1-5FLAG* at *lys1*^+^. *htb1*^+^ (YGF467), *htb1-G52D* (YGF422), and *htb1-P102L* (YGF424) strains are also integrated with *Padh21-EV* at *lys1*^+^. *E*, immunoblots of H2Bub and H2Bub-FLAG in *htb1*^+^, *htb1-G52D*, and *htb1-P102L* strains with H2B-FLAG, H2BG52D-FLAG, and H2BP102L-FLAG overexpression. pH2B, pG52D, and pP102L are abbreviations for pH2B-FLAG, pH2BG52D-FLAG, and pH2BP102L-FLAG, respectively. *F*, the immunoblots of H2Bub and H2Bub-FLAG in *htb1*^+^ strains integrated with overexpressed H2B-5FLAG, H2BG52D-5FLAG, and H2BP102L-5FLAG, as well as in *htb1-G52D* and *htb1-P102L* strains integrated with overexpressed H2B-5FLAG. *G*, the immunoblots of H2Bub-FLAG in the FLAG immunoprecipitate from the *htb1*^+^ strain transformed with plasmids of H2B-FLAG, H2BG52D-FLAG, and H2BP102L-FLAG. *H*, the immunoblots of H2Bub-FLAG in the FLAG immunoprecipitate from *htb1*^+^, *htb1-G52D*, and *htb1-P102L* strains transformed with the H2B-FLAG plasmid. The asterisk indicates cross-reactive protein bands. H and L indicate heavy and light chains of antibodies, respectively.

The above findings of *htb1-G52D* and *htb1-P102L* in *S. pombe* prompted us to ask whether they apply to humans. Since H2B mutations in human cancers occur in one of 18 copies of the classical H2B gene, we attempted to mimic this genotype in *S. pombe* by constructing heterozygous H2BG52D and H2BP102L mutants and tested their MMS sensitivities and H2Bub levels. Thus, we constructed the first type of heterozygous H2BG52D and H2BP102L mutants *via* overexpressing the FLAG-tagged H2BG52D and H2BP102L plasmids after depletion of thiamine in the *htb1*^*+*^ strain. These heterozygous strains conferred the MMS but not the temperature sensitivity (Fig. 10*A*). Reversely, we constructed the second type of heterozygous H2BG52D and H2BP102L mutants by overexpressing the FLAG-tagged H2B plasmid after depletion of thiamine in the *htb1-G52D* and *htb1-P102L* strains. We found that the overexpression of H2B rescued the *ts* phenotype but not the MMS sensitivity of the *htb1-G52D* and *htb1-P102L* mutants (Figs. 10*B* and S10*A*), supporting again that H2B or nucleosome destabilization which is responsible for *ts* may not be the key mechanism for their genomic instability. Overexpression of FLAG-tagged H2B, H2BG52D, and H2BP102L were verified with immunoblots (Fig. S10*B*). To overcome the problem that phenotypes of cells in drugs-containing PMG synthetic medium are weak, we constructed the third type of heterozygous H2BG52D and H2BP102L mutants *via* integrating the plasmids into the *lys1*^+^ locus and overexpressing the FLAG-tagged H2BG52D and H2BP102L from *adh21* promoter in the *htb1*^*+*^ strain. These heterozygous strains conferred the MMS and CPT sensitivities (Fig. 10*C*). We also constructed the fourth type of heterozygous H2BG52D and H2BP102L mutants by integrating and overexpressing the FLAG-tagged H2B in the *htb1-G52D* and *htb1-P102L* strains. These heterozygous strains also displayed significant MMS and CPT sensitivities (Fig. 10*D*). Altogether, these results suggest that the heterozygous H2BG52D and H2BP102L mutations cause genomic instability in a dominant-negative manner.

We then determined the levels of H2Bub in these four types of heterozygous H2BG52D and H2BP102L mutants. In the first type of heterozygotes, we discovered that the amount of exogenous ubiquitinated H2BG52D-FLAG and H2BP102L-FLAG were decreased compared with that of exogenous ubiquitinated H2B-FLAG in the *htb1*^+^ cells. In the second type of heterozygotes, the amount of exogenous ubiquitinated H2B-FLAG in the *htb1*^+^ cells was the same as that of exogenous ubiquitinated H2B-FLAG in the *htb1-G52D* and *htb1-P102L* cells (Fig. 10*E*). In addition, the amount of endogenous ubiquitinated H2BG52D in *htb1-G52D* cells and ubiquitinated H2BP102L in *htb1-P102L* cells was also decreased compared with endogenous ubiquitinated H2B in the *htb1*^+^ cells with overexpressed H2B-FLAG (Fig. 10*E*). Consistent results were obtained in the third and fourth type of heterozygotes with integration and overexpression of H2B, H2BG52D, and H2BP102L (Fig. 10*F*). Therefore, we conclude that the abundance of H2Bub is reduced when the G52D and P102L are mutated in the same H2B protein.

To further support the above mentioned results obtained from whole-cell protein extracts, overexpressed H2B-FLAG, H2BG52D-FLAG, and H2BP102L-FLAG in *htb1*^*+*^ cells (the first type of heterozygotes) were immunoprecipitated by FLAG beads. We found that the H2Bub abundance was significantly reduced in both input and immunoprecipitated H2BG52D-FLAG and H2BP102L-FLAG compared with that of H2B-FLAG (Fig. 10*G*). Reversely, overexpressed H2B-FLAG in *htb1*^*+*^, *htb1-G52D*, and *htb1-P102L* cells (the second type of heterozygotes) were also immunoprecipitated and the abundance of H2Bub was unaffected in both input and immunoprecipitated H2B-FLAG from *htb1-G52D* and *htb1-P102L* cells (Fig. 10*H*). These two IP results were reproducible in another independent transformant, respectively (Fig. S10, *C* and *D*). Together, these data support that the heterozygous H2BG52D and H2BP102L mutations reduce H2Bub in *cis*.

### Genomic instability of the *htb1-G52D* and *htb1-P102L* mutants are independent of Set1 mediated H3K4 methylation

Since H2Bub has the crosstalk with H3K4 methylation (H3K4me) (40, 67, 68), we finally tested whether H3K4 methylation, in addition to H2Bub, was also responsible for genomic instability of the *htb1-G52D* and *htb1-P102L* mutants. We uncovered that the abundance of both H3K4me2 and H3K4me3 was diminished in the *htb1-G52D* and *htb1-P102L* cells (Fig. S11*A*). Nevertheless, the *htb1-G52D* and *htb1-P102L* mutations did not affect the total abundance of Set1 proteins (Fig. S11*B*). Moreover, overexpression of Set1 failed to suppress the MMS sensitivities of the *htb1-G52D* and *htb1-P102L* mutants (Fig. S11*C*), although overexpression of Set1 did not significantly restore the levels of H3K4me3 in the *htb1-G52D* and *htb1-P102L* mutants (Fig. S11*D*). As previously reported (69), the null mutation of *set1*^*+*^ was not significantly sensitive to MMS, which was distinct from the phenotypes of *htb1-G52D* and *htb1-P102L* mutants. The absence of *set1*^*+*^ exaggerated the MMS sensitivities of the *htb1-G52D* and *htb1-P102L* mutants (Fig. S11*E*), implying again that *set1*^+^ affects distinct genes or pathways in response to MMS. Additionally, the gene expression profile of *set1*Δ was distinct from that of *htb1-G52D* and *htb1-P102L* (Fig. S11*F*), and the number of genes affected by *set1*Δ in GO terms was also different from the number affected by *htb1-G52D* and *htb1-P102L* (Fig. S11*G*). Together, these data suggest that the genomic instability of the *htb1-G52D* and *htb1-P102L* mutants is independent of Set1 or H3K4 methylation. This is reminiscent of the findings that the functions of H2Bub in transcription elongation and HR repair are also unrelated to H3K4 methylation mediated by Set1 (40, 56).

## Discussion

**Figure 11:**
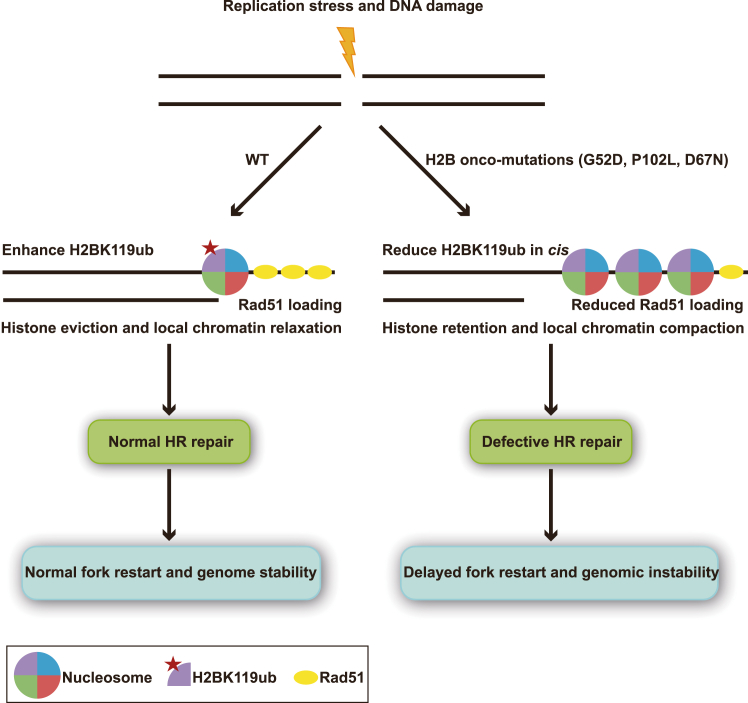
**A model for the genomic instability in H2B onco-mutants of *S. pombe*.** Replication stress and DNA damage increase the abundance of H2Bub to promote histone eviction and chromatin relaxation around DNA break sites, which facilitates the Rad51 recruitment and HR repair as well as genome stability in WT cells. In contrast, the H2B onco-mutations such as G52D, P102L, and D67N in *S. pombe* reduce the global and local abundance of H2Bub in *cis*. The loss of H2Bub leads to defective histone eviction and chromatin compaction at local DNA break sites. This compromises the Rad51 recruitment and HR repair and finally causes genomic instability.

We have characterized H2B oncohistones in *S. pombe*. The genomic instability caused by H2B oncohistones supports that noncanonical oncohistones may act as disease drivers rather than passengers. Noncanonical histone onco-mutations are thought to likely affect histone PTMs, but the evidence is lacking. Here we discovered that H2BG52D, H2BP102L, and H2BD67N onco-mutations in *S. pombe* reduced H2Bub, which plays a key role in HR repair by facilitating histone eviction and chromatin relaxation (56, 70). Thus, local histone retention and chromatin compaction impair Rad51 loading onto resected DNA break ends. This leads to defects in HR repair and genomic instability (Fig. 11). This model could also apply to heterozygous H2BG52D and H2BP102L. Therefore, we propose that affecting H2Bub is a novel mechanism of cancers with H2B mutations, and affecting histone PTMs is also a potential oncogenic mechanism of other noncanonical oncohistones.

Our survey of 16 H2B oncohistones in *S. pombe* revealed a significant association between *ts* and genomic instability of H2B onco-mutants. Seven H2B onco-mutations with *ts* directly destabilize nucleosome: H2BE75Q interferes with the H2B-H4 interaction (8, 9); H2BE112K disturbs the formation of nucleosome acidic patch (8); H2BG52D and H2BE34K could weaken H2B-DNA interaction (57, 71); H2BP102L and H2BD67N may be defective in the interaction with H2A (71). Therefore, nucleosome destabilization contributes to phenotypes of H2B oncohistones, as previously reported (32). However, this study uncovered that the genomic instability of *htb1-G52D*, *htb1-P102L*, and *htb1-D67N* is largely due to compromised H2Bub. Since their H2Bub levels correlate well with their severity of phenotypes and the recovery of H2Bub significantly rescues their genotoxic sensitivities and HR defects, we propose that besides nucleosome destabilization, affecting H2Bub is a parallel and dominant pathway for the genomic instability of some H2B onco-mutants. The fact that *htb1-K119R* showed negative genetic interaction with *htb1-G52D* further supports that H2Bub-dependent and -independent pathways act in parallel to maintain genome stability. Moreover, a similar study isolated 56 *ts* suppressors of the *S. pombe* *htb1-G52D* mutant and obtained 16 distinct *ts* suppressors. All these mutants were subunits of the SAGA complex: Ubp8, Gcn5, Hfi1/Ada1, Sgf11, and Sus1, but not other mutants implicated in chromatin remodeling (60). Although deubiquitination and chromatin remodeling functions of the SAGA complex are intertwined, these highly specific and convergent suppressors support that affecting H2Bub is the major mechanism for the genomic instability in *htb1-G52D* cells.

The DNA damage sensitivity of cancer-irrelevant histone mutations like H3K56R can be suppressed by H3 overexpression (72). However, the MMS sensitivities of H2BG52D and H2BP102L cannot be rescued by H2B overexpression, although their *ts* phenotypes can be rescued. Importantly, the DNA damage sensitivity and HR ability of H2BG52D and H2BP102L can be largely rescued by the inactivation of the Ubp8 and Sgf11 in the SAGA DUB module that results in restored H2Bub levels. These results support that the biological effect of noncanonical oncohistones is also dominant-negative.

There are several possibilities to explain how HR repair in H2B onco-mutants is inhibited by reduced H2Bub. First, H2Bub directly alters nucleosome stability and disrupts chromatin compaction (70, 73, 74). Second, H2Bub indirectly facilitates histone eviction by cooperating with the histone chaperone FACT to remove H2A-H2B dimer as it does in transcription (75, 76, 77, 78). Finally, H2Bub indirectly promotes chromatin relaxation to regulate HR repair *via* recruiting ATP-dependent chromatin remodelers like the SWI/SNF complex (42, 79, 80, 81). Therefore, local histone retention and chromatin compaction caused by H2Bub reduction in H2B onco-mutants may impair the recruitment of repair proteins like Rad51 to DNA damage sites (56, 82). To prove these possibilities, we need to study whether the defect of histone or nucleosome eviction and Rad51 loading of the H2BG52D and H2BP102L mutants can be suppressed by the inactivation of the Ubp8 or SAGA complex.

H2Bub has crosstalk with histone PTMs such as H3K4me (68) and the acetylation of H2A (83). H2Bub is also affected by other histone residues, *e*.*g*., H2A N-terminal repression domain, H4 basic patch, and H2A/H2B acidic patch (84, 85, 86). Nevertheless, how H2Bub is reduced in *cis* by the H2BG52D, P102L, and D67N onco-mutations is still unclear in detail. Our results suggest that these onco-mutations may indirectly regulate the H2B deubiquitination function of the DUB module in the SAGA complex. Structural studies suggest that Sgf11 and Ubp8 interact with nucleosomal DNA (61, 62). The H2BG52D mutation promotes the association of Ubp8 with the nucleosome. Accordingly, we hypothesize that H2BG52 regulates H2Bub through competing with Ubp8 and/or Sgf11 for association with the nucleosomal DNA, and the H2BG52D mutation weakens its interaction with DNA but strengthens the interaction of nucleosome with Ubp8 and/or Sgf11. We also speculate that H2BP102 and H2BD67 may affect nucleosome stability and higher-order chromatin structure, and the H2BP102L and H2BD67N mutations may destabilize chromatin structure and release additional nucleosomes for SAGA binding and deubiquitination. It will be interesting to test these hypotheses in future studies.

H2BG52D and H2BP102L were reported to affect chromosome segregation and centromere silencing at the restrictive temperature in *S. pombe* (71), but they were not recognized as oncohistones and studied for DNA damage repair. Moreover, H2BG53D mutation was knocked into one H2B gene (*HIST1H2BO*) in the pancreatic ductal adenocarcinoma cell line S2VP10 (57, 87), which has a relatively lower mutation frequency in human cancers (9). The genomic instability and the change in γH2A and H2Bub abundance were not detected in human cells (57), presumably because of the weak effect of the G53D mutation in one of 18 H2B genes. However, the homozygous mutation in the single H2B gene in our haploid fission yeast may amplify the effects of H2B oncohistones to be detectable. Moreover, our study of the heterozygous H2BG52D and H2BP102L mutants further supports the possibilities of genomic instability and local H2Bub reduction in human cells. Thus, changing the H2B mutated gene copy, types of cell lines, and local H2Bub detection methods like ChIP-seq could be necessary to confirm the effects of H2BG53D and H2BP103L in human cells.

In summary, the *S. pombe* model of H2B oncohistones demonstrates that H2B onco-mutations have effects on genotoxic response, HR repair, and genome stability. Inactivation of the Ubp8 in the SAGA complex can suppress genomic instability defects in H2B oncohistones by reversing the reduced levels of H2Bub. Therefore, we propose that the oncogenic mechanism of H2B oncohistones may be dependent on H2Bub. The inhibitors of USP22, which is the human homolog of Ubp8 and a death-from-cancer signature (88), might treat cancers with H2B oncohistones. In addition, we need to investigate whether other PTMs of H3, H4, H2B, and H2A could be influenced by their noncanonical onco-mutations.

## Experimental procedures

### *S. pombe* strains, plasmids, and antibodies

Standard genetic methods were used for the construction of *S. pombe* strains (89, 90, 91), which are listed in Table S1. The information on plasmids and antibodies is listed in Tables S2 and S3, respectively.

### *S. pombe* growth and survival assay

*S. pombe* strains were grown at 30 °C, unless other permissive or restrictive temperatures were indicated. For the growth assay, mid-log phase cells were resuspended to the same optical density (OD_600_) 0.5 and serially 5-fold diluted. Each dilution was spotted onto YES plates at the indicated temperatures and plates containing drugs for chronic exposure at 30 °C. The drugs include hydroxyurea (HU) (Sigma, H8627), camptothecin (CPT) (Sigma, C9911), methyl methanesulfonate (MMS) (Sigma, 129925), phleomycin (MCE, HY-126490), bleomycin (MCE, HY-17565), mycophenolic acid (MPA) (Sigma, M5255) and thiabendazole (TBZ) (Sigma, T8904). Cells were grown for 3 to 5 days and photographed. For the survival assay, mid-log phase cells were acutely exposed to 0.05% MMS for the indicated time points. Cells were washed and neutralized with 5% sodium thiosulphate, serially diluted, and plated onto YES. The number of colonies was counted. The percentage of survival was shown as colony number at the indicated time of MMS exposure divided by the colony number of cells without MMS treatment, which is set as 100% survival.

### RNA-seq and gene expression analysis

Total RNA was extracted from 50 OD_600_ cells using Trizol kit (Invitrogen). mRNA was enriched by oligo(dT) beads and fragmented. cDNA was synthesized, end-repaired, and ligated to Illumina sequencing adapters. The specific purified ligation products were amplified by PCR and sequenced using Illumina Hiseq 2500 by Gene Denovo Biotechnology (Guangzhou, China). Raw reads were filtered by fastp and the rRNA mapped reads were removed by Bowtie2 alignment. The remaining clean reads were mapped to *S. pombe* reference genome (Ensembl_release45) using HISAT2. The read count of each region was transformed to FPKM (fragment per kilobase of transcript per million mapped reads) value as gene expression abundance using StringTie. The transcripts with FDR (false discovery rate) or q value below 0.05 and more than 2-fold change averaged from three independent biological repeats were considered as differentially expressed genes (DEGs). Analysis of bioinformatic data was performed using Omicsmart online platform (http://www.omicsmart.com). All gene expression was listed in Table S4. A list of DEGs is provided in Table S5. A list of the expression of genes involved in DNA replication, repair, and recombination of GO-BP and KEGG is provided in Table S6. Gene expression in the SAGA complex and the HULC complex are listed in Table S7.

### EdU labeling and analysis by flow cytometry (FACS) and immunofluorescence (IF)

The methods of EdU labeling and detection by FACS and IF were modified from previous reports (92, 93, 94, 95). Strains with the ability to incorporate EdU, a nucleoside analog of thymidine, were synchronized into the early S phase after treatment with 12 mM HU for 4 h or by *cdc10ts* block and release. The synchronized and asynchronized cells were labeled with 10 μM 5-ethynyl-2′-deoxyuridine (EdU) (Molecular Probes, E10187) at the indicated time points with and without 0.05% MMS. For the MMS release experiment, MMS was inactivated and removed by repeated washing with 5% sodium thiosulphate. The cells were then fixed with cold 70% ethanol. After washing, cells were reacted with the Click-iT EdU (Alexa Fluor 488) imaging reagents (Molecular Probes, C10337). For the FACS analysis, after briefly sonicating cells, the cell percentage and mean intensities of fully and partially incorporated EdU were analyzed using a FACSVerse (BD) to detect FL1-A (Alexa Fluor 488). The EdU intensity at 0 h of labeling was the background and subtracted from the intensity at each time point. The results of FACS were analyzed using the FlowJo software. For the IF analysis, the cells with Alexa Fluor 488 labeled EdU were then stained with DAPI (Solarbio, C0065) and observed with a laser confocal microscope (Nikon, A1R). The results of IF were analyzed using the NIS-Elements Free Viewer software and the raw images of each representative timepoint were shown in Figs. S12–S15.

### Microscopy imaging

Methanol fixed cells were washed and stained with calcofluor (Sigma, 18909) to measure the septation index, which was calculated as the number of *S. pombe* septa divided by the total number of cells. Methanol-fixed cells were washed and observed with a laser confocal microscope (Leica, SP8) to measure the percentage of Rpa1-YFP and Rad52-2CFP foci.

### Immunoblotting of whole-cell protein extracts

Whole-cell proteins were extracted and immunoblotted according to previous publications (96, 97). 10 OD_600_ cells were resuspended in 500 μl of 10% trichloroacetic acid (TCA) (Sigma, 91228) and 200 μl of acid-washed glass beads (Sigma, G-8772). After bead-beating and centrifugation, the pellets were resuspended in the 2x SDS loading buffer, adjusted pH with 1 M Tris, and boiled at 90 °C for 5 min. After running gel and gel transfer, the blots were detected with SuperSignal West Pico PLUS chemiluminescence (Thermo, 34580) and Odyssey infrared imaging system (Li-Cor). The blots after exposure were stained with Coomassie Brilliant Blue (CBB) to show loading controls with total proteins (98). The ImageJ software was used for the quantification of the signal intensity of protein bands.

### Homologous recombination (HR) frequency assay

The frequency of HR was measured according to the previous method (99). The plasmid pGF77 (pBluescript-*leu1*-3′Δ) was linearized with *Apa*I (NEB, R0114) to generate the integrating fragment containing 5′ homologous region and 3′ truncated homologous region of *leu1*-3′Δ. This was co-transformed with an equal molar of pGF124 (pREP2-*ura4*^+^) into the *leu1-32 ura4-D18* strains. HR frequency was calculated as the number of colonies grown in PMG-Leu plates normalized to the number of colonies grown in PMG-Ura plates. The raw data are listed in Table S8.

### HO endonuclease-induced DSB assay

The assay for the HO-induced DSB was based on these previous publications (53, 54, 55). A single DSB was induced by HO (homing endonuclease) cleavage of the genome. gDNA was extracted and digested with and without *Apo*I (NEB, R3566). The PCR products at the gene *isn1*^*+*^ represent the amount of total DNA, and the PCR products at the HO site represent the amount of uncut DNA. The subtractions of uncut from total DNA were calculated as percentages of HO-induced DSB at indicated times except 0 h. The sequences of primers are listed in Table S9.

### Micrococcal nuclease (MNase) digestion assay

MNase digestion assay was based on a previous method (96). 200 OD_600_ cells were incubated with zymolase-20T (MP Biomedicals, 320921) to digest the cell wall. Spheroplasts were then permeabilized with 0.075% NP-40 buffer and digested with a set of increasing units of MNase (NEB, M0247) at 25 °C for 10 min. After DNA purification, an equal abundance of total DNA was separated on 1.5% agarose gel. The amount of indicated DNA in each lane was quantified with ImageJ software by calculating the integral area of the peak in the optical density plot after background subtraction.

### Chromatin immunoprecipitation and quantitative PCR (ChIP-qPCR)

ChIP-qPCR assay was modified from the previous publication (96). For the ChIPs of Rad51-5FLAG, H2B, H2B-5FLAG, and H2Bub, 50 OD_600_ cells were crosslinked with 3% paraformaldehyde (Sigma, P6148) at 25 °C for 20 min and stopped by the addition of 1/20 volume of 2.5 M glycine. For the ChIPs of Brl1-5FLAG and Ubp8-13Myc, 100 OD_600_ cells were shifted to 18 °C for 6 h prior to crosslinking with 3% paraformaldehyde for 60 min, followed by fixing with 10 mM dimethyl adipimidate (Sigma, 285625) at 25 °C for 45 min. Cells were resuspended in the ChIP lysis buffer (50 mM HEPES (pH 7.2); 150 mM NaCl; 1 mM EDTA; 1% TritonX-100; 0.1% SDC) with yeast protease inhibitors (Sangon, C510026) and disrupted with FastPrep (MP Biomedicals) bead-beating. Chromatin was then sonicated to 200 to 1000 bp of DNA fragments as input. For the ChIPs of H2B and H2Bub, the input samples were incubated with the indicated antibodies and then added with protein A/G agarose beads (Thermo, 26159). For the ChIPs of Rad51-5FLAG, H2B-5FLAG, Brl1-5FLAG, and Ubp8-13Myc, the input samples were incubated with the FLAG agarose beads (Sigma, A2220) and c-Myc magnetic beads (MCE, HY-K0206) overnight at 4 °C. After extensive washing with lysis buffer, high salt buffer, and LiCl buffer, the immunoprecipitated chromatin was eluted and reverse crosslinked with 50 μl of TE containing 1% SDS at 65 °C overnight. After digestion with 100 μg/ml proteinase K (NEB, P8102) and 100 μg/ml RNase A (Thermo, EN0531), the immunoprecipitated and input DNA were purified using the MinElute PCR purification kit (Qiagen, 28004). 6 μl of diluted DNA templates, 10 μl of 2x SYBR Green PCR master mix (TaKaRa, RR420A), and 4 μl of primer sets (2 μM each) at indicated genomic positions were mixed and run on the BioRad and ABI StepOne Real-Time PCR instruments. The enrichment at the indicated locus was calculated as the percentage of immunoprecipitated DNA level relative to the input DNA level by the comparative Ct (2^−Ct^) method. For the HO induction experiments, we performed ChIP experiments before (t = 0 h) and after HO induction (t = 20 h), separately. Thus, we normalized the enrichment in the *htb1-G52D*/*P102L* mutants to that in the WT (set as 1) as fold change at the induced time points of 0 h and 20 h, separately. The primers are listed in Table S9.

### Immunoprecipitation (IP) assay

The immunoprecipitation assay was based on a previous publication (96). 100 OD_600_ cells were resuspended in IP lysis buffer (50 mM Tris-HCl (pH 8.0); 150 mM NaCl; 1 mM MgAc_2_; 1 mM EDTA; 0.5% NP-40; 10% Glycerol) with yeast protease inhibitors (Sangon, C510026) and the deubiquitinase inhibitor N-Ethylmaleimide (NEM) (Sigma, E1271). The cells were lysed with FastPrep (MP Biomedicals) bead-beating. The lysate was digested with Benzonase nuclease (Millipore, 70664) to release histones from the chromatin to the supernatant. The cleared supernatant as input was incubated with anti-FLAG M2 affinity gel (Sigma, A2220) for 2 h at 4 °C. Immunoprecipitated proteins were then washed 3 times with lysis buffer and eluted with 2x SDS-loading buffer for 10 min at 70 °C.

### Statistical analysis

Experiments were repeated two or more times with independent biological repeats. Data collection and statistical analysis were performed using Microsoft Office Excel and GraphPad Prism software. All data are expressed as mean values with indicated error bars and are statistically analyzed using Fisher’s exact test, Student’s *t* test, and ordinary one-way ANOVA with Dunnett’s multiple comparisons test. ns, ∗, ∗∗, ∗∗∗, and ∗∗∗∗ indicates no significance, *p* < 0.05, *p* < 0.01, *p* < 0.001, and *p* < 0.0001, respectively.

## Data availability

The RNA-seq data have been deposited to the NCBI Gene Expression Omnibus (GEO) under accession number GSE212629. All data that support this study are included in the article and its supporting information.

## Supporting information

This article contains supporting information.

## Conflict of interest

The authors declare that they have no known competing financial interests or personal relationships that could have appeared to influence the work reported in this paper.
