# Supplementary material for: H2B oncohistones cause homologous recombination defect and genomic instability through reducing H2B monoubiquitination in Schizosaccharomyces pombe
Source: J Biol Chem. 2024 May 7;300(6):107345. doi: 10.1016/j.jbc.2024.107345 (PMC11167522; doi:10.1016/j.jbc.2024.107345)
Supplement: Supporting Figure S13 [file mmc6.pdf]

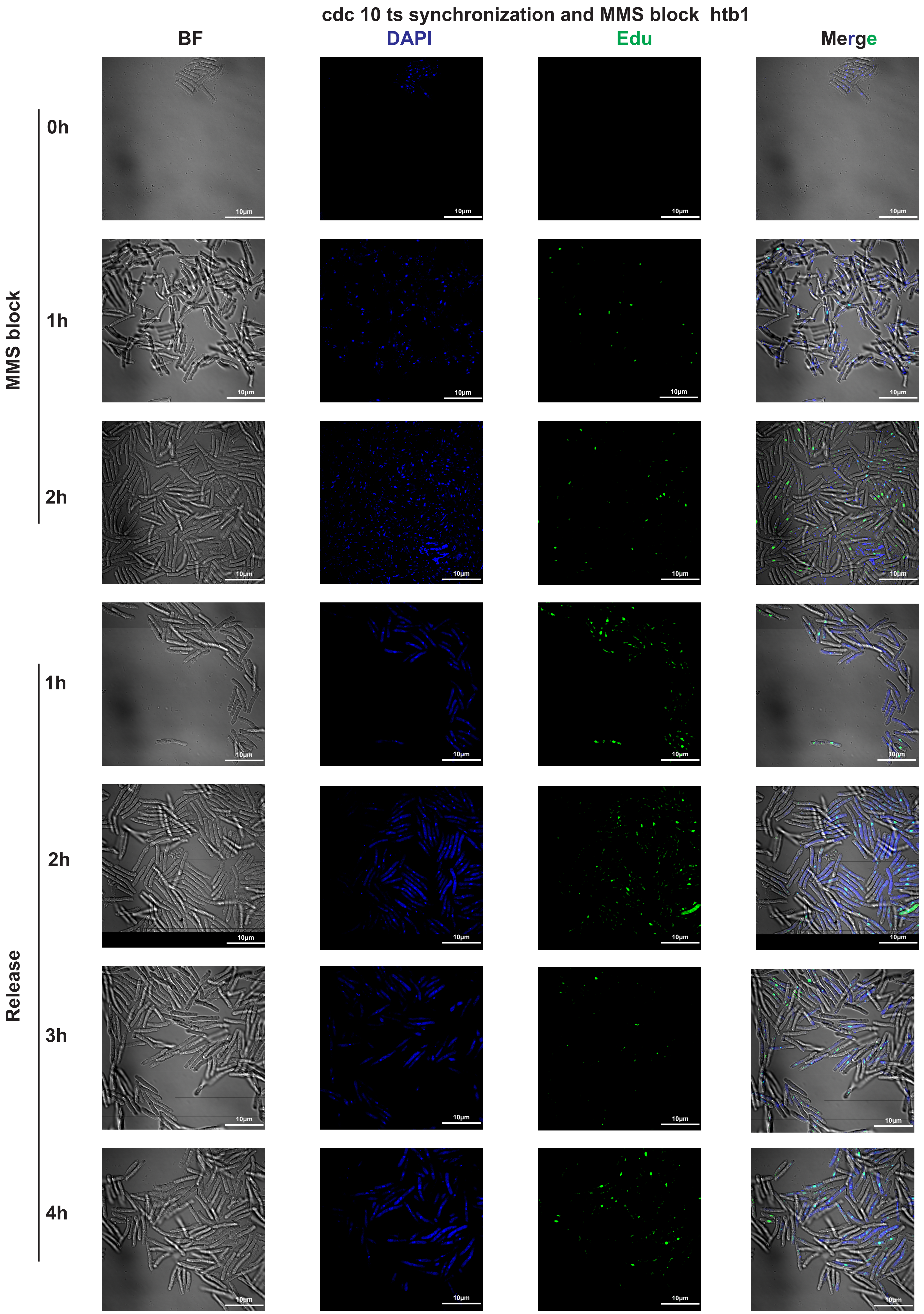
cdc 10 ts synchronization and MMS block htb1
BF
DAPI
Edu
Merge
MMS block
0h
1h
2h
Release
1h
2h
3h
4h
10μm

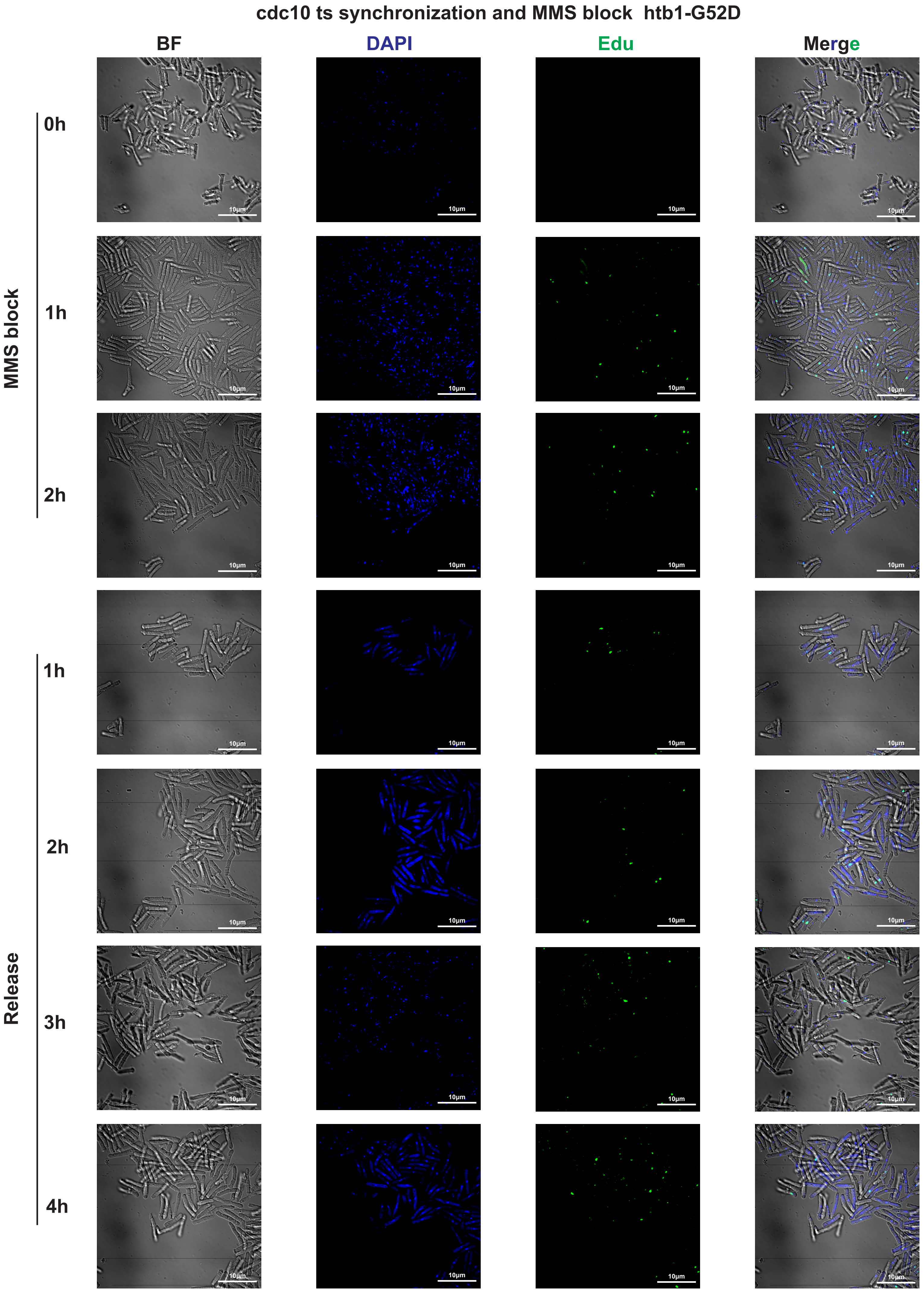
cdc10 ts synchronization and MMS block htb1-G52D
BF
DAPI
Edu
Merge
MMS block
0h
1h
2h
Release
1h
2h
3h
4h
10μm

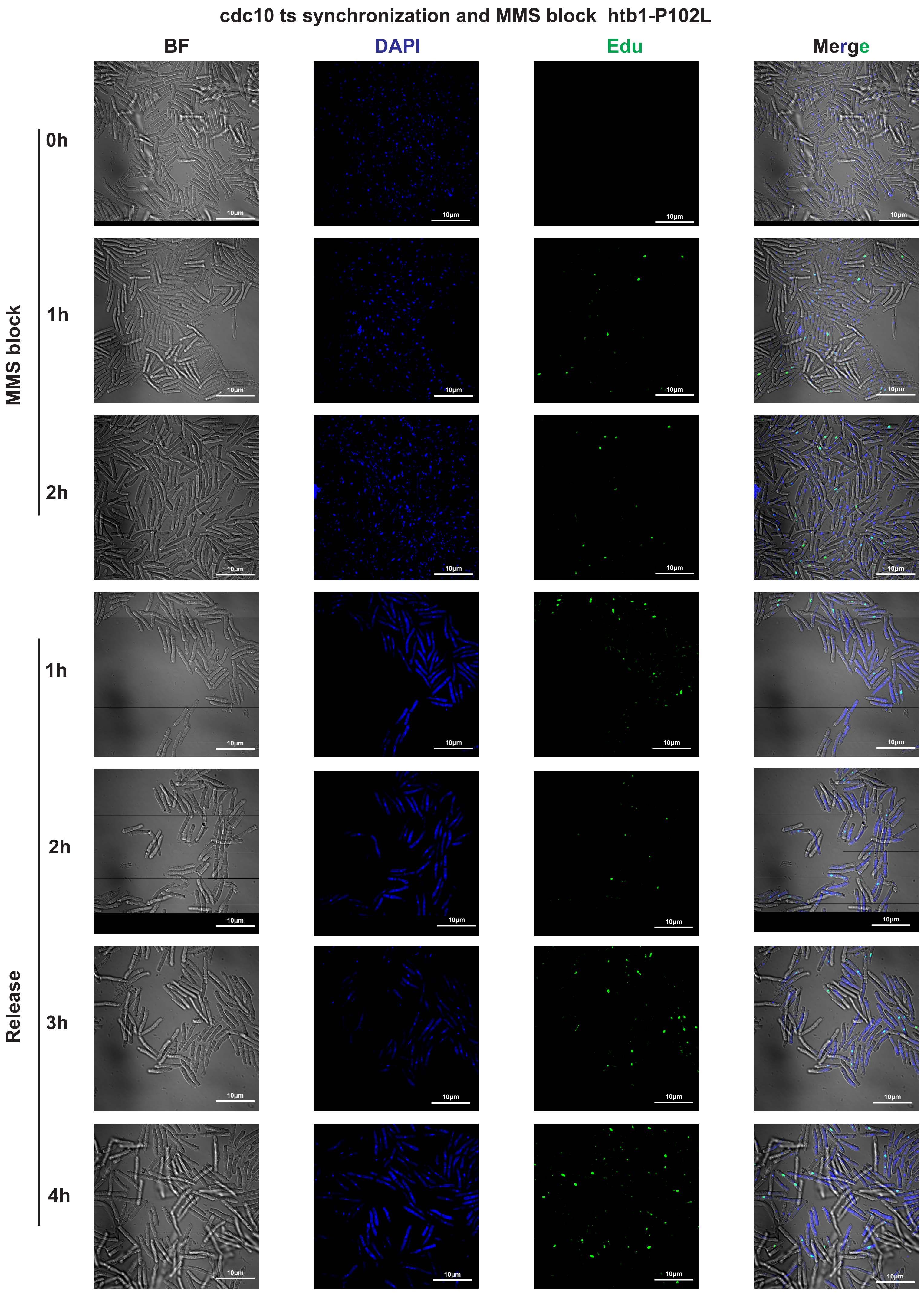
cdc10 ts synchronization and MMS block htb1-P102L
BF
DAPI
Edu
Merge
MMS block
0h
1h
2h
Release
1h
2h
3h
4h
10μm
