# Supplementary material for: H2B oncohistones cause homologous recombination defect and genomic instability through reducing H2B monoubiquitination in Schizosaccharomyces pombe
Source: J Biol Chem. 2024 May 7;300(6):107345. doi: 10.1016/j.jbc.2024.107345 (PMC11167522; doi:10.1016/j.jbc.2024.107345)
Supplement: Supporting Figure S14 [file mmc7.pdf]

Asynchronization and without MMS *htb1*

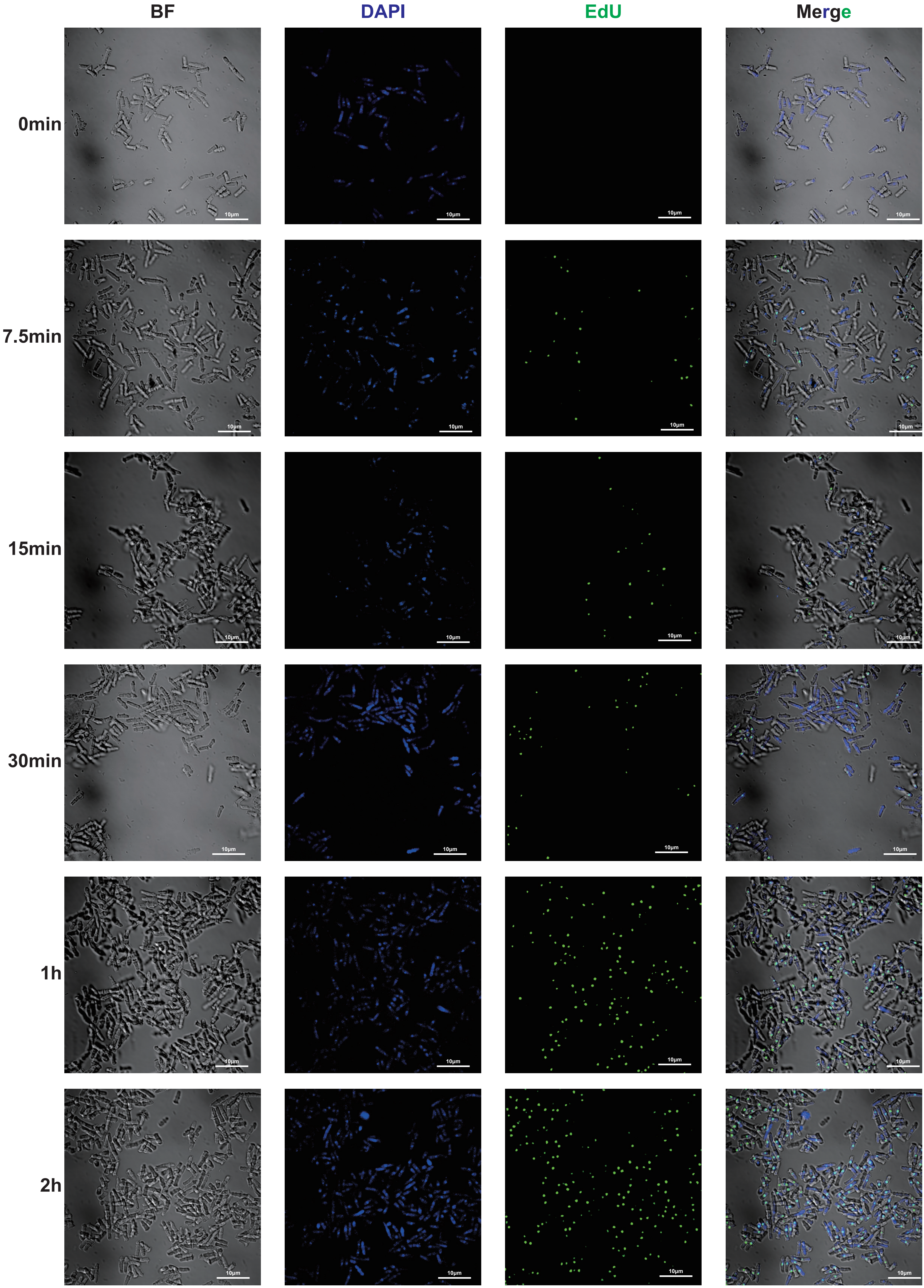

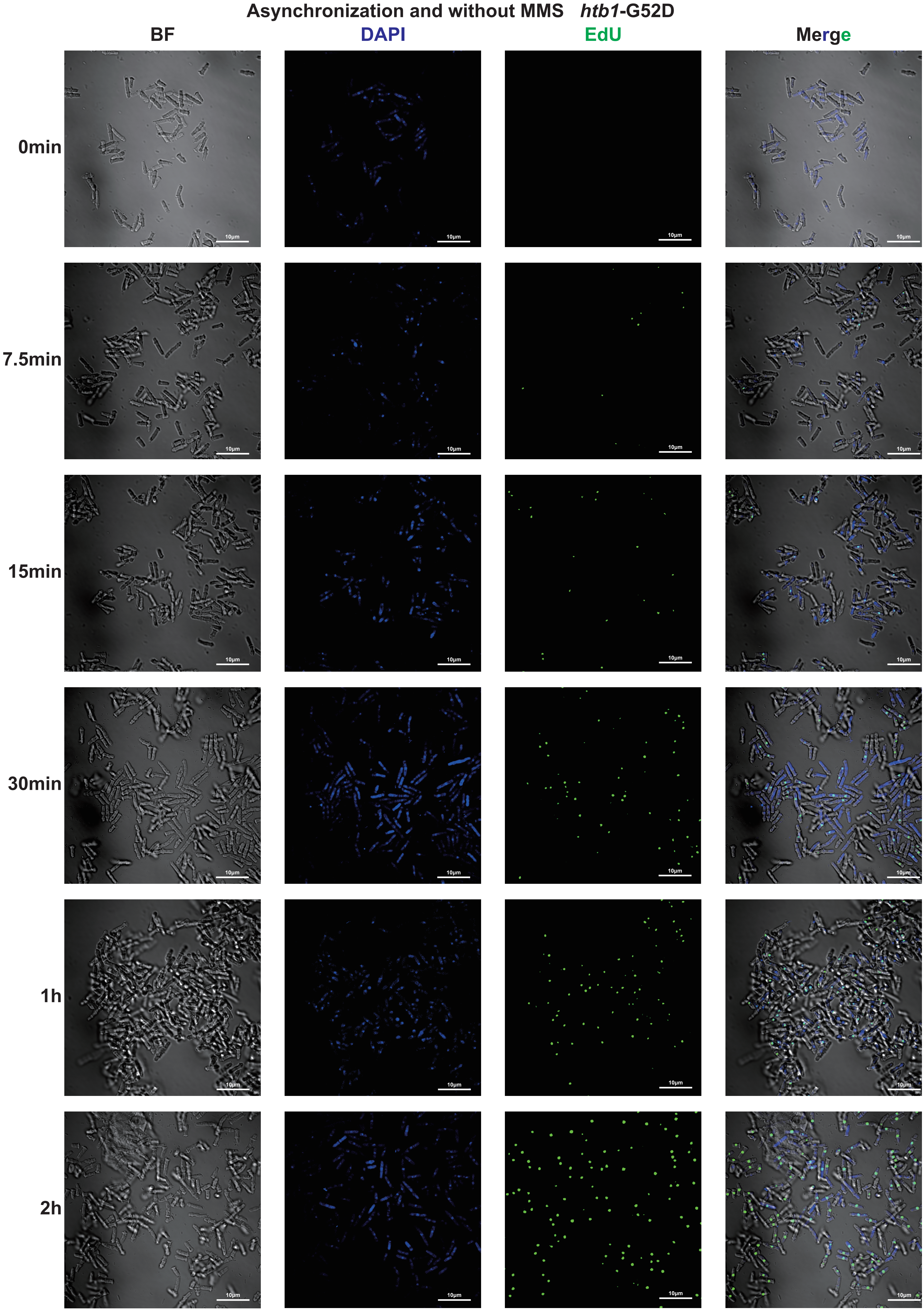
Asynchronization and without MMS *htb1*-G52D
BF
DAPI
EdU
Merge
0min
7.5min
15min
30min
1h
2h
10μm

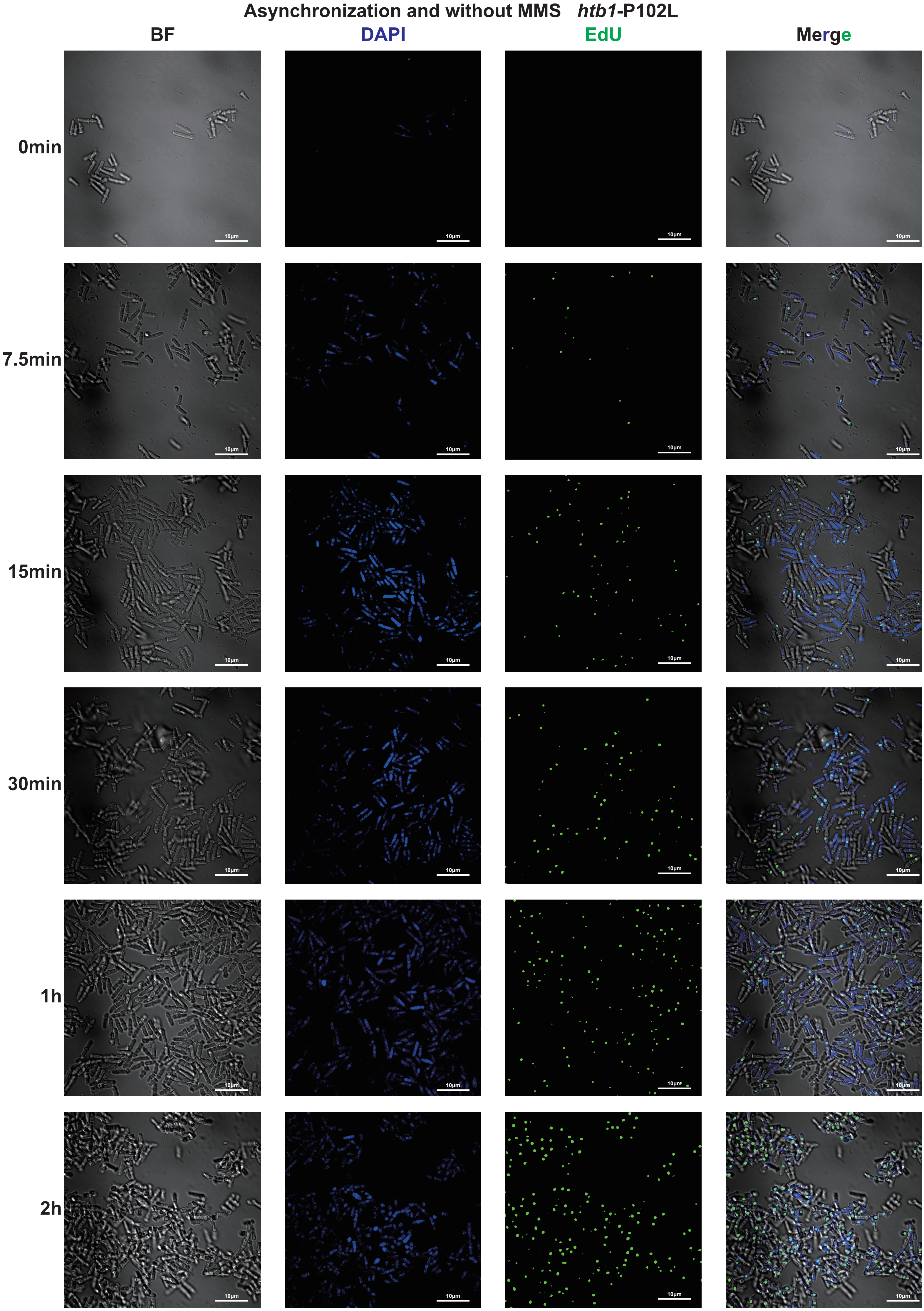

Asynchronization and without MMS htb1-P102L
BF
DAPI
EdU
Merge
0min
7.5min
15min
30min
1h
2h
10μm
