# Supplementary material for: H2B oncohistones cause homologous recombination defect and genomic instability through reducing H2B monoubiquitination in Schizosaccharomyces pombe
Source: J Biol Chem. 2024 May 7;300(6):107345. doi: 10.1016/j.jbc.2024.107345 (PMC11167522; doi:10.1016/j.jbc.2024.107345)
Supplement: Supporting Information [file mmc9.pdf]

Supporting Information

H2B oncohistones cause homologous recombination defects and genomic instability through reducing H2B monoubiquitination in *Schizosaccharomyces pombe*

Bingxin Qin[1,3], Guangchun Lu[1,3], Xuejin Chen[2], Chenhua Zheng[2], Huanteng Lin[2], Qi Liu[1], Jinjie Shang[1], and Gang Feng[1,2,*]

[1]Jiangsu Key Laboratory for Microbes and Functional Genomics, College of Life Sciences, Nanjing Normal University, Nanjing 210023, China
[2]School of Basic Medical Sciences, Fujian Medical University, Fuzhou 350122, China
[3]These authors contributed equally to this work

[*]Correspondence: fengg@njnu.edu.cn

A list of the materials

Figures of S1-S11

Figures of S12-S15 are separate pdf files

Tables of S1, S2, S3, S8, S9

Tables of S4, S5, S6, S7 are separate excel files

Figure S1

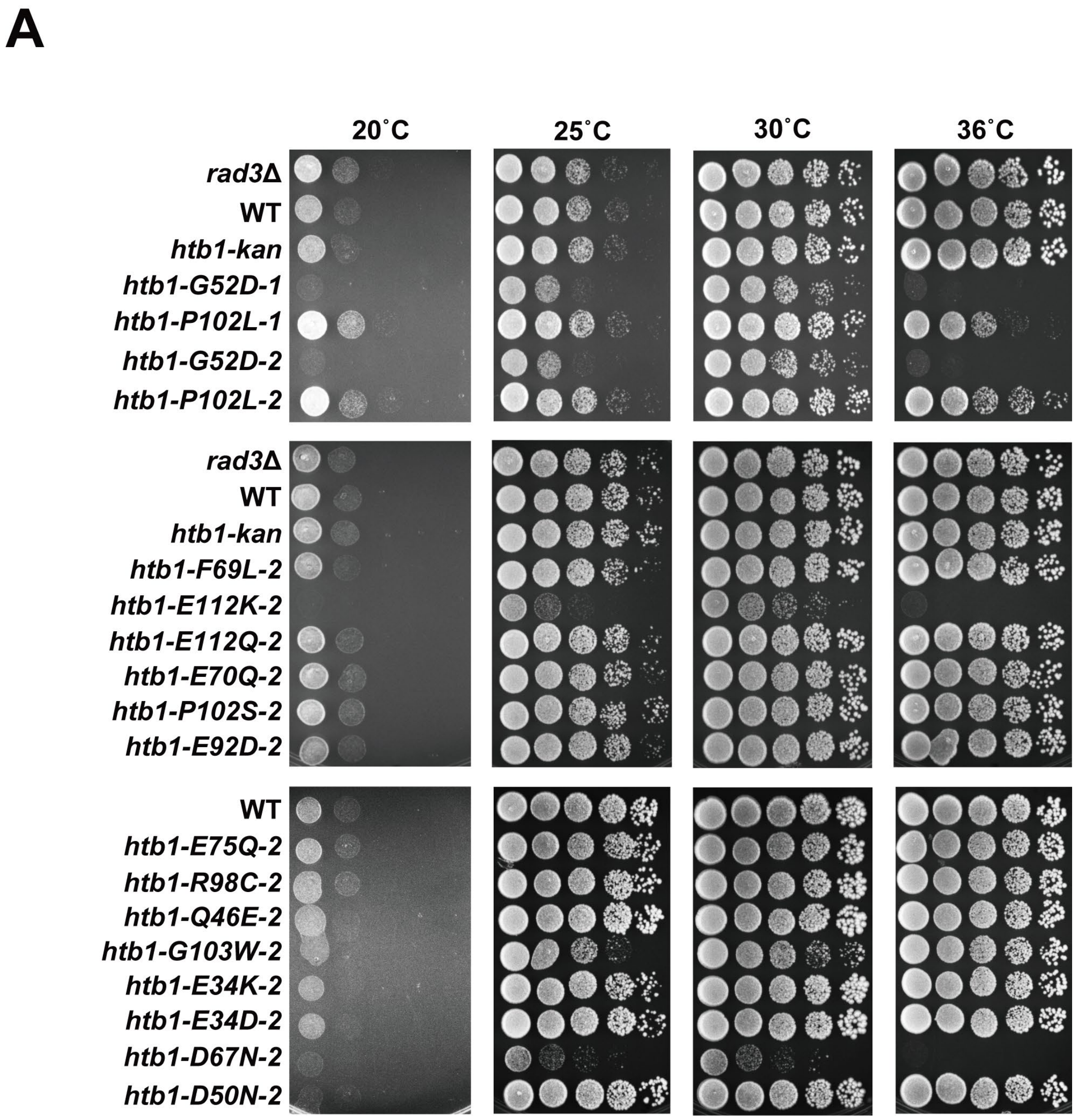

Figure S1 (related to Figure 1). *A*, the growth phenotypes of the second independent biological repeats of 16 H2B onco-mutants under indicated conditions. The images in the top row of the 25°C, 30°C, and 36°C are reuse of Figure 1*A*, additionally with 2nd repeats of *htb1-G52D/P102L*.

Figure S1

B

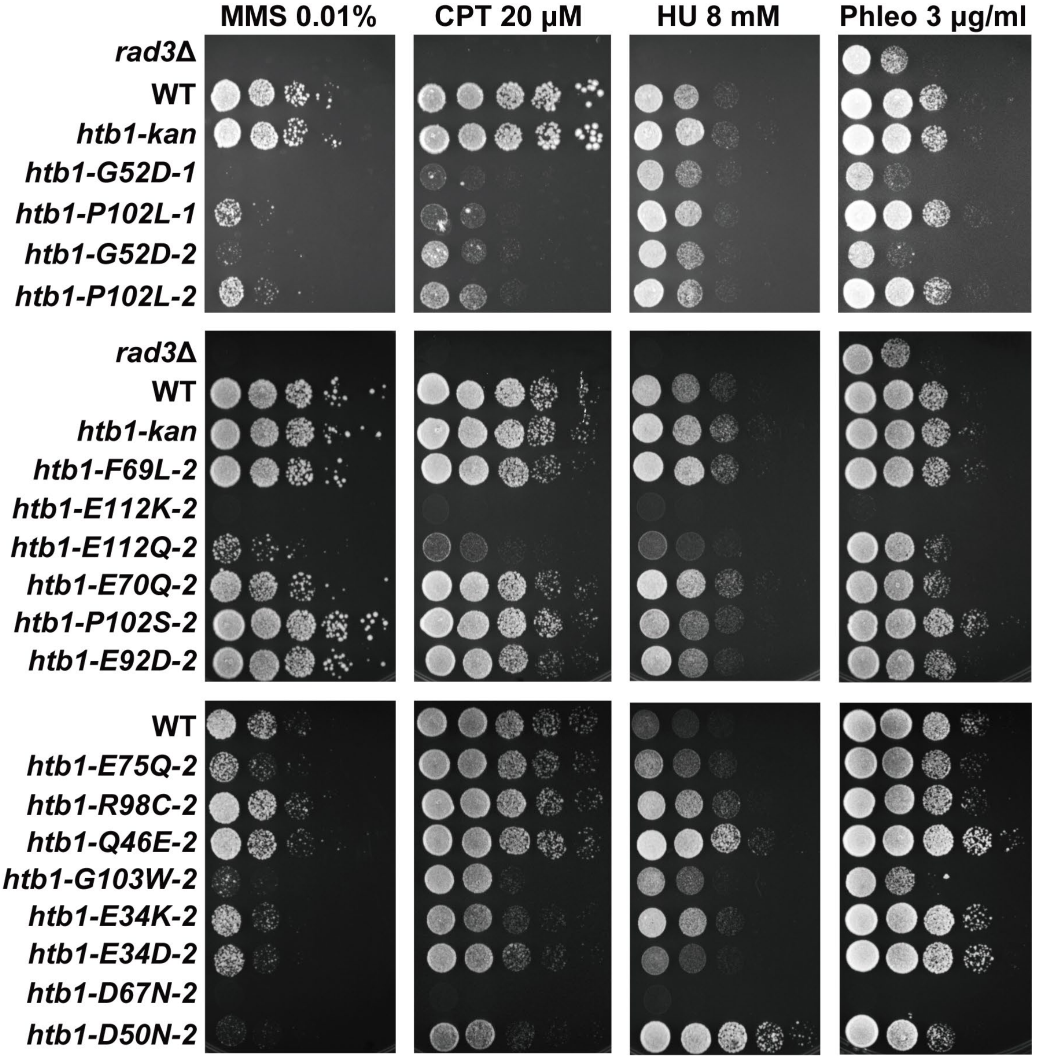

Figure S1 (related to Figure 1). *B*, the growth phenotypes of the second independent biological repeats of 16 H2B onco-mutants under indicated conditions. The images in the top row of the MMS, CPT, HU, and Phleo are reuse of Figure 1, *A and B*, additionally with 2nd repeats of *htb1-G52D/P102L*.

Figure S1

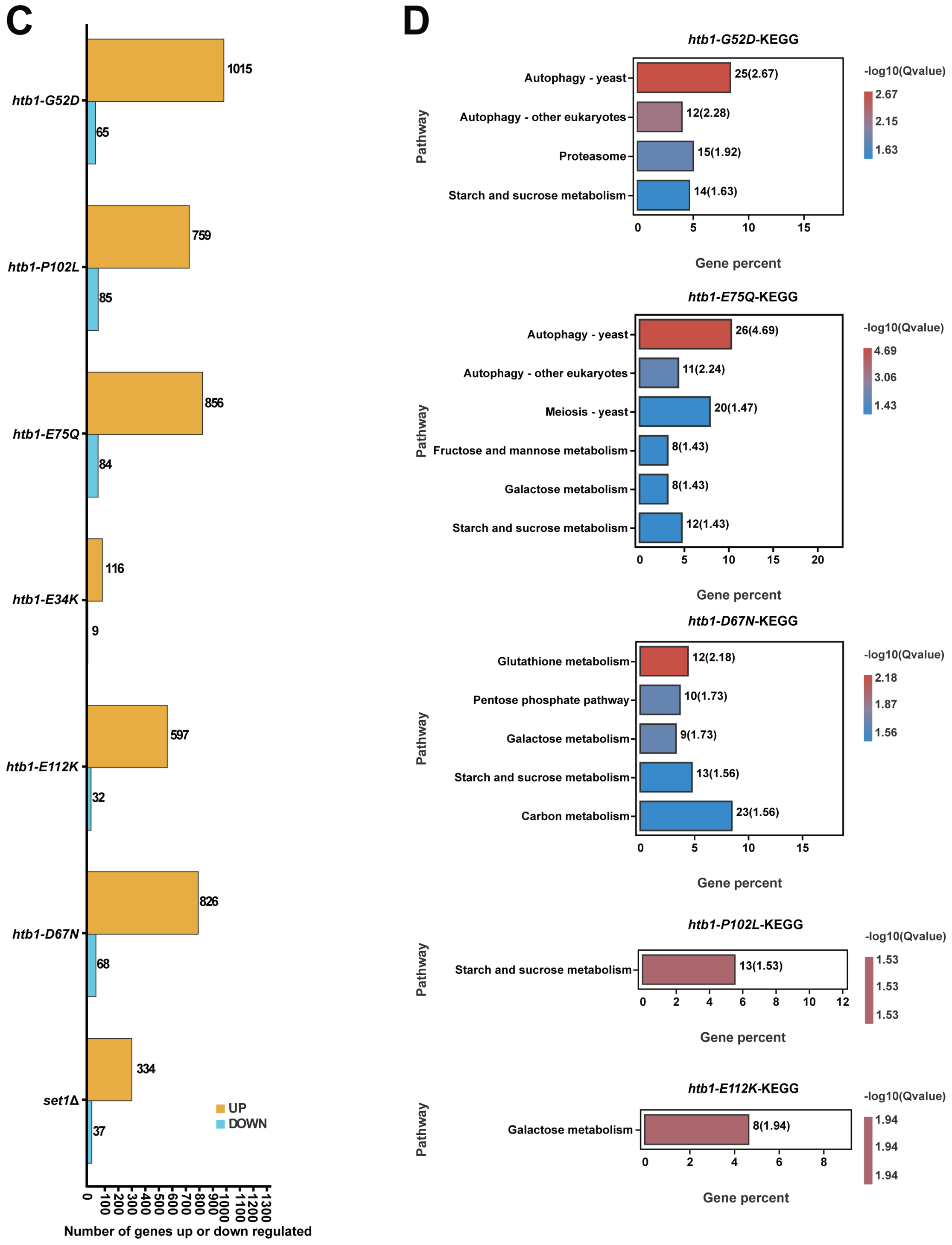

Figure S1 (related to Figure 1). *C*, the analysis of the differentially expressed genes (DEGs) of *htb1-G52D* (YGF277), *htb1-P102L* (YGF279), *htb1-E34K* (YGF275), *htb1-E75Q* (YGF317), *htb1-E112K* (YGF328), *htb1-D67N* (YGF324), and *set1Δ* (YGF285) compared with WT (TK8) cells. The number of DEGs is shown. *D*, top 10 significantly ($q<0.05$) enriched KEGG pathways in the indicated H2B onco-mutants. The number of enriched genes and -log10(q) value in each pathway are shown.

Figure S1

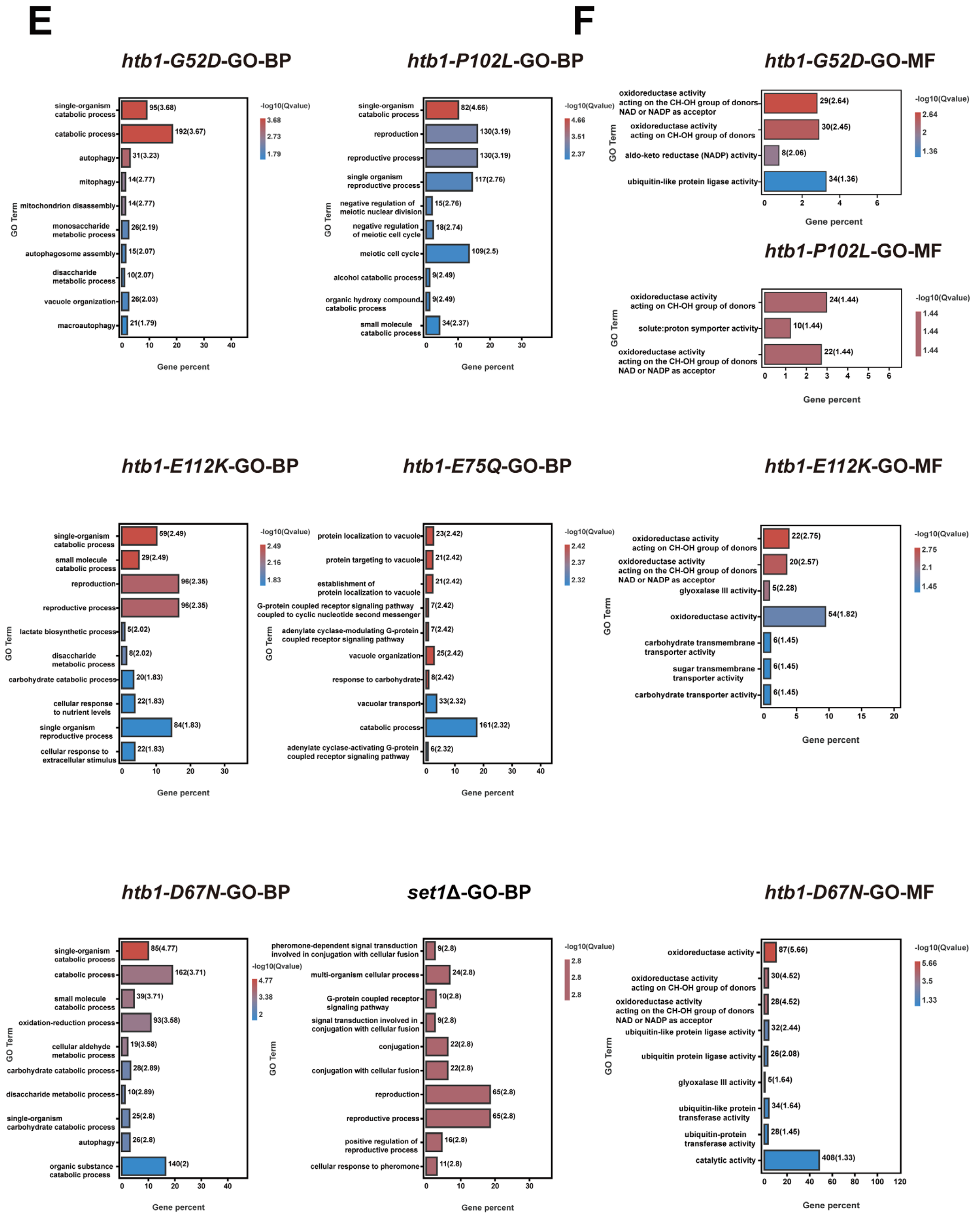

Figure S1 (related to Figure 1). *E*, top 10 significantly (q<0.05) enriched GO-BP pathways in the indicated H2B onco-mutants. The number of enriched genes and -log10(q) value in each GO term are shown. *F*, top 10 significantly (q<0.05) enriched GO-MF pathways in the indicated H2B onco-mutants. The number of enriched genes and -log10(q) value in each GO term are shown.

Figure S1

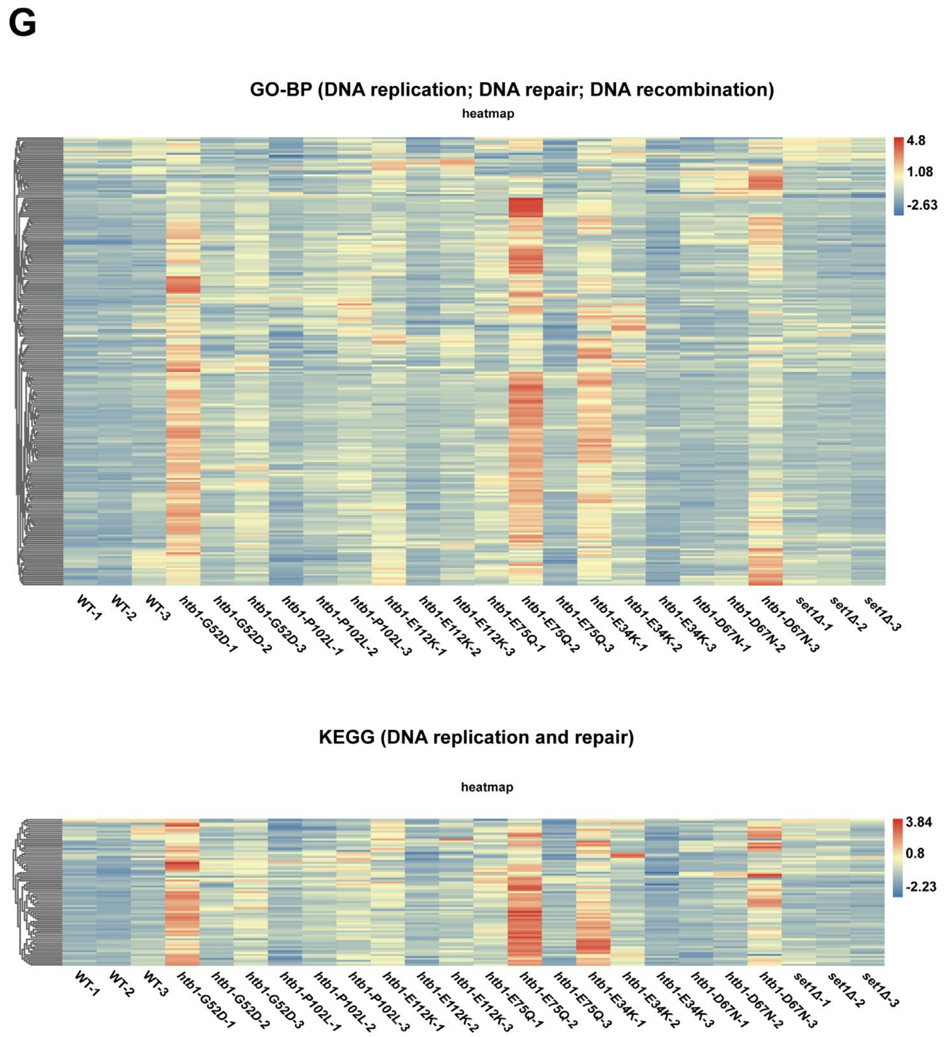

Figure S1 (related to Figure 1). *G*, *upper panel*, gene expression of GO-BP ontology 0006260 (DNA replication), 0006281 (DNA repair), and 0006310 (DNA recombination) are shown in the heatmap. *Lower panel*, gene expression of KEGG ontology of 03030 (DNA replication), 03410 (base excision repair), 03420 (nucleotide excision repair), 03430 (mismatch repair), 03440 (homologous recombination), and 03450 (non-homologous end-joining) are demonstrated in the heatmap.

Figure S2

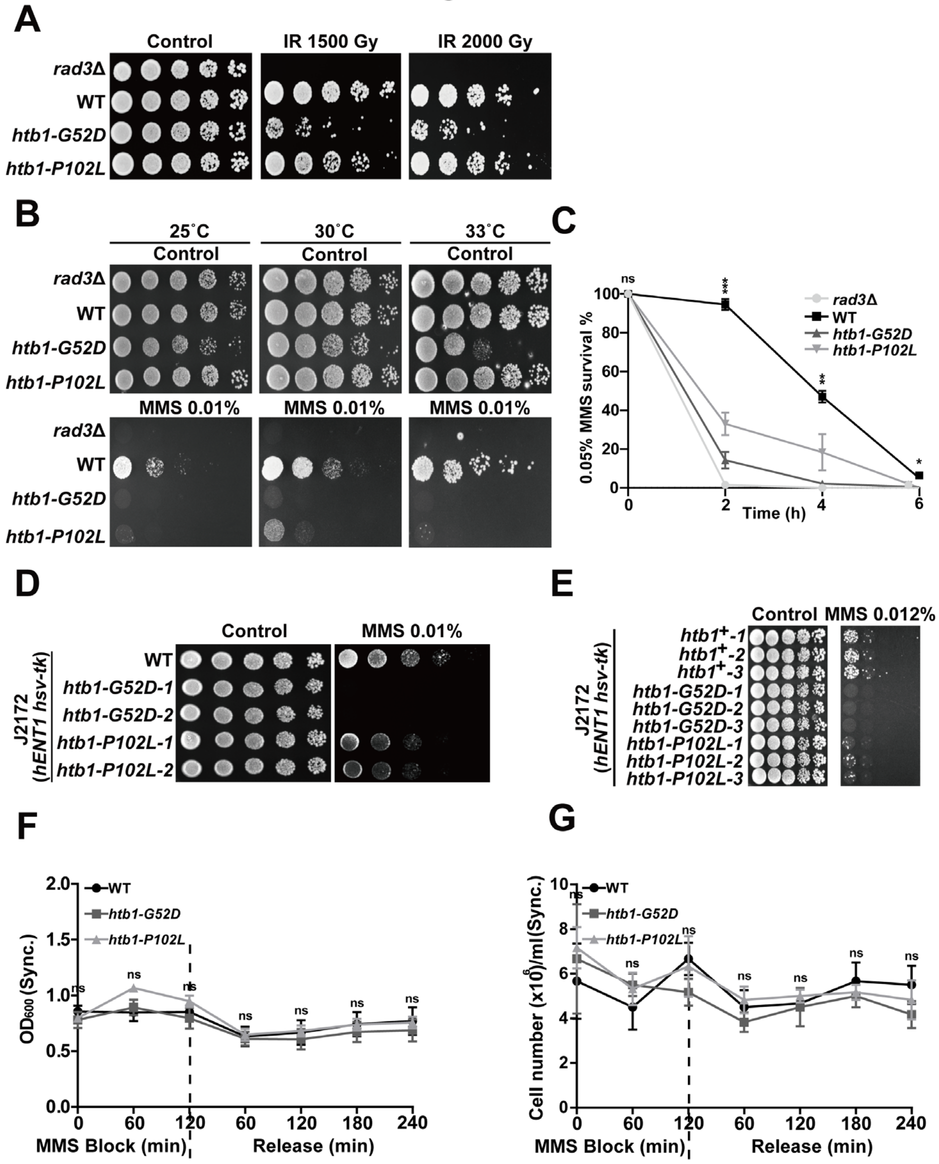

Figure S2 (related to Figure 2). *A*, the growth of the *htb1-G52D* and *htb1-P102L* mutant under the indicated intensity of IR. *B*, the growth of WT (TK8), *htb1-G52D* (YGF277), and *htb1-P102L* (YGF279) strains under 0.01% MMS at 25°C, 30°C, and 33°C. *C*, the survival percentage of *rad3*Δ (LD297) as a positive control, WT (TK8), *htb1-G52D* (YGF277), and *htb1-P102L* (YGF279) cells after 0.05% MMS treatment at the indicated timepoints. The survival percentage from two independent biological repeats is averaged and SEM is shown as the error bar. One-way ANOVA is statistically performed at each timepoint. *D* and *E*, the growth of EdU-incorporating strains of WT (J2172), *htb1-G52D* (YGF281), and *htb1-P102L* (YGF282) under 0.01% (*D*) and 0.012% (*E*) MMS. *F* and *G*, EdU-incorporating strains of WT (J2172), *htb1-G52D* (YGF281), and *htb1-P102L* (YGF282) were synchronized into early S phase by 12 mM HU treatment, and then labeled with EdU at the indicated timepoints under 0.05% MMS block and release conditions. MMS block is shown on the left of dotted line, and then release into normal condition is shown on the right. The numbers of OD_{600} (*F*) and cell concentrations (*G*) from three independent biological repeats are averaged and error bars represent SEM. One-way ANOVA is statistically performed at each timepoint for multiple comparisons.

Figure S3

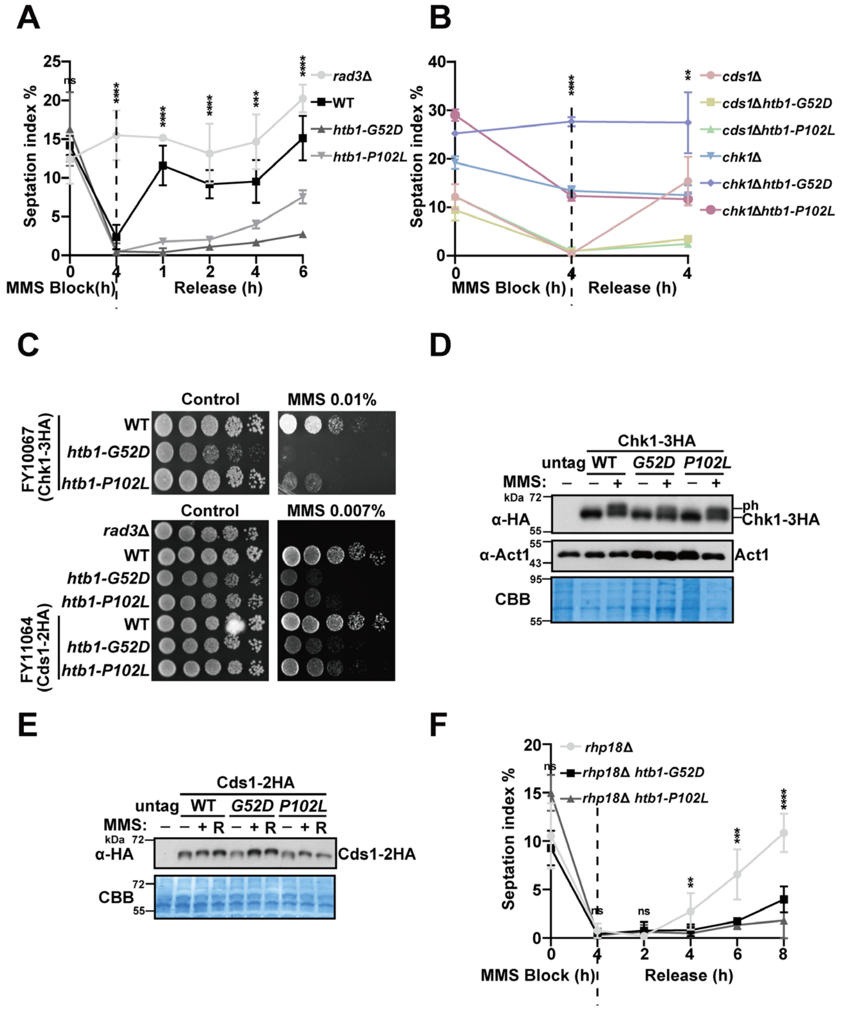

Figure S3 (related to Figure 3). *A*, the septation index of WT (TK8), *htb1-G52D* (YGF277), *htb1-P102L* (YGF279) and *rad3*Δ (LD297) under 0.05% MMS block and release at the indicated time point. The septation percentage of each sample is averaged from two independent biological repeats. Error bars reflect SDs. One-way ANOVA is statistically performed at each timepoint. *B*, the septation index of *cds1*Δ (Ets13), *cds1*Δ *htb1-G52D* (YGF287), *cds1*Δ *htb1-P102L* (YGF288), *chk1*Δ (Ets12), *chk1*Δ *htb1-G52D* (YGF291) and *chk1*Δ *htb1-P102L* (YGF292) under 0.05% MMS block (on the left of dotted line) and recovery (on the right of dotted line). The septation percentage of each sample is averaged from two independent biological repeats. Error bars represent SDs. One-way ANOVA is statistically performed at each timepoint. *C*, *upper panel*, the MMS sensitivity of Chk1-3HA (FY10067), Chk1-3HA *htb1-G52D* (YGF289), and Chk1-3HA *htb1-P102L* (YGF290) strains. *Lower panel*, the MMS sensitivity of Cds1-2HA (FY11064), Cds1-2HA *htb1-G52D* (YGF303), and Cds1-2HA *htb1-P102L* (YGF304). *D*, immunoblots of Chk1-3HA activation after treatment with 0.05% MMS for 2 h. Phosphorylated Chk1-3HA is labeled as ph. *E*, the immunoblot of Cds1-2HA activation after 2 h of 0.05% MMS block and 1 h of release (indicated as R). *F*, the septation index of *rhp18*Δ (FY20976), *rhp18*Δ *htb1-G52D* (YGF299) and *rhp18*Δ *htb1-P102L* (YGF300) under 0.05% MMS block and release at the indicated timepoint. The septation percentage of each sample is averaged from three independent biological repeats. Error bars reflect SDs. One-way ANOVA is statistically performed at each timepoint.

Figure S4

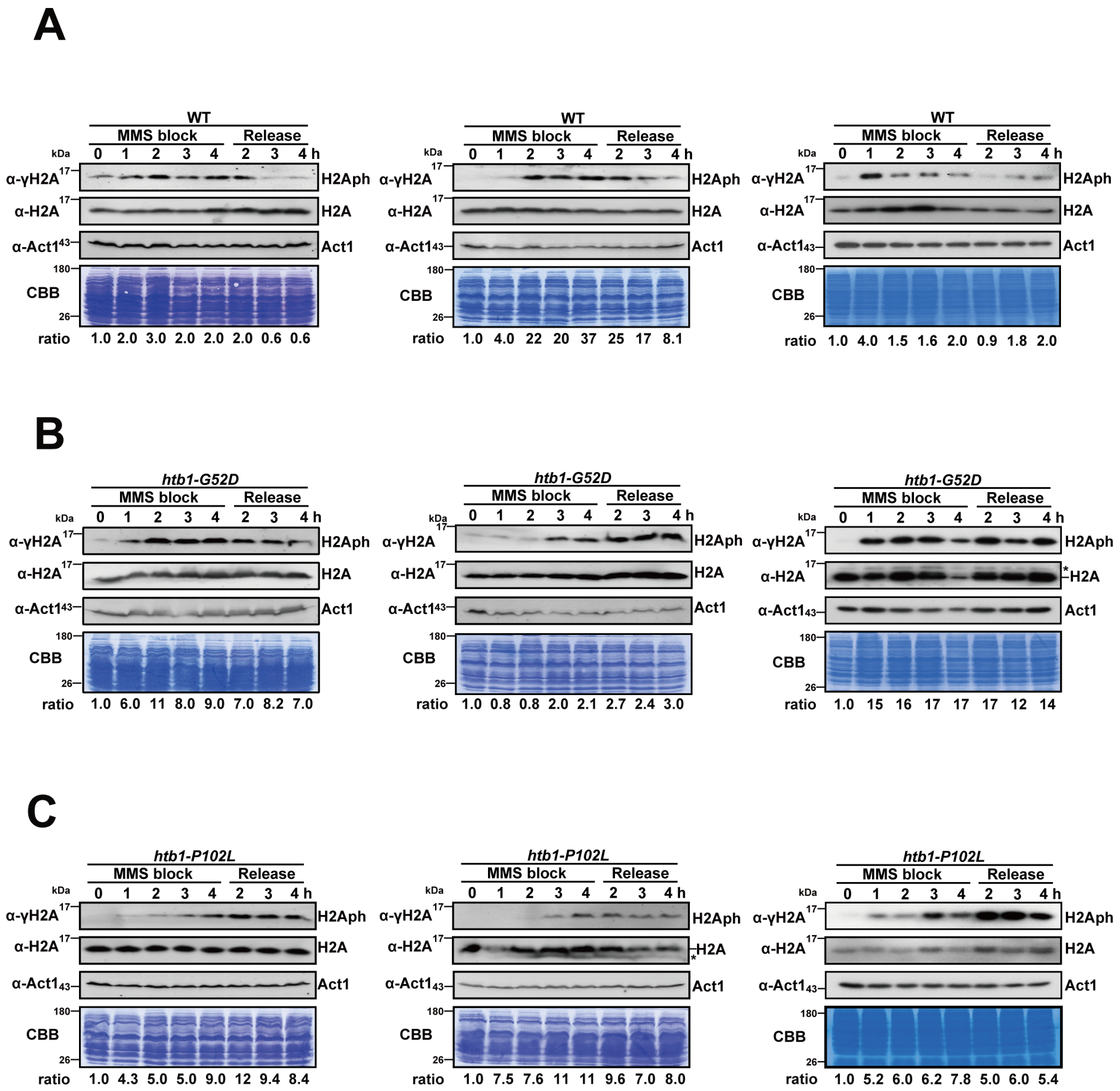

Figure S4 (related to Figure 4). The immunoblots of γH2A and H2A abundance during MMS block and release at the indicated timepoints in another three independent biological repeats of WT (TK8) (*A*), *htb1-G52D* (YGF277) (*B*), and *htb1-P102L* (YGF279) (*C*) cells. The γH2A signal normalized to H2A is used to calculate the ratio of the indicated timepoints to 0 h.

Figure S4

D

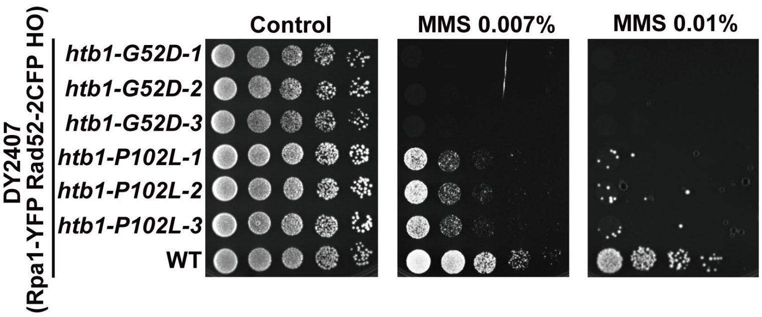

E

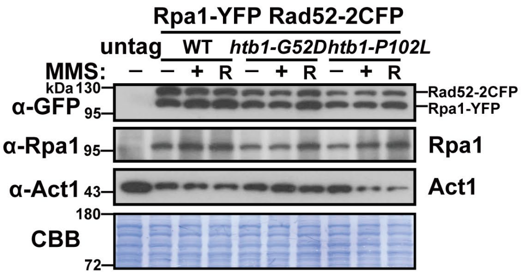

Figure S4 (related to Figure 4). *D*, MMS sensitivities of WT (DY2407), *htb1-G52D* (YGF276), and *htb1-P102L* (YGF278) strains with Rpa1-YFP and Rad52-2CFP. *E*, immunoblots of Rpa1-YFP and Rad52-2CFP with antibodies against GFP and Rpa1 in WT (DY2407), *htb1-G52D* (YGF276), and *htb1-P102L* (YGF278) cell extracts under MMS block and release.

Figure S5

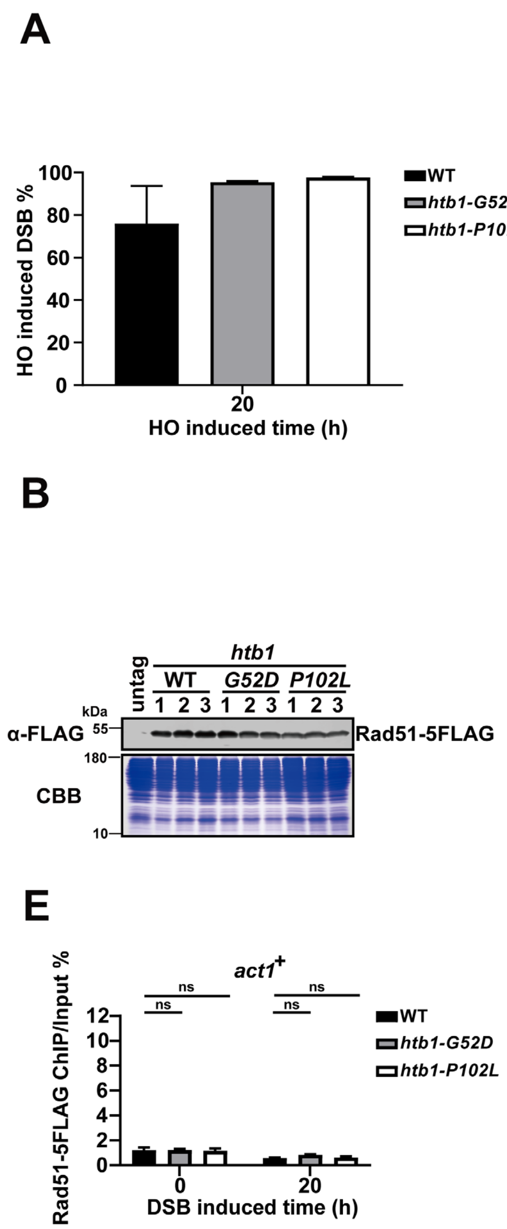

Figure S5 (related to Figure 5). *A*, DSB percentages after HO induction for 20 h in WT (DY2407), *htb1-G52D* (YGF276), and *htb1-P102L* (YGF278) strains. *B*, the immunoblots of Rad51-5FLAG in WT (YGF466), *htb1-G52D* (YGF462), and *htb1-P102L* (YGF463) strains. *C-D*, the ChIP-qPCR analysis of Rad51-5FLAG enrichment in untag cells (DY49) and WT (YGF466) at 35 bp, 3.1 kb, 9.4 kb, 20 kb adjacent to the HO-induced DSB site and *act1*$^+$ after HO-induced 0 h (*C*) and 20 h (*D*). The data from three independent biological repeats are averaged. Error bars represent SEMs. Student's t-test is used for comparing WT with untagged cells. *E*, the ChIP-qPCR analysis of Rad51-5FLAG enrichment in WT (YGF466), *htb1-G52D* (YGF462) and *htb1-P102L* (YGF463) at *act1*$^+$ after HO-induced 0 h and 20 h. The data from three independent biological repeats are averaged. Error bars represent SEMs. One-way ANOVA is used for multiple comparisons between the indicated samples and WT at HO-induced 0 h and 20 h.

Figure S6

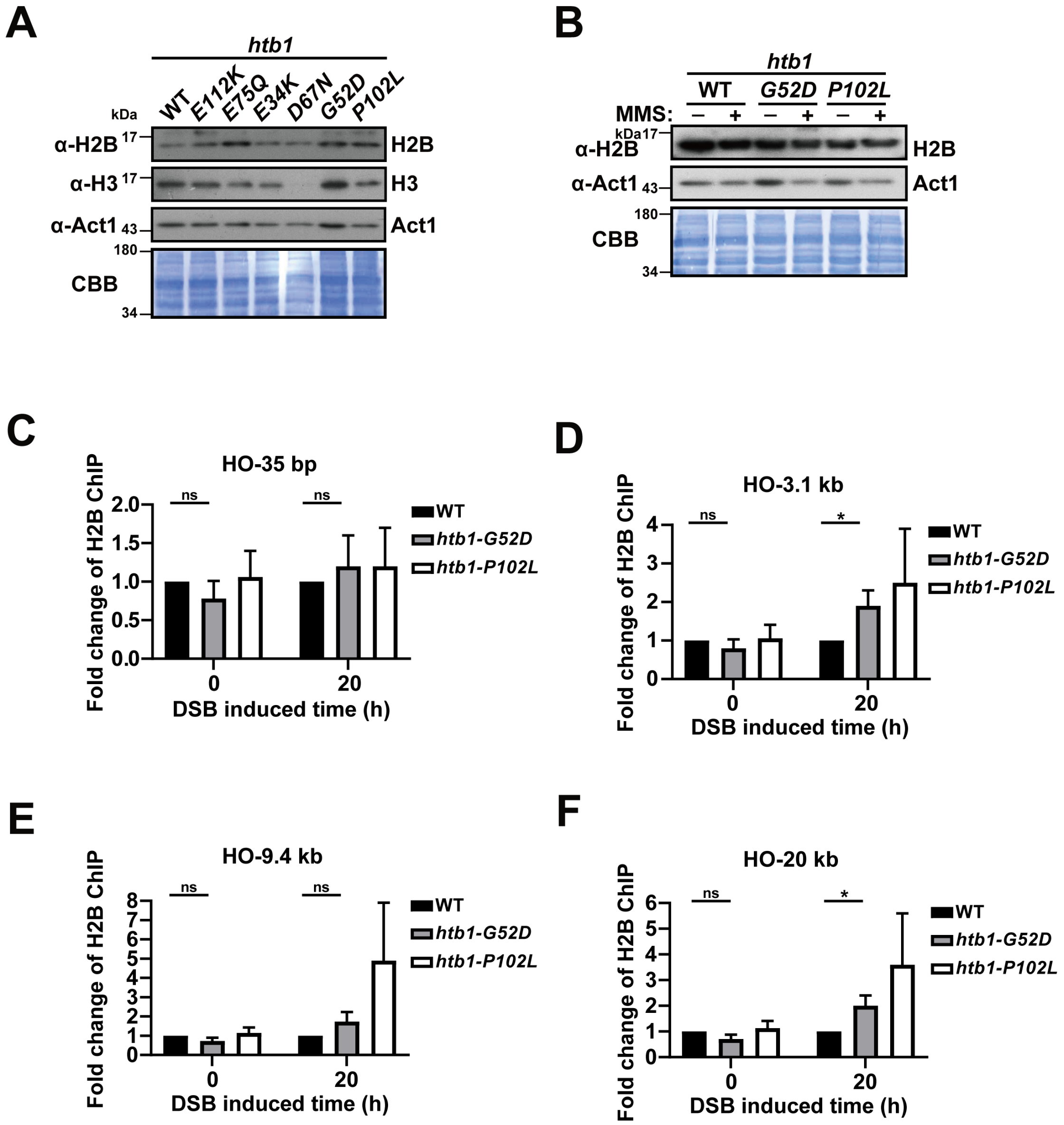

Figure S6 (related to Figure 6). *A*, the immunoblots of H2B levels in WT (TK8), *htb1-E112K* (YGF328), *htb1-E75Q* (YGF317), *htb1-E34K* (YGF275), *htb1-D67N* (YGF324), *htb1-G52D* (YGF277), and *htb1-P102L* (YGF279) strains. *B*, the immunoblots of H2B abundance in WT (TK8), *htb1-G52D* (YGF277), and *htb1-P102L* (YGF279) strains before and after 0.05% MMS treatment. *C-F*, the ChIP-qPCR analysis of H2B enrichment at 35 bp (*C*), 3.1 kb (*D*), 9.4 kb (*E*), and 20 kb (*F*) adjacent to the HO-induced DSB site. The data from two or three independent biological repeats are averaged. Error bars represent SEMs. Student's t-test is used for comparing *htb1-G52D* with WT at HO-induced 0 h and 20 h.

Figure S6

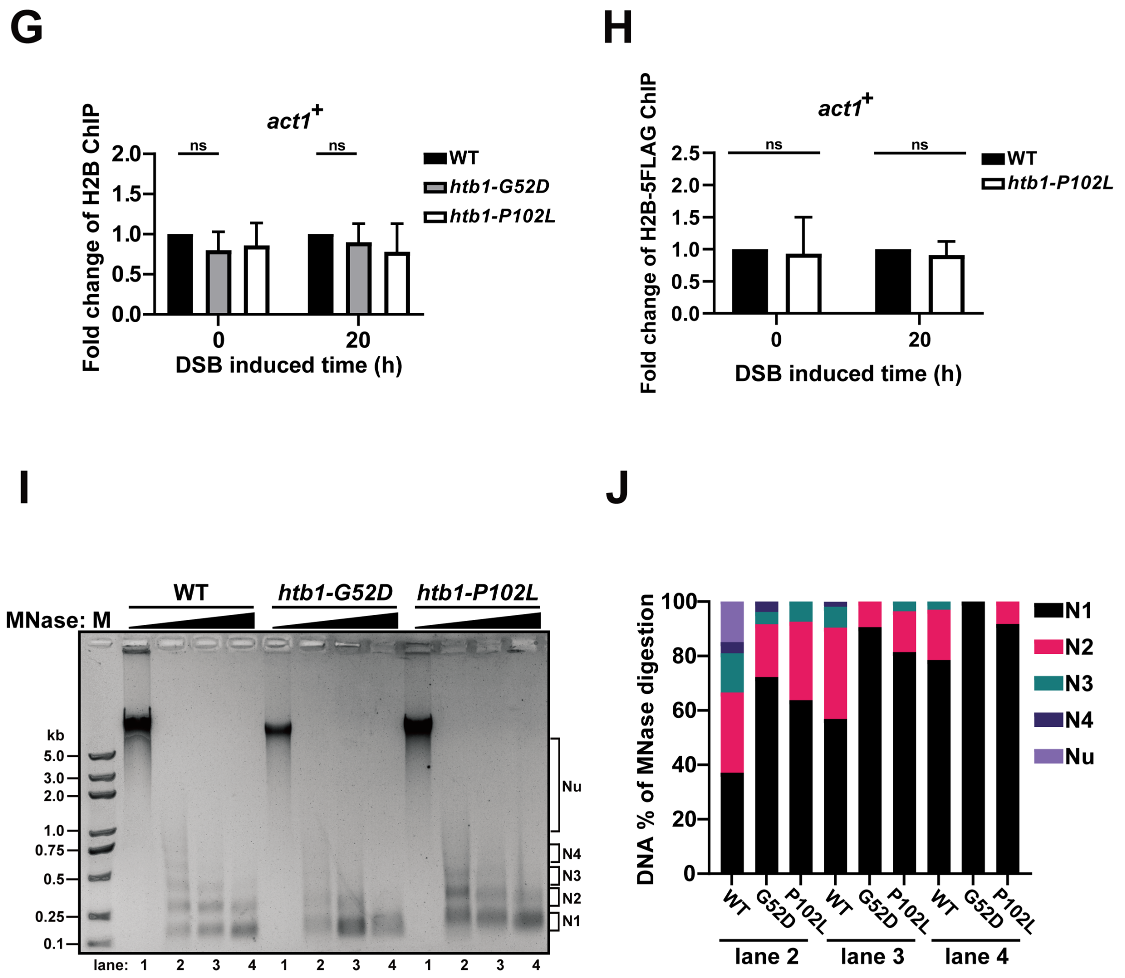

Figure S6 (related to Figure 6). *G*, after HO-induced 0 h or 20 h, the fold change of H2B enrichment in the *htb1-G52D* (YGF276) and *htb1-P102L* (YGF278) relative to WT (DY2407) at *act1*$^{+}$ gene body is shown. *H*, the ChIP-qPCR analysis of H2B-5FLAG enrichment at *act1*$^{+}$ gene body after HO-induced 0 h and 20 h. The fold change of H2B-5FLAG enrichment in the *htb1-P102L* (YGF492) relative to WT (DY49) is shown. The data from two or three independent biological repeats are averaged. Error bars represent SEMs. Student's t-test is used for comparing *htb1-P102L* with WT at HO-induced 0 h or 20 h. *I* and *J*, MNase assay was performed in second independent repeat of WT (TK8), *htb1-G52D* (YGF277) and *htb1-P102L* (YGF279) strains. *I*, the DNA gel after MNase digestion. N indicates the nucleosome DNA band; Nu indicates upper nucleosome DNA bands. *J*, quantification of nucleosomal DNA bands in lanes 2-4.

Figure S7

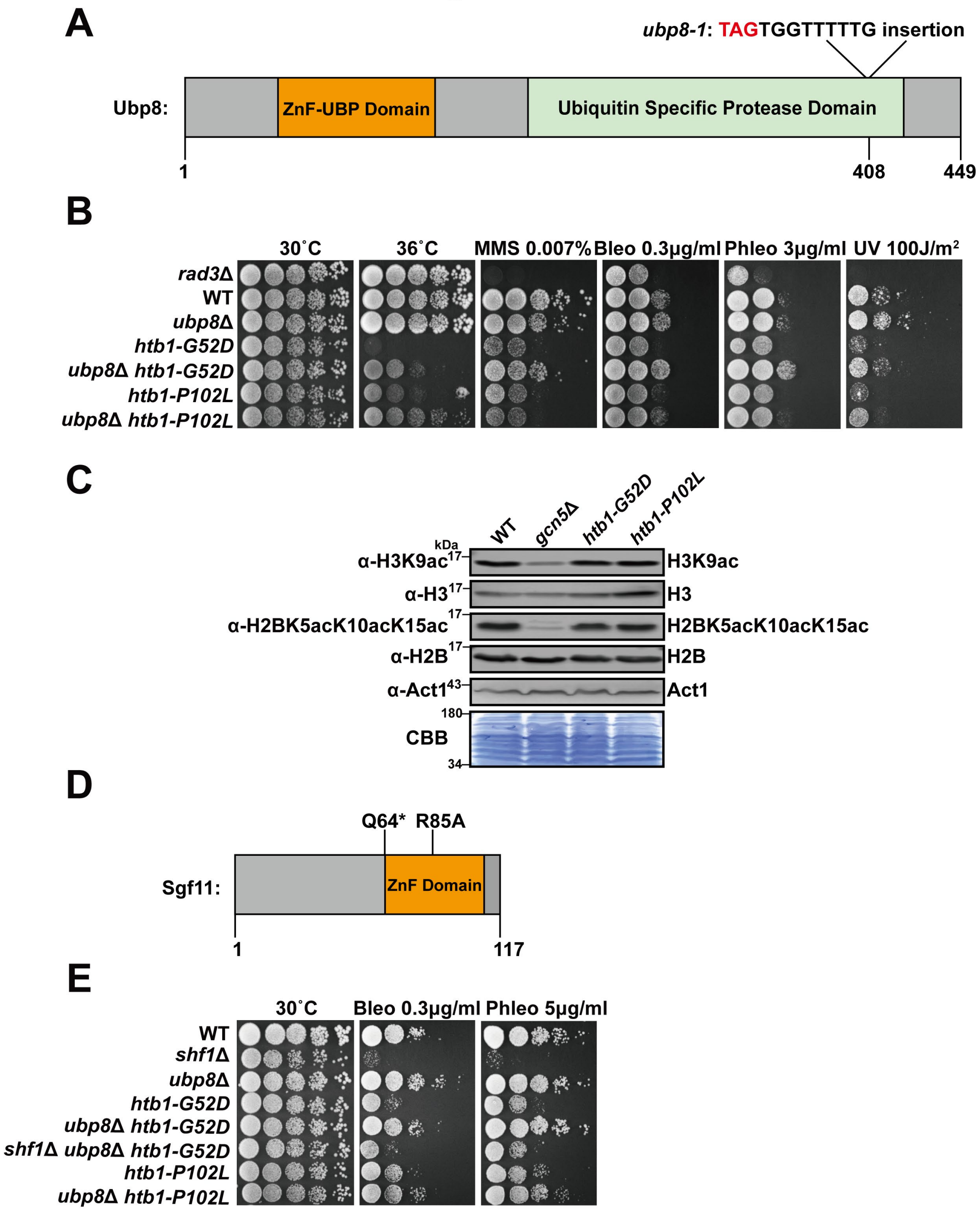

Figure S7 (related to Figure 7). *A*, the diagram of the *ubp8-1* mutation. *B*, the growth of the second independent biological repeats of *ubp8*Δ (YGF415), *htb1-G52D* (YGF277), *ubp8*Δ *htb1-G52D* (YGF416), *htb1-P102L* (YGF279), and *ubp8*Δ *htb1-P102L* (YGF417) strains in the presence of 36°C, MMS, bleomycin, phleomycin, and UV. *C*, the immunoblots of levels of H3K9ac and H2BK5acK10acK15ac in *gcn5*Δ (BN1), *htb1-G52D* (YGF277), and *htb1-P102L* (YGF279) cells. The specificity of the antibody against H2BK5acK10acK15ac was verified (our unpublished data). *D*, the diagram of the *sgf11-Q64** and *sgf11-R85A* mutations. * indicates the stop codon. *E*, the growth of the second independent biological repeat of *shf1*Δ (YGF431), *ubp8*Δ (YGF415), *htb1-G52D* (YGF277), *ubp8*Δ *htb1-G52D* (YGF416), *shf1*Δ *ubp8*Δ *htb1-G52D* (YGF432), *htb1-P102L* (YGF279), and *ubp8*Δ *htb1-P102L* (YGF417) strains in the presence of 30°C, bleomycin, and phleomycin.

Figure S8

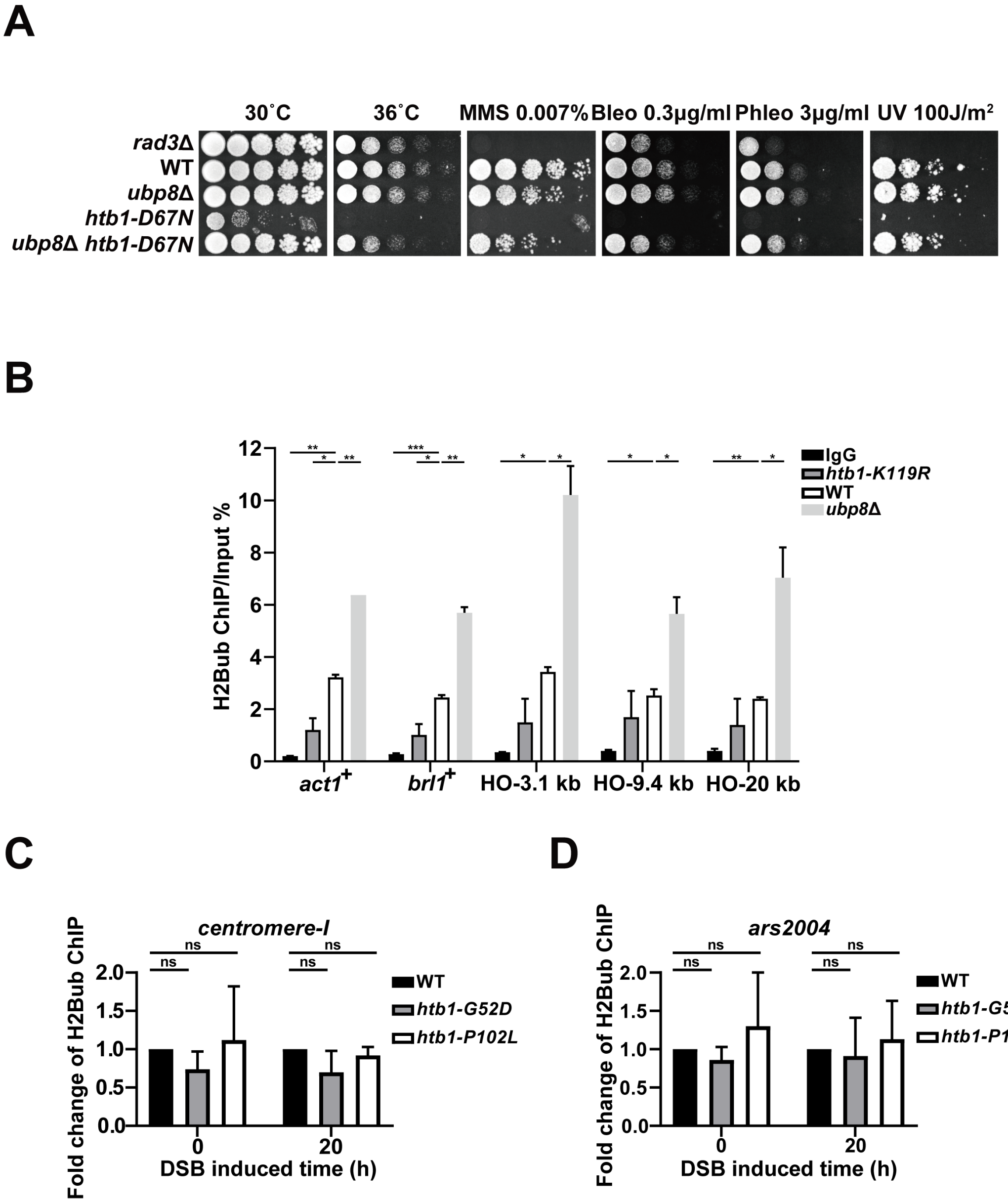

Figure S8 (related to Figure 8). *A*, the growth of *ubp8*Δ (YGF415), *htb1-D67N* (YGF324), and *ubp8*Δ *htb1-D67N* (YGF443) cells in the presence of 36°C, MMS, bleomycin, phleomycin, and UV. *B*, the ChIP-qPCR analysis of H2Bub enrichment at the indicated genomic positions. The H2Bub enrichments as the percentages in IP DNA relative to input DNA from the IgG mock, WT (DY2407), *htb1-K119R* (YGF226), and *ubp8*Δ (YGF415) are shown. The data from two or three independent biological repeats are averaged. Error bars represent SEMs. One-way ANOVA is used for multiple comparisons between the indicated samples and WT. *C* and *D*, the ChIP-qPCR analysis of H2Bub enrichment at *centromere-I* (*C*) and *ars2004* (*D*) after HO-induced 0 h or 20 h. The fold change of H2Bub enrichment in the *htb1-G52D* (YGF276) and *htb1-P102L* (YGF278) relative to WT (DY2407) is shown. The data from two or three independent biological repeats are averaged. Error bars represent SEMs. One-way ANOVA is used for multiple comparisons between the indicated samples and WT at HO-induced 0 h or 20 h.

Figure S9

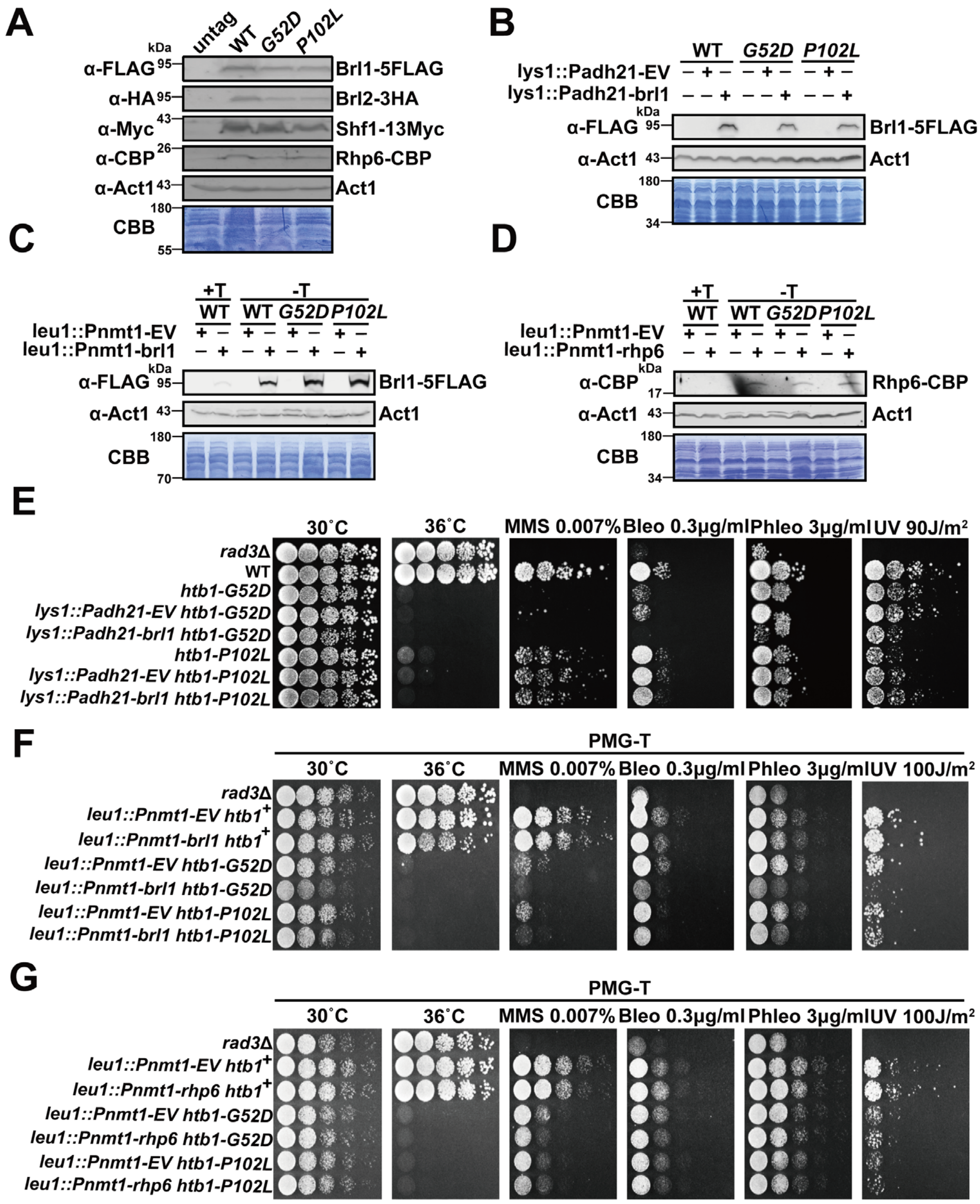

Figure S9 (related to Figure 9). *A*, the immunoblots of Brl1-5FLAG, Brl2-3HA, Shf1-13myc, and Rhp6-CBP in *htb1⁺* (YGF479), *htb1-G52D* (YGF496), and *htb1-P102L* (YGF497) cells. *B*, the immunoblots of overexpressed Brl1-5FLAG from *adh21* promoter in *htb1⁺*, *htb1-G52D* (YGF426), and *htb1-P102L* (YGF427) cells. *C*, the immunoblots of overexpressed Brl1-5FLAG from *nmt1* promoter in *htb1⁺* (YGF471), *htb1-G52D* (YGF474), and *htb1-P102L* (YGF477) cells. *D*, the immunoblots of overexpressed Rhp6-CBP from *nmt1* promoter in *htb1⁺* (YGF472), *htb1-G52D* (YGF475), and *htb1-P102L* (YGF478) cells. *E*, the growth of *htb1-G52D* (YGF426) and *htb1-P102L* (YGF427) integrated with overexpressed *Padh21-brl1-5FLAG* at *lys1⁺* in the presence of 36°C, MMS, bleomycin, phleomycin, and UV. *F*, the growth of *htb1⁺* (YGF471), *htb1-G52D* (YGF474), and *htb1-P102L* (YGF477) integrated with overexpressed *Pnmt1-brl1-5FLAG* at *leu1⁺* in the presence of 36°C, MMS, bleomycin, phleomycin, and UV. *G*, the growth of *htb1⁺* (YGF472), *htb1-G52D* (YGF475), and *htb1-P102L* (YGF478) integrated with overexpressed *Pnmt1-rhp6-CBP* at *leu1⁺* in the presence of 36°C, MMS, bleomycin, phleomycin, and UV.

Figure S9

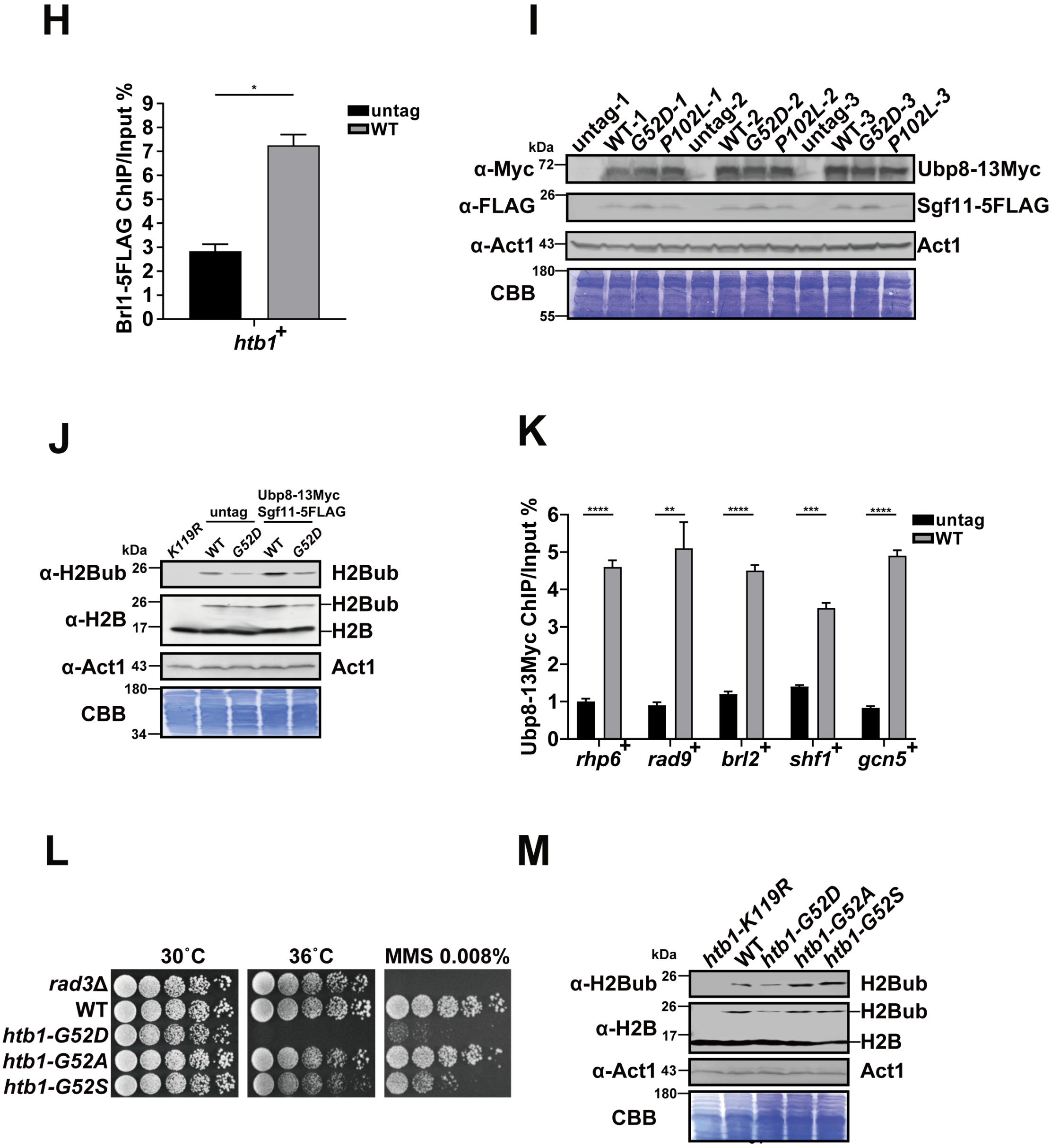

Figure S9 (related to Figure 9). *H*, the ChIP-qPCR of Brl1-5FLAG enrichment at *htb1*$^{+}$. The Brl1-5FLAG enrichments from the untag control (TK8) and WT (YGF479) are shown as the percentages of IP DNA relative to input DNA. The data from two independent biological repeats are averaged. Error bars represent SEMs. Student's t-test is used for comparisons between the untag control and WT. *I*, the immunoblots of Ubp8-13Myc and Sgf11-5FLAG in *htb1*$^{+}$ (YGF502), *htb1-G52D* (YGF503), and *htb1-P102L* (YGF504) cells. *J*, the immunoblots of H2Bub in untagged WT (TK8) and *htb1-G52D* (YGF277) cells as well as WT (YGF502) and *htb1-G52D* (YGF503) tagged with Ubp8-13Myc and Sgf11-5FLAG. *K*, the ChIP-qPCR of Ubp8-13Myc enrichment at *rhp6*$^{+}$, *rad9*$^{+}$, *brl2*$^{+}$, *shf1*$^{+}$, and *gcn5*$^{+}$ from the untag control (TK8) and WT (YGF502). The data from three independent biological repeats are averaged. Error bars represent SEMs. Student's t-test is used for comparisons between the untag control and WT. *L*, the growth of *htb1-G52D* (YGF277), *htb1-G52A* (YGF459), and *htb1-G52S* (YGF460) in the presence of 36°C and MMS. *M*, immunoblots of H2Bub in *htb1-G52D* (YGF277), *htb1-G52A* (YGF459), and *htb1-G52S* (YGF460) cells.

Figure S10

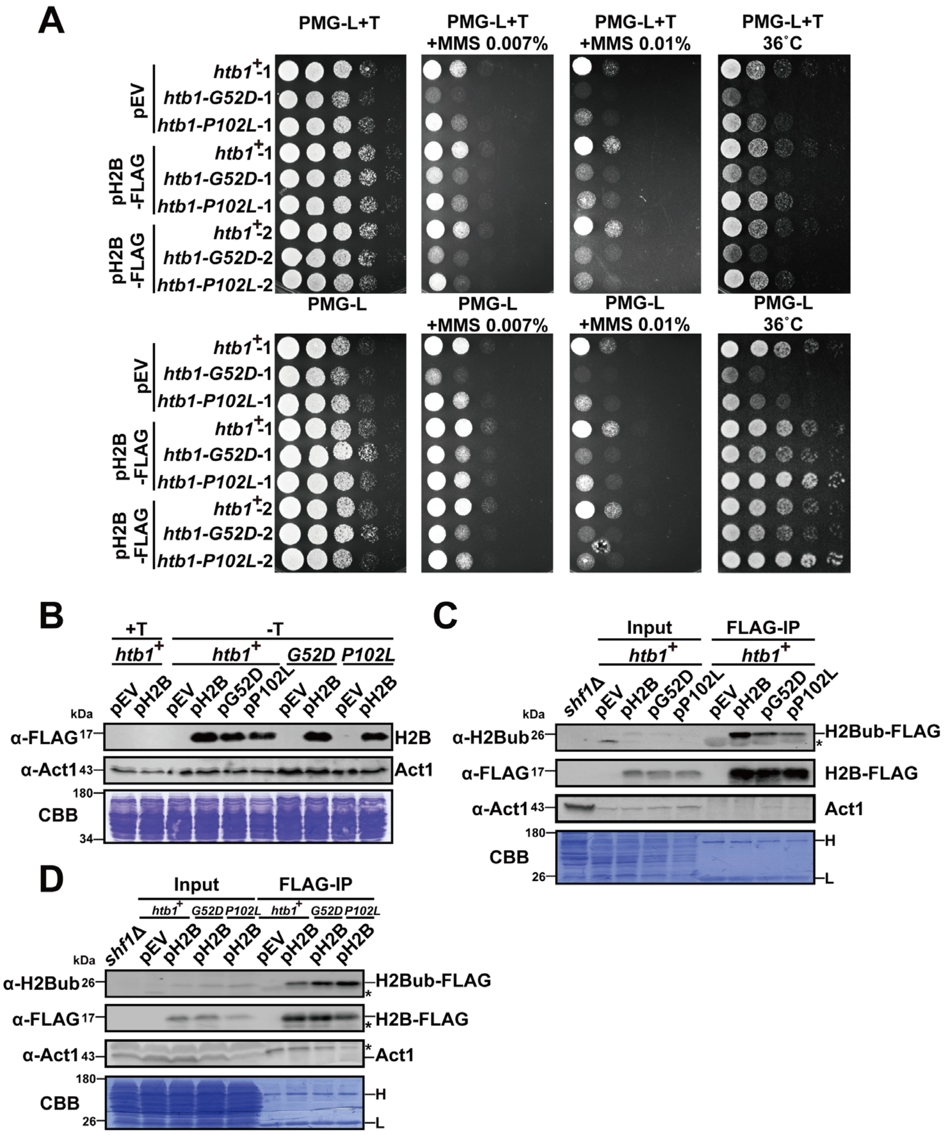

Figure S10 (related to Figure 10). *A*, MMS and temperature sensitivities of the second independent biological repeats of *htb1*$^{+}$/pEV (YGF311), *htb1-G52D*/pEV (YGF312), *htb1-P102L*/pEV (YGF313), *htb1*$^{+}$/pH2B-FLAG (YGF314), *htb1-G52D*/pH2B-FLAG (YGF315), and *htb1-P102L*/pH2B-FLAG (YGF316) strains. pEV is the empty vector of pREP1 plasmid. pH2B-FLAG indicates the C-terminal FLAG tagged *htb1*$^{+}$ gene under control of *nmt1*$^{+}$ promoter in pREP1. T indicates thiamine. The images are reuse of Figure 10*B*, additionally with 2nd repeats of *htb1-G52D/P102L* transformed with pH2B-FLAG. *B*, immunoblots of H2B-FLAG, H2BG52D-FLAG, and H2BP102L-FLAG overexpression in *htb1*$^{+}$ (TK8), *htb1-G52D* (YGF277), and *htb1-P102L* (YGF279) cell extracts with the FLAG antibody. pEV is the empty vector of pREP1. pH2B, pG52D, and pP102L indicate FLAG tag at the C-terminus of the *htb1*$^{+}$, *htb1-G52D*, and *htb1-P102L* genes in pREP1, respectively. *C*, immunoblots of H2Bub-FLAG in the FLAG IP from another repeat of WT transformed with plasmids of H2B-FLAG, H2BG52D-FLAG, and H2BP102L-FLAG. *D*, immunoblots of H2Bub-FLAG in the FLAG IP from another repeat of *htb1*$^{+}$, *htb1-G52D*, and *htb1-P102L* strains transformed with the H2B-FLAG plasmid. Asterisk indicates cross-reactive protein bands. H and L indicates heavy and light chain of antibody, respectively.

Figure S11

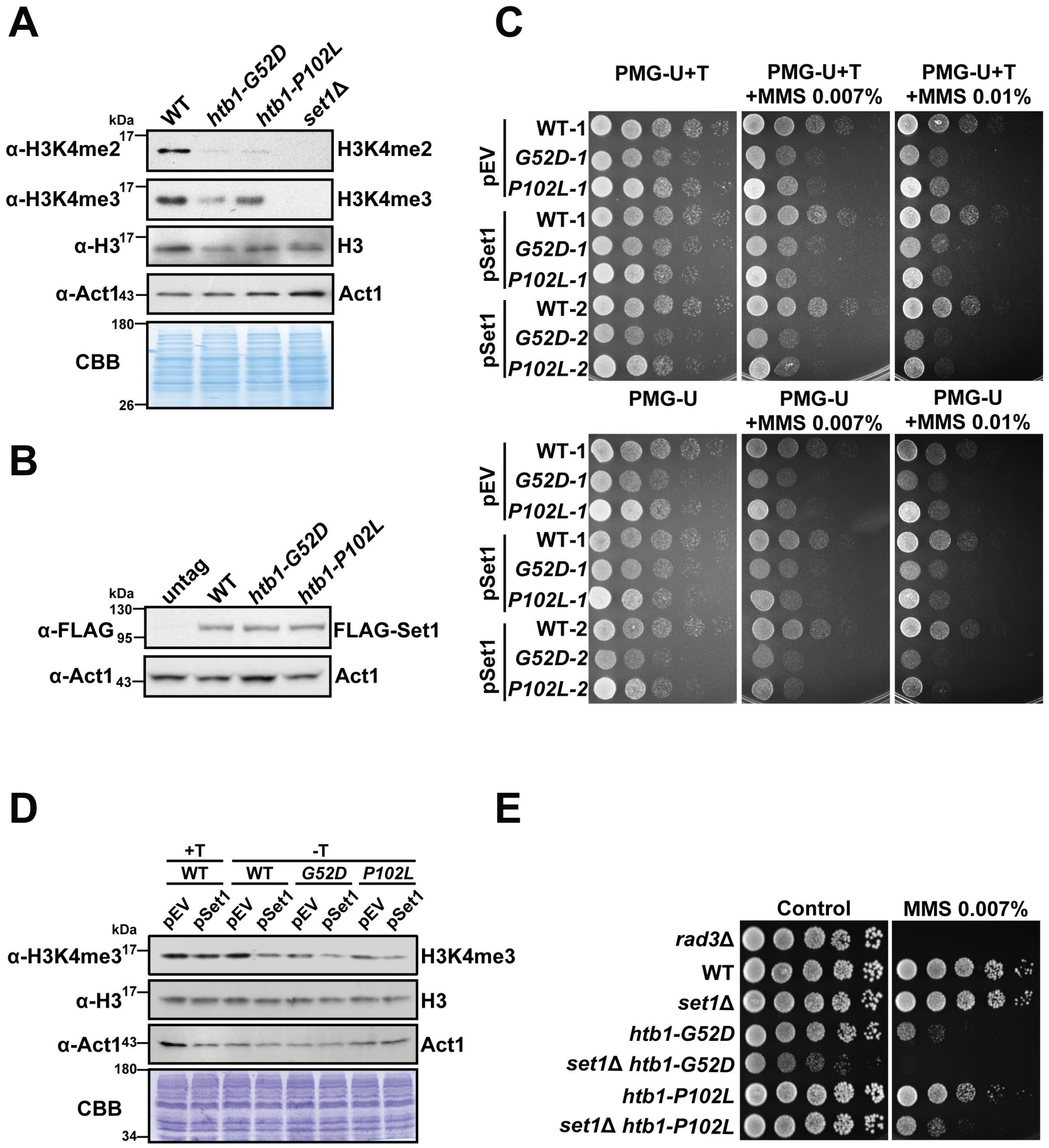

Figure S11. *A*, immunoblots of the abundance of H3K4me2 and H3K4me3 in WT (TK8), *htb1-G52D* (YGF277), *htb1-P102L* (YGF279), and *set1*Δ (FY14106) strains. *B*, immunoblots of FLAG-tagged Set1 in untag (YGF401), WT (YGF402), *htb1-G52D* (YGF403), and *htb1-P102L* (YGF404) strains, in which the native $set1^{+}$ gene is deleted and another copy of FLAG-*set1* under the $nmt1^{+}$ promoter is integrated at $leu1^{+}$. *C*, MMS sensitivities of WT/pEV (YGF305), *htb1-G52D*/pEV (YGF306), *htb1-P102L*/pEV (YGF307), WT/pSet1 (YGF308), *htb1-G52D*/pSet1 (YGF309), and *htb1-P102L*/pSet1 (YGF310) strains with and without Set1 overexpression. pEV is the empty vector of pDUAL; pSet1 indicates the $set1^{+}$ gene under $nmt1^{+}$ promoter in pDUAL. *D*, immunoblots of H3K4me3 abundance in WT/pEV (YGF305), *htb1-G52D*/pEV (YGF306), *htb1-P102L*/pEV (YGF307), WT/pSet1 (YGF308), *htb1-G52D*/pSet1 (YGF309), and *htb1-P102L*/pSet1 (YGF310) strains before or after Set1 overexpression. *E*, MMS sensitivities of *set1*Δ *htb1-G52D* (YGF297) and *set1*Δ *htb1-P102L* (YGF298) mutants.

Figure S11

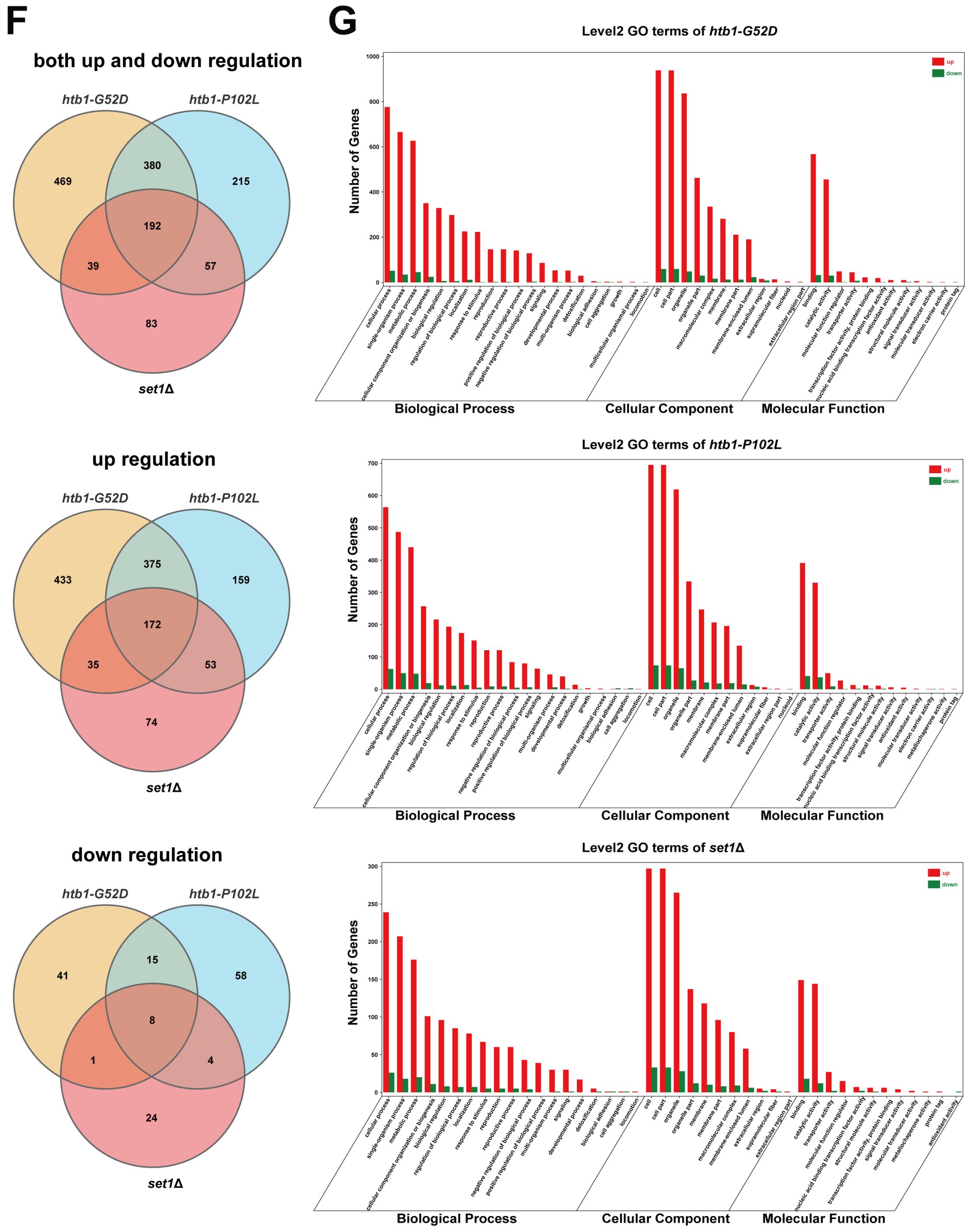

Figure S11. *F*, venn graph of differentially-regulated, up-regulated, and down-regulated genes among *htb1-G52D* (YGF277), *htb1-P102L* (YGF279) and *set1*Δ (FY14106) mutants. *G*, the analysis of the GO level2 terms including biological process, cellular component, and molecular function in *htb1-G52D* (YGF277), *htb1-P102L* (YGF279) and *set1*Δ (FY14106) mutants.

Table S1

Yeast strains used in this study

Strain	Genotype	Source
LD297	h^{-} leu1-32 ura4-D18 ade6-M210 his3-D1 rad3Δ::LEU2	L. Du
TK8	h^{+} ade6-M216 leu1-32 ura4-D18	T. Kelly
J2172	h^{-} cdc10-M17 ura4-D18 ade6-704 leu1-32::[Padh1-hENT1-$leu1^{+}$] his7-366::[Padh1-hsv-tk-$his7^{+}$]	A. Carr
Ets12	h^{-} ade6-704 leu1-32 ura4-D18 chk1Δ::kanMX6	A. Carr
Ets13	h^{+} ade6-704 leu1-32 ura4-D18 cds1Δ::kanMX6	A. Carr
DY49	h^{+} leu1-32 his3-D1 arg3Δ::HO site-natMX6 ars1::Pnmt41-HO-$his3^{+}$	L. Du
DY2407	h^{-} leu1-32 his3-D1 ura4-294 arg3Δ::HO site-natMX6 ars1::[Pnmt41-HO-$his3^{+}$] lys1-131::[Pdis1-mCherry-LacI-$lys1^{+}$] erg7ter:: lacOrepeat($ura4^{+}$) rad52-2CFP::hphMX6 ssb1-YFP-FLAG-6His::$leu1^{+}$	L. Du
YHMZ31	h^{+} leu1-32 his3-D1 ura4-294 arg3Δ::HO site-natMX6 ars1::Pnmt41-HO-$his3^{+}$ rad52-2CFP::hphMX6 ssb1-YFP-FLAG-6His::$leu1^{+}$ exo1Δ::$ura4^{+}$	D. Kong
BN1	h^{+} ade6-M210 leu1-32 ura4-D18 gcn5Δ::kanMX6	Bioneer
FY10067	h^{+} ade6-? leu1-32 chk1-3HA::LEU2	NBRP
FY11064	h^{-} leu1-32 ura4-D18 cds1-2HA-6His-$ura4^{+}$	NBRP
FY14106	h^{+} leu1-32 ura4-D18 set1Δ::kanMX6	NBRP
FY14135	h^{+} ade6-M210 leu1-32 ura4-D18 rad51Δ::$ura4^{+}$	NBRP
FY17274	h^{-} leu1-32 htb1-G52D	NBRP
FY17275	h^{-} leu1-32 htb1-P102L	NBRP
FY18537	h^{-} leu1-32 ura4-D18 rad51Δ::hphMX6	NBRP
FY20970	h^{90} leu1-32 ura4-D18 ade6-? ubc13Δ::$ura4^{+}$	NBRP
FY20976	h^{90} leu1-32 ura4-D18 ade6-? rhp18Δ::$ura4^{+}$	NBRP
YGF1	h^{-} ura4-D18 htb1::kanMX6	This study
YGF226	h^{-} ura4-D18 htb1-K119R::kanMX6	This study
YGF266	h^{-} ura4-D18 pku70Δ::$ura4^{+}$	This study
YGF275	h^{+} ade6-M210 leu1-32 ura4-D18 htb1-E34K::kanMX6	This study
YGF276	h^{-} leu1-32 his3-D1 ura4-294 arg3Δ::HO site-natMX6 ars1::[Pnmt41-HO-$his3^{+}$] lys1-131::[Pdis1-mCherry-LacI-$lys1^{+}$] erg7ter:: lacOrepeat($ura4^{+}$) rad52-2CFP::hphMX6 ssb1-YFP-FLAG-6His::$leu1^{+}$ htb1-G52D::kanMX6	This study
YGF277	h^{-} ade6? leu1-32 ura4-D18 htb1-G52D	This study
YGF278	h^{-} leu1-32 his3-D1 ura4-294 arg3Δ::HO site-natMX6 ars1::[Pnmt41-HO-$his3^{+}$] lys1-131::[Pdis1-mCherry-LacI-$lys1^{+}$] erg7ter:: lacOrepeat($ura4^{+}$) rad52-2CFP::hphMX6 ssb1-YFP-FLAG-6His::$leu1^{+}$ htb1-P102L::kanMX6	This study
YGF279	h^{-} ade6? leu1-32 ura4-D18 htb1-P102L	This study
YGF281	h^{-} cdc10-M17 ura4-D18 ade6-704 leu1-32::[Padh1-hENT1-$leu1^{+}$] his7-366::[Padh1-hsv-tk-$his7^{+}$] htb1-G52D::kanMX6	This study
YGF282	h^{-} cdc10-M17 ura4-D18 ade6-704 leu1-32::[Padh1-hENT1-$leu1^{+}$] his7-366::[Padh1-hsv-tk-$his7^{+}$] htb1-P102L::kanMX6	This study
YGF287	h? ade6? leu1-32 ura4-D18 htb1-G52D cds1Δ::kanMX6	This study
YGF288	h? ade6? leu1-32 ura4-D18 htb1-P102L cds1Δ::kanMX6	This study
YGF289	h? ade6? leu1-32 ura4? htb1-G52D chk1-3HA::LEU2	This study
YGF290	h? ade6? leu1-32 ura4? htb1-P102L chk1-3HA::LEU2	This study
YGF291	h? ade6? leu1-32 ura4-D18 htb1-G52D chk1Δ::kanMX6	This study
YGF292	h? ade6? leu1-32 ura4-D18 htb1-P102L chk1Δ::kanMX6	This study
YGF293	h? ade6? leu1? ura4-D18 htb1-G52D pku70Δ::$ura4^{+}$	This study
YGF294	h? ade6? leu1? ura4-D18 htb1-P102L pku70Δ::$ura4^{+}$	This study
YGF295	h? ade6? leu1-32 ura4-D18 htb1-G52D ubc13Δ::$ura4^{+}$	This study
YGF296	h? ade6? leu1-32 ura4-D18 htb1-P102L ubc13Δ::$ura4^{+}$	This study
YGF297	h? ade6? leu1-32 ura4-D18 htb1-G52D set1Δ::kanMX6	This study
YGF298	h? ade6? leu1-32 ura4-D18 htb1-P102L set1Δ::kanMX6	This study
YGF299	h? ade6? leu1-32 ura4-D18 htb1-G52D rhp18Δ::$ura4^{+}$	This study
YGF300	h? ade6? leu1-32 ura4-D18 htb1-P102L rhp18Δ::$ura4^{+}$	This study
YGF301	h? ade6? leu1-32 ura4-D18 htb1-G52D rad51Δ::hphMX6	This study

YGF302	*h? ade6? leu1-32 ura4-D18 htb1-P102L rad51Δ::hphMX6*	This study
YGF303	*h? ade6? leu1-32 ura4-D18 htb1-G52D cds1-2HA-6His-ura4*$^{+}$	This study
YGF304	*h? ade6? leu1-32 ura4-D18 htb1-P102L cds1-2HA-6His-ura4*$^{+}$	This study
YGF305	h^{+} *ade6-M216 leu1-32 ura4-D18 [pDUAL]*	This study
YGF306	h^{-} *ade6? leu1-32 ura4-D18 htb1-G52D [pDUAL]*	This study
YGF307	h^{-} *ade6? leu1-32 ura4-D18 htb1-P102L [pDUAL]*	This study
YGF308	h^{+} *ade6-M216 leu1-32 ura4-D18 [pDUAL-Pnmt1-set1]*	This study
YGF309	h^{-} *ade6? leu1-32 ura4-D18 htb1-G52D [pDUAL-Pnmt1-set1]*	This study
YGF310	h^{-} *ade6? leu1-32 ura4-D18 htb1-P102L [pDUAL-Pnmt1-set1]*	This study
YGF311	h^{+} *ade6-M216 leu1-32 ura4-D18 [pREP1]*	This study
YGF312	h^{-} *ade6? leu1-32 ura4-D18 htb1-G52D [pREP1]*	This study
YGF313	h^{-} *ade6? leu1-32 ura4-D18 htb1-P102L [pREP1]*	This study
YGF314	h^{+} *ade6-M216 leu1-32 ura4-D18 [pREP1-htb1-FLAG]*	This study
YGF315	h^{-} *ade6? leu1-32 ura4-D18 htb1-G52D [pREP1-htb1-FLAG]*	This study
YGF316	h^{-} *ade6? leu1-32 ura4-D18 htb1-P102L [pREP1-htb1-FLAG]*	This study
YGF317	h^{+} *ade6-M210 leu1-32 ura4-D18 htb1-E75Q::kanMX6*	This study
YGF318	h^{+} *ade6-M210 leu1-32 ura4-D18 htb1-G103W::kanMX6*	This study
YGF319	h^{+} *ade6-M216 leu1-32 ura4-D18 [pREP1-htb1-G52D-FLAG]*	This study
YGF320	h^{+} *ade6-M216 leu1-32 ura4-D18 [pREP1-htb1-P102L-FLAG]*	This study
YGF321	h^{+} *ade6-M210 leu1-32 ura4-D18 htb1-E112Q::kanMX6*	This study
YGF322	h^{+} *ade6-M210 leu1-32 ura4-D18 htb1-R98C::kanMX6*	This study
YGF323	h^{+} *ade6-M210 leu1-32 ura4-D18 htb1-F69L::kanMX6*	This study
YGF324	h^{+} *ade6-M210 leu1-32 ura4-D18 htb1-D67N::kanMX6*	This study
YGF325	h^{+} *ade6-M210 leu1-32 ura4-D18 htb1-P102S::kanMX6*	This study
YGF326	h^{+} *ade6-M210 leu1-32 ura4-D18 htb1-E92D::kanMX6*	This study
YGF328	h^{+} *ade6-M210 leu1-32 ura4-D18 htb1-E112K::kanMX6*	This study
YGF329	h^{+} *ade6-M210 leu1-32 ura4-D18 htb1-E34D::kanMX6*	This study
YGF330	h^{+} *ade6-M210 leu1-32 ura4-D18 htb1-E70Q::kanMX6*	This study
YGF331	h^{+} *ade6-M210 leu1-32 ura4-D18 htb1-Q46E::kanMX6*	This study
YGF332	h^{+} *ade6-M210 leu1-32 ura4-D18 htb1-D50N::kanMX6*	This study
YGF340	h^{+} *ade6-M216 leu1-32 ura4-D18 [pREP1-crb2]*	This study
YGF341	h^{-} *ade6? leu1-32 ura4-D18 htb1-G52D [pREP1-crb2]*	This study
YGF342	h^{-} *ade6? leu1-32 ura4-D18 htb1-P102L [pREP1-crb2]*	This study
YGF357	*h? leu1? his3? ura4-? exo1Δ::ura4*$^{+}$	This study
YGF358	*h? ade6? leu1-32 his3? ura4-? exo1Δ::ura4*$^{+}$ *htb1-G52D*	This study
YGF359	*h? ade6? leu1-32 his3? ura4-? exo1Δ::ura4*$^{+}$ *htb1-P102L*	This study
YGF393	h^{+} *ade6-M216 leu1-32 ura4-D18 rqh1Δ::kanMX6*	This study
YGF394	h^{-} *ade6? leu1-32 ura4-D18 htb1-G52D rqh1Δ::kanMX6*	This study
YGF395	h^{-} *ade6? leu1-32 ura4-D18 htb1-P102L rqh1Δ::kanMX6*	This study
YGF396	h^{+} *ade6-M216 leu1-32 ura4-D18 mus81Δ::kanMX6*	This study
YGF397	h^{-} *ade6? leu1-32 ura4-D18 htb1-G52D mus81Δ::kanMX6*	This study
YGF398	h^{-} *ade6? leu1-32 ura4-D18 htb1-P102L mus81Δ::kanMX6*	This study
YGF401	h^{+} *ura4-D18 set1Δ::kanMX6 leu1-32::[pDUAL-Pnmt1-set1-leu1*$^{+}$*]*	This study
YGF402	h^{+} *ura4-D18 set1Δ::kanMX6 leu1-32::[pDUAL-Pnmt1-FLAG-set1-leu1*$^{+}$*]*	This study
YGF403	*h? ade6? ura4-D18 htb1-G52D set1Δ::kanMX6 leu1-32::[pDUAL-Pnmt1-FLAG-set1-leu1*$^{+}$*]*	This study
YGF404	*h? ade6? ura4-D18 htb1-P102L set1Δ::kanMX6 leu1-32::[pDUAL-Pnmt1-FLAG-set1-leu1*$^{+}$*]*	This study
YGF408	h^{-} *leu1-32 htb1-G52D ubp8-1*	This study
YGF415	h^{+} *ade6-M216 leu1-32 ura4-D18 ubp8Δ::kanMX6*	This study
YGF416	h^{-} *leu1-32 ura4-D18 htb1-G52D ubp8Δ::kanMX6*	This study
YGF417	h^{-} *leu1-32 ura4-D18 htb1-P102L ubp8Δ::kanMX6*	This study
YGF418	*h? leu1-32 ura4-D18 htb1-G52D gcn5Δ::kanMX6*	This study
YGF419	*h? leu1-32 ura4-D18 htb1-P102L gcn5Δ::kanMX6*	This study
YGF422	h^{-} *leu1-32 ura4-D18 htb1-G52D lys1Δ::[pHBKA21-hphMX6]*	This study
YGF424	h^{-} *leu1-32 ura4-D18 htb1-P102L lys1Δ::[pHBKA21-hphMX6]*	This study
YGF430	h^{+} *ade6-M216 leu1-32 ura4-D18 ubp8Δ::hphMX6*	This study
YGF431	h^{+} *ade6-M216 leu1-32 ura4-D18 shf1Δ::hphMX6*	This study
YGF432	*h? ade6? leu1-32 ura4-D18 htb1-G52D ubp8Δ::kanMX6 shf1Δ::hphMX6*	This study
YGF433	h^{-} *leu1-32 ura4-D18 htb1-P102L ubp8Δ::kanMX6 shf1Δ::hphMX6*	This study

YGF442	h^+ ade6-M216 leu1-32 ura4-D18 htb1-P102L K119R::kanMX6	This study
YGF443	h? ade6? leu1? ura4? ubp8Δ::hphMX6 htb1-D67N::kanMX6	This study
YGF444	h^+ ade6-M216 leu1-32 ura4-D18 htb1-G52D K119R::kanMX6	This study
YGF455	h^- ade6? leu1? ura4? ubp8Δ::hphMX6	This study
YGF457	h? ade6-M216 leu1-32 ura4-D18 htb1-G52D gcn5Δ::kanMX6 shf1Δ::hphMX6	This study
YGF458	h? ade6-M216 leu1-32 ura4-D18 htb1-P102L gcn5Δ::kanMX6 shf1Δ::hphMX6	This study
YGF459	h^+ ade6-M216 leu1-32 ura4-D18 htb1-G52A::kanMX6	This study
YGF460	h^+ ade6-M216 leu1-32 ura4-D18 htb1-G52S::kanMX6	This study
YGF462	h^+ arg3Δ::HO site-natMX6 ars1::Pnmt41-HO-$his3^+$ htb1-G52D::kanMX6 rad51-5FLAG::hph	This study
YGF463	h^+ arg3Δ::HO site-natMX6 ars1::Pnmt41-HO-$his3^+$ htb1-P102L::kanMX6 rad51-5FLAG::hph	This study
YGF466	h^+ leu1-32 his3-D1 arg3Δ::HO site-natMX6 ars1::Pnmt41-HO-$his3^+$ rad51-5FLAG::hph	This study
YGF467	h^+ ade6-M216 leu1-32 ura4-D18 lys1Δ::[pHBKA21-hphMX6]	This study
YGF468	h^+ ade6-M216 leu1-32 ura4-D18 lys1Δ::[pHBKA21-brl1-5FLAG-hphMX6]	This study
YGF470	h^+ ade6-M216 ura4-D18 leu1-32::[pJK148-Pnmt1-$leu1^+$]	This study
YGF471	h^+ ade6-M216 ura4-D18 leu1-32::[pJK148-Pnmt1-brl1-5FLAG-$leu1^+$]	This study
YGF472	h^+ ade6-M216 ura4-D18 leu1-32::[pJK148-Pnmt1-rhp6-CBP-$leu1^+$]	This study
YGF473	h^- ade6? ura4-D18 htb1-G52D leu1-32::[pJK148-Pnmt1-$leu1^+$]	This study
YGF474	h^- ade6? ura4-D18 htb1-G52D leu1-32::[pJK148-Pnmt1-brl1-5FLAG-$leu1^+$]	This study
YGF475	h^- ade6? ura4-D18 htb1-G52D leu1-32::[pJK148-Pnmt1-rhp6-CBP-$leu1^+$]	This study
YGF476	h^- ade6? ura4-D18 htb1-P102L leu1-32::[pJK148-Pnmt1-$leu1^+$]	This study
YGF477	h^- ade6? ura4-D18 htb1-P102L leu1-32::[pJK148-Pnmt1-brl1-5FLAG-$leu1^+$]	This study
YGF478	h^- ade6? ura4-D18 htb1-P102L leu1-32::[pJK148-Pnmt1-rhp6-CBP-$leu1^+$]	This study
YGF479	h^+ ade6-M216 leu1-32 ura4-D18 brl2-3HA::natMX6 brl1-5FLAG::hphMX6 rhp6-CBP::$leu1^+$ shf1-13Myc::kanMX6	This study
YGF480	h^+ leu1-32 his3-D1 arg3Δ::HO site-natMX6 ars1::P41nmt1-HO-$his3^+$ htb1-5FLAG::hphMX6	This study
YGF482	h^+ ade6-M216 leu1-32 ura4-D18 sgf11Δ::kanMX6	This study
YGF483	h^+ ade6-M216 leu1-32 ura4-D18 htb1-G52D sgf11Δ::kanMX6	This study
YGF484	h^+ ade6-M216 leu1-32 ura4-D18 htb1-P102L sgf11Δ::kanMX6	This study
YGF485	h^+ ade6-M216 leu1-32 ura4-D18 sgf11-Q64*::hphMX6	This study
YGF486	h^+ ade6-M216 leu1-32 ura4-D18 htb1-G52D sgf11-Q64*::hphMX6	This study
YGF487	h^+ ade6-M216 leu1-32 ura4-D18 htb1-P102L sgf11-Q64*::hphMX6	This study
YGF488	h^+ ade6-M216 leu1-32 ura4-D18 sgf11-R85A::hphMX6	This study
YGF489	h^+ ade6-M216 leu1-32 ura4-D18 htb1-G52D sgf11-R85A::hphMX6	This study
YGF490	h^+ ade6-M216 leu1-32 ura4-D18 htb1-P102L sgf11-R85A::hphMX6	This study
YGF491	h^+ ade6-M216 leu1-32 ura4-D18 htb1-G52D P102L::kanMX6	This study
YGF492	h^+ leu1-32 his3-D1 arg3Δ::HO site-natMX6 ars1::P41nmt1-HO-$his3^+$ htb1-P102L-5FLAG::hphMX6	This study
YGF493	h^+ ade6-M216 leu1-32 ura4-D18 lys1Δ::[pHBKA21-htb1-5FLAG-hphMX6]	This study
YGF494	h^- leu1-32 ura4-D18 htb1-G52D lys1Δ::[pHBKA21-htb1-5FLAG-hphMX6]	This study
YGF495	h^- leu1-32 ura4-D18 htb1-P102L lys1Δ::[pHBKA21-htb1-5FLAG-hphMX6]	This study
YGF496	h? ade6? leu1-32 ura4-D18 htb1-G52D brl2-3HA::natMX6 brl1-5FLAG::hphMX6 rhp6-CBP::$leu1^+$ shf1-13Myc::kanMX6	This study
YGF497	h? ade6? leu1-32 ura4-D18 htb1-P102L brl2-3HA::natMX6 brl1-5FLAG::hphMX6 rhp6-CBP::$leu1^+$ shf1-13Myc::kanMX6	This study
YGF498	h^+ ade6-M216 leu1-32 ura4-D18 lys1Δ::[pHBKA21-htb1-G52D-5FLAG-hphMX6]	This study

YGF500	*h⁺ ade6-M216 leu1-32 ura4-D18 lys1Δ::[pHBKA21-htb1-P102L-5FLAG-hphMX6]*	This study
YGF502	*h⁺ ade6-M216 leu1-32 ura4-D18 ubp8-13Myc::kanMX6 sgf11-5FLAG::hphMX6*	This study
YGF503	*h⁻ ade6? leu1-32 ura4-D18 htb1-G52D ubp8-13Myc::kanMX6 sgf11-5FLAG::hphMX6*	This study
YGF504	*h⁻ ade6? leu1-32 ura4-D18 htb1-P102L ubp8-13Myc::kanMX6 sgf11-5FLAG::hphMX6*	This study

Table S2

Plasmids used in this study

Plasmid	Alias	Description	Source
pGF73	pFA6a-htb1-G52D-kanMX6	pFA6a containing *htb1-G52D* mutation and *kanMX6*	This study
pGF74	pFA6a-htb1-P102L-kanMX6	pFA6a containing *htb1-P102L* mutation and *kanMX6*	This study
pGF77	pBluescript-leu1 (3'Δ)	pBluescript containing 3' truncated *leu1*	D. Kong
pGF80	pREP1-htb1-FLAG	pREP1 containing $nmt1^+$ promoter and FLAG tagged *htb1* and *LEU2*	NBRP(FYP2103)
pGF81	pDUAL	pUC119 containing truncated *leu1* and $ura4^+$	Lab stock
pGF82	pDUAL-Pnmt1-set1	pDUAL expressing *set1* from $nmt1^+$ promoter	L. Du
pGF85	pDUAL-Pnmt1-FLAG-set1	pDUAL expressing FLAG tagged *set1* from $nmt1^+$ promoter	L. Du
pGF86	pFA6a-htb1-E112K-kanMX6	pFA6a containing *htb1-E112K* mutation and *kanMX6*	This study
pGF87	pFA6a-htb1-G103W-kanMX6	pFA6a containing *htb1-G103W* mutation and *kanMX6*	This study
pGF88	pREP1-htb1-G52D-FLAG	pREP1 containing $nmt1^+$ promoter and FLAG tagged *htb1-G52D* and *LEU2*	This study
pGF89	pREP1-htb1-P102L-FLAG	pREP1 containing $nmt1^+$ promoter and FLAG tagged *htb1-P102L* and *LEU2*	This study
pGF90	pFA6a-htb1-E112Q-kanMX6	pFA6a containing *htb1-E112Q* mutation and *kanMX6*	This study
pGF92	pFA6a-htb1-E75Q-kanMX6	pFA6a containing *htb1-E75Q* mutation and *kanMX6*	This study
pGF93	pFA6a-htb1-P102S-kanMX6	pFA6a containing *htb1-P102S* mutation and *kanMX6*	This study
pGF94	pFA6a-htb1-R98C-kanMX6	pFA6a containing *htb1-R98C* mutation and *kanMX6*	This study
pGF95	pFA6a-htb1-E92D-kanMX6	pFA6a containing *htb1-E92D* mutation and *kanMX6*	This study
pGF96	pFA6a-htb1-E70Q-kanMX6	pFA6a containing *htb1-E70Q* mutation and *kanMX6*	This study
pGF97	pFA6a-htb1-F69L-kanMX6	pFA6a containing *htb1-F69L* mutation and *kanMX6*	This study
pGF98	pFA6a-htb1-D67N-kanMX6	pFA6a containing *htb1-D67N* mutation and *kanMX6*	This study
pGF99	pFA6a-htb1-E34K-kanMX6	pFA6a containing *htb1-E34K* mutation and *kanMX6*	This study
pGF100	pFA6a-htb1-E34D-kanMX6	pFA6a containing *htb1-E34D* mutation and *kanMX6*	This study
pGF101	pFA6a-htb1-Q46E-kanMX6	pFA6a containing *htb1-Q46E* mutation and *kanMX6*	This study
pGF104	pFA6a-htb1-D50N-kanMX6	pFA6a containing *htb1-D50N* mutation and *kanMX6*	This study
pGF108	pREP1-crb2-6His	pREP1 containing $nmt1^+$ promoter and 6His tagged *crb2* and *LEU2*	This study
pGF124	pREP2	pUC119 containing $nmt1^+$ promoter and $ura4^+$	Lab stock
pGF133	pREP1	pUC119 containing $nmt1^+$ promoter and *LEU2*	Lab stock
pGF147	pBluescript-sgf11-Q64*-hphMX6	pBluescript containing *sgf11-Q64** mutation and *hphMX6*	This study
pGF154	pBluescript-	pBluescript containing *sgf11-R85A* mutation and	This study

	sgf11-R85A-hphMX6	*hphMX6*	
pGF156	pHBKA21	pUC119 containing *adh21*$^+$ promoter and *lys1* homology and *hphMX6*	Q. Jin
pGF177	pHBKA21-htb1-5FLAG-hphMX6	pHBKA21 containing *adh21*$^+$ promoter and 5FLAG tagged *htb1* and *hphMX6*	This study
pGF178	pFA6a-htb1-G52D P102L-kanMX6	pFA6a containing *htb1-G52D P102L* mutation and *kanMX6*	This study
pGF180	pHBKA21-brl1-5FLAG-hphMX6	pHBKA21 containing *adh21*$^+$ promoter and 5FLAG tagged *brl1* and *hphMX6*	This study
pGF182	pFA6a-htb1-G52D K119R-kanMX6	pFA6a containing *htb1-G52D K119R* mutation and *kanMX6*	This study
pGF183	pFA6a-htb1-P102L K119R-kanMX6	pFA6a containing *htb1-P102L K119R* mutation and *kanMX6*	This study
pGF186	pFA6a-htb1-G52A-kanMX6	pFA6a containing *htb1-G52A* mutation and *kanMX6*	This study
pGF188	pFA6a-htb1-G52S-kanMX6	pFA6a containing *htb1-G52S* mutation and *kanMX6*	This study
pGF199	pJK148-Pnmt1-brl1-5FLAG	pJK148 containing *nmt1*$^+$ promoter and 5FLAG tagged *brl1* and *leu1*$^+$	This study
pGF200	pJK148-Pnmt1-rhp6-CBP-leu1$^+$	pJK148 containing *nmt1*$^+$ promoter and CBP tagged *rhp6* and *leu1*$^+$	This study
pGF207	pHBKA21-htb1-G52D-5FLAG-hphMX6	pHBKA21 containing *adh21*$^+$ promoter and 5FLAG tagged *htb1-G52D* and *hphMX6*	This study
pGF209	pHBKA21-htb1-P102L-5FLAG-hphMX6	pHBKA21 containing *adh21*$^+$ promoter and 5FLAG tagged *htb1-P102L* and *hphMX6*	This study

Table S3

Antibodies used in this study

Antibody	Description	Source
H2AS129ph (γH2A) (rabbit pAb)	IB: 1:2000	Abcam (ab17353)
H2A (rabbit pAb)	IB: 1:2000	PTM-Biolab (1008)
H3K4me2 (rabbit pAb)	IB: 1:300	Abcam (ab7766)
H3K4me3 (rabbit pAb)	IB: 1:500	Abcam (ab8580)
H3K9ac (rabbit pAb)	IB: 1:1000	Abcam (ab4441)
H3 (rabbit pAb)	IB: 1:5000	Abcam (ab1791)
H2BK119ub (rabbit mAb)	IB: 1:1000; ChIP: 1:200	CST (5546)
H2BK5acK10acK15ac (rabbit pAb)	IB: 1:200	In-lab
H2B (rabbit pAb)	IB: 1:1000 ChIP: 1:500	GeneTex (64122)
H2B (rabbit pAb)	IB: 1:1000	In-lab
Rpa1 (rabbit pAb)	IB: 1:1000	In-lab
Actin (rabbit mAb)	IB: 1:50000	Abclonal (AC026)
HA (mouse mAb)	IB: 1:1000	Sigma (H3663)
FLAG (mouse mAb)	IB: 1:1000	Sigma (F1804)
GFP (mouse mAb)	IB: 1:1000	Bioworld (AP0675M)
Myc (mouse mAb)	IB: 1:1000	Sigma (ab32)
CBP (mouse mAb)	IB: 1:1000	Genscript (A01798)
IgG-HRP (anti-rabbit)	IB: 1:10000	Bioeasytech (BE0101)
IgG-HRP (anti-mouse)	IB: 1:10000	Bioeasytech (BE0102)
IgG-Alexa Fluor Plus 800 (anti-mouse)	IB: 1:10000	Thermo (A32730)
IgG-Alexa Fluor Plus 800 (anti-rabbit)	IB: 1:10000	Thermo (A32735)
IgG-Alexa Fluor Plus 680 (anti-rabbit)	IB: 1:10000	Thermo (A32802)

Table S8

Raw data for homologous recombination assay

Sample	Leu^+ (mean)	Ura^+ (mean)	HR frequency (%)
WT-1	2,755	11,950	23.1
WT-2	2,855	13,750	20.8
WT-3	1,337	9,000	14.9
*rad51*Δ-1	1	3,200	0.03
*rad51*Δ-2	0	1,360	0
*rad51*Δ-3	1	5,510	0.02
htb1-G52D-1	174	2,330	7.5
htb1-G52D-2	225	6,470	3.5
htb1-G52D-3	297	3,400	8.7
htb1-P102L-1	1,318	13,230	10.0
htb1-P102L-2	762	13,820	5.5
htb1-P102L-3	266	3,350	7.9
WT-4	2,033	10,825	18.8
WT-5	2,838	13,680	20.7
*shf1*Δ-1	61	1,725	3.5
*shf1*Δ-2	47	1,750	2.7
*shf1*Δ-3	77	3,950	1.9
*ubp8*Δ-1	1,099	8,500	13.0
*ubp8*Δ-2	2,399	17,000	14.1
*ubp8*Δ-3	1,871	14,500	13.0
*ubp8*Δ *htb1-G52D*-1	3,054	19,665	15.5
*ubp8*Δ *htb1-G52D*-2	3,878	28,025	13.8
*ubp8*Δ *htb1-G52D*-3	3,252	24,080	13.5
*ubp8*Δ *htb1-P102L*-1	3,047	21,720	14.0
*ubp8*Δ *htb1-P102L*-2	1,814	12,500	15.0
*shf1*Δ *ubp8*Δ *htb1-G52D*-1	1,283	20,595	6.2
*shf1*Δ *ubp8*Δ *htb1-G52D*-2	1,003	20,865	4.8
*shf1*Δ *ubp8*Δ *htb1-P102L*-1	4	59	6.8
*shf1*Δ *ubp8*Δ *htb1-P102L*-2	5	160	3.1

Table S9

Primers used in the qPCR assay

Oligonucleotide	Sequence (5' to 3')	Use
GF85	ATGGTAGATGGAGAAACGGG	Forward primer for qPCR *ars2004*
GF86	CACGGCATCTTTCTTCACGA	Reverse primer for qPCR *ars2004*
GF319	CGTCCTTCCGATGTTGCTTTAACGCATACTC	Reverse primer for qPCR total DNA control in HO-mediated DSB system
GF320	AGACGTATTTGAGTGATAGTGCTCGCTGC	Forward primer for qPCR total DNA control in HO-mediated DSB system
GF321	CCAGTAACTTGTGGGTTCCTTGTAGTCG	Forward primer for qPCR uncut DNA in HO-mediated DSB system
GF322	CGCGCCTTAATTAACCCGGTACAGTAC	Reverse primer for qPCR uncut DNA in HO-mediated DSB system
GF323	TGTTGCGGAAAGCTGAAAGGTACCTG	Forward primer for qPCR DNA 35 bp away from DSB site in HO-mediated DSB system
GF324	CTCGCAGTCTGAGAGAGAACTAGATATCGG	Reverse primer for qPCR DNA 35 bp away from DSB site in HO-mediated DSB system
GF325	CATAAGGTTTGCATACACCGTTGGGTAGG	Forward primer for qPCR DNA 3.1 kb away from DSB site in HO-mediated DSB system
GF326	CGGAAAGAACTTGATTGGATTGATTAACACTCATCC	Reverse primer for qPCR DNA 3.1 kb away from DSB site in HO-mediated DSB system
GF327	TCAAAGCTGCGAAACAACAC	Forward primer for qPCR DNA 9.4 kb away from DSB site in HO-mediated DSB system
GF328	TCGGTGCAGACGATCAATAA	Reverse primer for qPCR DNA 9.4 kb away from DSB site in HO-mediated DSB system
GF329	AGCAGGAGAGAGATATGGAACT	Forward primer for qPCR DNA 20 kb away from DSB site in HO-mediated DSB system
GF330	GGGAAGTCTCCTACTTCAACTT	Reverse primer for qPCR DNA 20 kb away from DSB site in HO-mediated DSB system
GF352	CCATTGAGCACGGTATTGTC	Forward primer for qPCR *act1*$^{+}$
GF353	GAGCCTCAGTCAACAAGCAA	Reverse primer for qPCR *act1*$^{+}$
GF475	GAATGGATAGACCAACGCCAGG	Forward primer for qPCR *brl1*$^{+}$
GF476	TGCTGCACCCGTTTCCATTC	Reverse primer for qPCR *brl1*$^{+}$
GF477	CAAGGCAATGAGCTGAAGGAC	Forward primer for qPCR *brl2*$^{+}$
GF478	GGAGACCGAATTTGTGCTTGAG	Reverse primer for qPCR *brl2*$^{+}$
GF479	CAAAGCTGCAAGTCTGCGTG	Forward primer for qPCR *shf1*$^{+}$
GF480	CAGCTGCATCACTACGTAGACC	Reverse primer for qPCR *shf1*$^{+}$
GF481	TGTTATCATTGGCCCAGCAGAC	Forward primer for qPCR *rhp6*$^{+}$
GF482	TCGCCATTTGCGTACACATTTG	Reverse primer for qPCR *rhp6*$^{+}$
GF630	TTATTCCATAATAACACGACCAATC	Forward primer for qPCR *centromere-I*
GF631	CGACGTTGAAAAGGAAATTAATTG	Reverse primer for qPCR *centromere-I*
GF636	CACTGTTTCAAATGTTAATCTTCG	Forward primer for qPCR *rad9*$^{+}$

GF637	TTCTTGCTATTGGTAGTGACG	Reverse primer for qPCR *rad9*$^+$
GF640	CAAGTGTTGTAGACAGTACATC	Forward primer for qPCR *gcn5*$^+$
GF641	CGTTAGAAACAACACGAAATTG	Reverse primer for qPCR *gcn5*$^+$
GF650	ATGTCTGCTGCTGAAAAGAAAC	Forward primer for qPCR *htb1*$^+$
GF651	CTGAGCAGAAGAAGAATACTTGG	Reverse primer for qPCR *htb1*$^+$
